# Characterising long COVID: a living systematic review

**DOI:** 10.1136/bmjgh-2021-005427

**Published:** 2021-09-27

**Authors:** Melina Michelen, Lakshmi Manoharan, Natalie Elkheir, Vincent Cheng, Andrew Dagens, Claire Hastie, Margaret O'Hara, Jake Suett, Dania Dahmash, Polina Bugaeva, Ishmeala Rigby, Daniel Munblit, Eli Harriss, Amanda Burls, Carole Foote, Janet Scott, Gail Carson, Piero Olliaro, Louise Sigfrid, Charitini Stavropoulou

**Affiliations:** 1 School of Health Sciences, City University of London, London, UK; 2 ISARIC Global Support Centre, Centre for Tropical Medicine and Global Health, University of Oxford, Oxford, UK; 3 Department of Clinical Research, Faculty of Infectious and Tropical Diseases, London School of Hygiene and Tropical Medicine, London, UK; 4 Bristol Medical School, University of Bristol, Bristol, UK; 5 Long Covid Support, Birmingham, UK; 6 Anaesthetic Department, Queen Elizabeth Hospital, Kings Lynn, UK; 7 Julius-Maximilians-Universität Würzburg, Würzburg, Bayern, Germany; 8 Department of Paediatrics and Paediatric Infectious Diseases, Institute of Child’s Health, Sechenov First Moscow State Medical University (Sechenov University), Moscow, Russia; 9 Inflammation, Repair and Development Section, National Heart and Lung Institute, Faculty of Medicine, Imperial College London, London, UK; 10 Research and Clinical Center for Neuropsychiatry, Moscow, Russia; 11 Bodleian Health Care Libraries, University of Oxford, Oxford, UK; 12 Freelance, Soquel, California, USA; 13 MRC-University of Glasgow Centre for Virus Research, University of Glasgow, Glasgow, UK

**Keywords:** COVID-19, public health, systematic review

## Abstract

**Background:**

While it is now apparent clinical sequelae (long COVID) may persist after acute COVID-19, their nature, frequency and aetiology are poorly characterised. This study aims to regularly synthesise evidence on long COVID characteristics, to help inform clinical management, rehabilitation strategies and interventional studies to improve long-term outcomes.

**Methods:**

A living systematic review. Medline, CINAHL (EBSCO), Global Health (Ovid), WHO Global Research on COVID-19 database, LitCovid and Google Scholar were searched till 17 March 2021. Studies including at least 100 people with confirmed or clinically suspected COVID-19 at 12 weeks or more post onset were included. Risk of bias was assessed using the tool produced by Hoy *et al*. Results were analysed using descriptive statistics and meta-analyses to estimate prevalence.

**Results:**

A total of 39 studies were included: 32 cohort, 6 cross-sectional and 1 case–control. Most showed high or moderate risk of bias. None were set in low-income countries and few included children. Studies reported on 10 951 people (48% female) in 12 countries. Most included previously hospitalised people (78%, 8520/10 951). The longest mean follow-up time was 221.7 (SD: 10.9) days post COVID-19 onset. Over 60 physical and psychological signs and symptoms with wide prevalence were reported, most commonly weakness (41%; 95% CI 25% to 59%), general malaise (33%; 95% CI 15% to 57%), fatigue (31%; 95% CI 24% to 39%), concentration impairment (26%; 95% CI 21% to 32%) and breathlessness (25%; 95% CI 18% to 34%). 37% (95% CI 18% to 60%) of patients reported reduced quality of life; 26% (10/39) of studies presented evidence of reduced pulmonary function.

**Conclusion:**

Long COVID is a complex condition with prolonged heterogeneous symptoms. The nature of studies precludes a precise case definition or risk evaluation. There is an urgent need for prospective, robust, standardised, controlled studies into aetiology, risk factors and biomarkers to characterise long COVID in different at-risk populations and settings.

**PROSPERO registration number:**

CRD42020211131.

**Key questions**

**What is already known?**

- A significant number of people continue to describe ongoing symptoms long after the acute phase of COVID-19, often referred to as long COVID.
- Long COVID is a heterogeneous condition with an uncertain prevalence, for which there is currently no precise case definition.

**What are the new findings?**

- The breadth of reported symptoms suggests a complex, heterogeneous condition affecting both those who were hospitalised and those managed in the community.
- Our review identifies weakness (41%; 95% CI 25% to 59%), general malaise (33%; 95% CI 15% to 57%), fatigue (31%; 95% CI 24% to 39%), concentration impairment (26%; 95% CI 21% to 32%) and breathlessness (25%; 95% CI 18% to 34%) as the most common symptoms reported.

**What do the new findings imply?**

- The current evidence base of the clinical spectrum of long COVID is limited, based on heterogenous data, and vulnerable to biases, hence caution should be used when interpreting or generalising the results.
- Our review identifies areas where further long COVID research is critically needed to help characterise long COVID in different populations and define its aetiology, risk factors and biomarkers, as well as the impact on variants of concern and vaccination on long-term outcomes.

## Introduction

SARS-CoV-2 first emerged in December 2019 causing a widespread pandemic. Most people experience asymptomatic or mild-to-moderate acute COVID-19 symptoms, while around 15% of people are estimated to progress to more severe disease requiring hospitalisation and approximately 5% become critically ill.^1^

While the acute phase of the disease was characterised early, there are still limited data on long-term outcomes.^2^ Symptoms of long-lasting COVID-19 sequelae and complications, termed long COVID by people living with long COVID,^3^ have been reported worldwide. Yet the underlying aetiology behind prolonged or fluctuating symptomatology is limited and there is no widely accepted uniformed case definition.^4^ Instead, long COVID has been defined pragmatically as ‘not recovering for several weeks or months following the start of symptoms’.^4^ Others have distinguished between postacute COVID-19, referring to symptoms beyond 3 weeks, and chronic COVID-19, referring to symptoms beyond 12 weeks,^5^ while the National Institute for Health and Care Excellence distinguishes between ongoing symptomatic COVID-19 lasting from 4 to 12 weeks and post COVID-19 syndrome continuing for over 12 weeks.^6^

The number of people living with long COVID is unknown. Attempts to quantify the prevalence of long COVID use different methods, including national surveys and patient-led studies, making it difficult to compare across studies. The UK’s Office for National Statistics has estimated that on average 1 in 5 people have symptoms beyond 5 weeks, while 1 in 10 have symptoms persisting over 12 weeks.^7^ A patient-led survey found that in survival analysis, the chance of full recovery by day 50 was smaller than 20%^8^ and a COVID-19 symptom app study found that 13.3% (558/4182) patients had symptoms lasting 28 days or more, 4.5% (189/4182) patients had symptoms for 8 or more weeks and 2.3% (95/4182) patients had symptoms lasting over 12 weeks.^9^

The symptoms of long COVID are equally ill-defined, with patients describing it as a fluctuating illness of disparate symptoms.^8 10^ Indeed, the National Institute for Health Research has suggested that postacute COVID-19 may consist of several distinct clinical syndromes including: a postintensive care syndrome, chronic fatigue syndrome, long-term COVID-19 syndrome and disease from SARS-CoV-2 inflicted organ damage.^11^ Additionally, even with an expanding knowledge of risk factors in the acute phase, little is currently known on predictive factors for developing long COVID.^9^ Despite suggested classifications, there is yet no clear consensus.

Our early understanding of long COVID has been accumulated from case reports and cross-sectional online survey studies as the pandemic global research focus has largely been on studies of hospitalised patients during the acute phase. As the pandemic progresses, emerging studies have followed up patients to present the fluctuating multiorgan sequelae of acute COVID-19, yet evidence is still scarce. There continues to be a call to further understand and acknowledge this condition by incorporating patient knowledge and experiences, together with standardised studies, exploring underlying aetiologies behind different syndromes.^12 13^

Given the enormous number of people worldwide who have suffered from COVID-19, it is essential to establish a precise categorisation of long COVID. Such categorisation will not only help people better understand their symptoms but also direct research into prevention, treatment and support, ultimately allowing us to understand and prepare to respond to the long-term consequences inflicted by the COVID-19 pandemic. Our review seeks to synthesise and continually update the evidence on the character and prevalence of long COVID.

## Methods

Systematic reviews conducted early during the COVID-19 pandemic soon became redundant due to the rapidity with which new research was released. In recognition of this, many reviewers have moved towards the concept of a ‘living systematic review’ (LSR), which compared with traditional systematic reviews has in-built mechanisms for regular update and renewal.^14 15^ We conducted a ‘living’ systematic review to provide frequently updated evidence on the symptoms and complications of long COVID. This review was developed in collaboration with infectious disease clinicians, public health professionals, information specialists, review methodologists with experience in clinical epidemic research and members of the global Long COVID Support Group, which includes people living with long COVID. This is the first version of this LSR, which will be updated approximately every 6 months as new evidence emerges, using the established protocol and review platform. The updates will be led by the International Severe Acute Respiratory and emerging Infection Consortium (ISARIC) systematic review team in collaboration with members of Long COVID Support. Previous versions will be archived in online supplemental materials. The findings will be disseminated via *BMJ Global Health* and on a dedicated webpage with infographics and a brief summary for lay people and professionals.

10.1136/bmjgh-2021-005427.supp1 Supplementary data

### Protocol registration

This report was structured according to the Preferred Reporting Items for Systematic Reviews and Meta-Analyses statement guidelines.^16^ The protocol was registered with PROSPERO and published in a peer-reviewed journal.^17^

### Search strategy

The following databases were searched: Medline and CINAHL (EBSCO), Global Health (Ovid), WHO Global Research Database on COVID-19 and LitCovid from 1 January 2020 to 17 March 2021. Additionally, we searched Google Scholar on 17 March 2021, screening the first 500 titles. A ‘backwards’ snowball search was conducted of the references of systematic reviews. Full search terms are included in online supplemental file 1. The search terms and inclusion criteria have, for this first version, been designed to cast a wide net and will be modified in line with new evidence, research priorities and clinical and policy needs.

### Eligibility criteria

Peer-reviewed studies were considered eligible if they included at least 100 people with laboratory confirmed and/or clinically diagnosed COVID-19. Without a clear, internationally agreed case definition, we included studies that reported symptoms or outcomes assessed at 12 or more weeks post COVID-19 onset.^6^ There were no language restrictions. Reviews and opinion pieces were excluded. Studies were excluded if they included fewer than 100 participants, to avoid small study effects,^18^ or the follow-up was unclear or less than 12 weeks post onset.

### Screening

Screening was performed independently by two systematic reviewers. Any disagreements were resolved via consensus or a third reviewer. Non-English articles were translated using Google Translate and assessed by a systematic reviewer with good knowledge of the language. The data were managed using the review software Rayyan.^19^

### Data extraction

Data extraction was performed using Microsoft Excel. A data extraction template informed by a previous review^20^ was reviewed, updated and piloted before being finalised. Data extracted included study design, population characteristics, outcomes, prevalence, duration of symptoms and risk factors. Data extraction was performed by one systematic reviewer and checked by a second reviewer. Disagreements were resolved through consensus. To avoid duplication of data in future updates and ensure robustness, data extraction was not performed for non-peer-reviewed preprints.

### Risk of bias assessment

The included studies were assessed for risk of bias using the tool produced by Hoy *et al*^21^ (online supplemental file 2). This assessment checklist is a validated tool for assessing risk of bias in prevalence studies. The checklist has 10 domains for assessing risk of bias, used to calculate a cumulative overall risk of bias for the whole study.

### Data analysis

We undertook individual descriptive analysis for each study. We presented symptom proportions by different settings, as presented in the individual studies: hospitalised, non-hospitalised or a mix of both populations if no subset data were available. Symptoms were broadly grouped into physiological clusters through discussion with clinicians. Proportion of symptoms and its 95% CIs were estimated using the exact method.^22^ If there were two or more studies for each symptom, a meta-analysis was performed using a random intercept logistic regression model with Hartung-Knapp modification due to the heterogeneity and skewed sample sizes.^23 24^ Heterogeneity between estimates was assessed using the I^2^ statistic.^25^ Additional subgroup analysis was conducted to explore the modification of the following factors on proportion of symptoms: hospitalisation, settings, continents and follow-up timing. We also conducted meta-regression analysis on the percentage of females and intensive care unit (ICU) patients where there were more than 10 studies for the symptom. Sensitivity analyses were conducted to examine the impact of high risk of bias studies and statistical methods, Freeman-Tukey double arcsine transformation using inverse variance meta-analysis, on the estimates. Funnel plots were plotted using proportion of the symptom against the precision and sample sizes^22^ where there were more than 10 studies for the symptom to explore risk of publication bias. All analysis and data presentation were performed using metaprop^26^ and ggplot2^27^ in R (V.4.0.5) via RStudio (V.1.3.1093).^28^ The data are presented using a combination of infographics, prepared by a design company (Design Science^29^) and scientific tables to facilitate interpretation by different stakeholders, including non-specialists.

### Patient and public involvement

The study team includes members who have been affected by long-term COVID-19 sequalae, including members of Long COVID Support,^10^ a patient support group with global reach, with approximately 40 000 members.

They actively contributed to the development of the study protocol, to inform the research questions and interpretation and presentation of the findings and to communicate the results to different audiences. The results of this LSR will be disseminated to long COVID patient forums for discussion and feedback to inform research priorities and updates.

## Results

We identified 6459 studies, of which 39 met the inclusion criteria (online supplemental file 3), all of which were published in English. Of these, 32 were included in the meta-analysis. The remaining studies include single symptoms or imaging and diagnostics and are presented narratively.

### Characteristics of included studies

**Figure 1:**
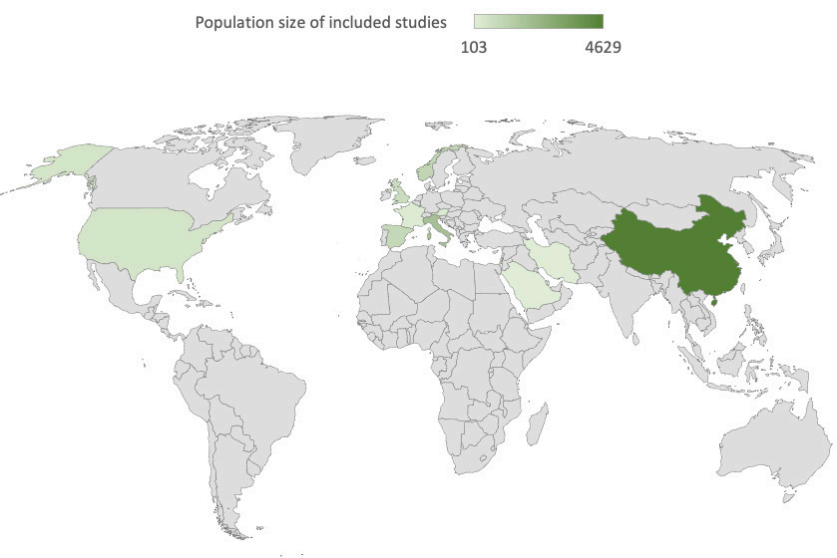
Map of study distribution.

Most studies were set in Europe (62%, 24/39), followed by Asia (23%, 9/39), North America (8%, 3/39) and the Middle East 8% (3/39) (figure 1). There was no study set in a low-middle income country.^30^ Most were cohort studies (82%, 32/39), followed by cross sectional studies (15%, 6/39) and a case–control study (3%, 1/39). These studies present data on 10 951 (range: 100–1733) people in 12 countries, aged from 9 months to 93 years old and 48% (5206/10 951) were females.

The map shows the global distribution of the studies identified and the shading shows the combined studies population size by country.

Most studies included adults, while 10% (4/39) also included children.^31–34^ Only 15% (6/39) of studies reported ethnicity of the participants,^35–40^ but without stratification. Table 1 presents the included study characteristics.

**Table 1:** Study characteristics

Study	Design	Country	Population size	Age (years)	Sex (% female)	COVID-19 confirmation method	Follow-up time (days)	Follow-up timepoint	Follow-up mode
Non-hospitalised									
Hopkins *et al*^58^	Cross sectional	UK	434	Median (range): 40 (19–77)	75	PCR or serological assays (26.3%)	6 months	First survey	Electronic survey
Klein *et al*^47^	Cohort (P)	Israel	103	Mean (SD): 35 (12)	38	PCR (RT-PCR)	6 months	Onset	Phone interview
Petersen *et al*^32^	Cohort (P)	Faroe Islands	180	Mean (SD; range): 39.9 (19.4; 0–93)	54	PCR (RT-PCR)	Mean (SD) 125 (17)	Onset	Phone interview
Stavem *et al*^68^	Crosssectional	Norway	451	Mean (SD): 49.8 (15.2)	56	PCR (RT-PCR)	Median (range): 117 (41–193)	Onset	Outpatient visit and survey
Non-hospitalised and hospitalised									
Parente-Arias *et al*^55^	Cohort (P)	Spain	151	Mean (range): 55.2 (18–88)	65	PCR (RT-PCR)	Mean (SD): 100.5 (3.3)	Admission	Phone interview
Venturelli *et al*^60^	Cohort (P)	Italy	767	Mean (SD): 63 (13.6)	33	PCR (RT-PCR) (94%); serology (5%) Clinician diagnosis (1.2%)	Median (IQR): 105 (84–127)	Onset	Outpatient visit
Anastasio *et al*^41^	Cohort (P)	Italy	379	Median (IQR; range): 56 (49–63; 20–80)	54	PCR (RT-PCR)	Median (IQR): 135 (102–175)	Onset	Outpatient visit
Einvik *et al*^67^	Crosssectional	Norway	538	Mean (SD) 57.7 (14.2) (hospital) 49.6 (15.3)	42 (hospital) 56	PCR (RT-PCR)	Mean (SD): 112 (30) (hospital) 118 (27)	Onset	Outpatient visit and survey
Jacobson *et al*^40^	Cohort (P)	USA	118	Mean (SD): 43.3 (14.4)	47	PCR (RT-PCR)	Mean (SD): 119.3 (33)	Diagnosis	Outpatient visit
Logue *et al*^35^	Cohort (P)	USA	177 21 (C)	Mean (SD): 48 (15.2)	57	Lab confirmed	Median (range): 169 (31–300)	Onset	Electronic survey
Mazza *et al*^70^	Cohort (P)	Italy	226	Mean (SD; range): 58 (12.8; 26–87)	34	PCR (RT-PCR)	Mean (SD): 90 (13.4)	Discharge	Phone interview
Rass *et al*^50^	Cohort (P)	Austria	135	Median (IQR; range) 56 (48–68; 19–87)	39	PCR (RT-PCR)	Median (IQR): 102 (91–110)	Onset	Outpatient visit
Sonnweber *et al*^48^	Cohort (P)	Austria	145	Mean (SD): 57 (14)	43	PCR (RT-PCR)	Mean (SD): 103 (21)	Diagnosis	Outpatient visit
Hospitalised									
Alharthy *et al*^54^	Cohort (P)	Saudi Arabia	127	Mean (SD): 47 (11.38)	21	PCR (RT-PCR)	4 months	Discharge	Outpatient visit
Arnold *et al*^37^	Cohort (P)	UK	110	Median (IQR): 60 (46–73)	38	PCR or radiological diagnosis	Median (IQR): 90 (80–97)	Onset	Outpatient visit
Baricich *et al*^63^	Crosssectional	Italy	204	Mean (SD): 57.9 (12.8)	40	NR	Mean (SD): 124.7 (17.5)	Discharge	Outpatient visit
Bellan *et al*^42^	Cohort (P)	Italy	238	Median (IQR): 61 (50–71)	40	PCR (RT-PCR) (97.5%); bronchoalveolar lavage (0.4%); serology/radiological (2.1%)	3–4 months	Discharge	Outpatient visit
Blanco *et al*^38^	Cohort (P)	Spain	100	Mean (SD) TLco<80: 54.98 (10.72) TLco>80: 54.75 (9.83)	36	PCR (RT-PCR)	Median (IQR): 104 (89.25–126.75)	Onset	Outpatient visit
Doyle *et al*^66^	Cohort (P)	UK	129	Mean: 62 (Cambridge) 56 (London)	31 (Cambridge) 27 (London)	PCR (RT-PCR)	Median (range): 113 (96–138)	Discharge	NR
Garrigues *et al*^65^	Cohort (P)	France	120	Mean (SD): 63.2 (15.7)	38	PCR (RT-PCR)	Mean (SD): 110.9 (11.1)	Admission	Phone interview
Gherlone *et al*^57^	Cohort (P and R)	Italy	122	Median (IQR): 62.5 (53.9–74.1)	25	PCR (RT-PCR)	Median (IQR): 104 (95–132)	Discharge	Outpatient visit
Han *et al*^46^	Cohort (P)	China	114	Mean (SD; range): 54 (12; 24–82)	30	PCR (RT-PCR)	Mean (SD): 175 (20)	Onset	Outpatient visit
Huang *et al*^56^	Cohort (P and R)	China	1733	Median (IQR): 57 (47–65)	48	Lab confirmed	Median (IQR): 186 (175–199)	Onset	Outpatient visit
Zhang *et al*^31^	Cohort (R/S)	China	527	Median (IQR; range): 42.5 (32–54; 0–91)	44	NR	6 months	Discharge	Outpatient visit
Lerum *et al*^61^	Cohort (P)	Norway	103	Median (25th–75th percentile): 59 (49–72)	48	Nasopharyngeal swab	3 months	Discharge	Outpatient visit
Méndez *et al*^49^	Cohort (R/S)	Spain	215	Median (IQR): 55 (47–66)	40	Lab confirmed	Median (IQR): 87 (62–109)	Discharge	Outpatient visit
Nguyen *et al*^33^	Cohort (P)	France	125	Median (IQR; range): 36 (27–48; 16–85)	55	PCR (RT-PCR)	Mean (SD): 221.7 (10.9)	Onset	Phone interview
Nugent *et al*^36^	Cohort (R/S)	USA	182 1430 (C)	Median (IQR): 67.4 (58.3–80.1)	47	PCR (RT-PCR)	Median (IQR): 92.9 (52.5–127.7)	Discharge	Outpatient visit
Qin *et al*^53^	Cohort (P)	China	647	Mean (SD): 58 (15)	56	PCR (RT-PCR)	90	Discharge	Outpatient visit
Qu *et al*^34^	Cohort (P)	China	540	Median (IQR): 47.50 (37–57)	50	PCR (RT-PCR)	3 months	Discharge	Electronic survey
Sibila *et al*^51^	Cohort (P)	Spain	172	Mean (SD): 56.1 (19.8)	43	NR	Mean (SD): 101.5 (19.9)	Discharge	Outpatient visit
Simani *et al*^59^	Cohort (P)	Iran	120	Mean (SD): 54.62 (16.94)	33	PCR or radiological diagnosis	6 months	Discharge	Outpatient visit
Suárez-Robles *et al*^64^	Crosssectional	Spain	134	Mean (SD): 58.53 (18.53)	54	PCR (RT-PCR)	90	Discharge	Phone survey
Sykes *et al*^39^	Cohort (P)	UK	134	Median (range): 58 (25–89)	34	PCR (RT-PCR)	Median (range): 113 (46–167)	Discharge	Outpatient visit
Taboada *et al*^62^	Cross sectional	Spain	183	Mean (SD): 65.9 (14.1)	40	PCR (RT-PCR)	6 months	Discharge	Unstructured interview
Weng *et al*^45^	Cohort (P)	China	117	45.3%≥60 years	44	Viral nucleic acid test	90	Discharge	Phone interview
Xiong *et al*^44^	Cohort (P)	China	538 184 (C)	Median (IQR; range): 52 (41–62; 22–79)	55	PCR (RT-PCR)	Median (IQR; range): 97.0 (95.0–102.0; 91–116)	Discharge	Phone interview
Xu *et al*^43^	Case–control	China	103 27 (C)	Median (IQR) M/M: 56 (45–63) S/C: 61 (55–68)	M/M: 58.8 S/C: 53.6	NR	3 months	Discharge	Outpatient visit
Zhang *et al*^52^	Cohort (P)	China	310	Median (IQR): 51 (31.8–61)	50	PCR (RT-PCR)	Median (IQR): 92.0 (90–100)	Discharge	Outpatient visit

C, control group; M/M, mild/moderate; NR, not reported; P, prospective; PCR, polymerase chain reaction; R, retrospective; RT, Reverse transcription; S/C, severe/critical; TLco, carbon monoxide transfer factor.

Most studies (67%, 26/39) were cohorts of hospitalised patients post discharge, 10% (4/39) followed up people who were not hospitalised, while 23% (9/39) included both (hospitalised and non-hospitalised populations). Of the inclusions in this review, 78% (8520/10 951) were previously hospitalised during the acute COVID-19 phase. Twenty-two studies included people requiring ICU admission during the acute phase.^31 33–35 37 38 40–55^

The longest follow-up period in any study was a mean of 221.7 (SD: 10.9) days post onset. Only 56% (22/39) of studies specified COVID-19 severity,^31 33–35 37 38 40–55^ 31% (12/39) treatment received during the acute phase^36 40 41 45 46 50 53 56–60^ and 62% (24/39) described ventilation support requirements.^36–42 45 46 48–51 53 54 56 57 60–66^ Pre-existing comorbidities were reported in the majority of studies (85%, 33/39), with hypertension and diabetes most commonly documented.^33 35–57 59–63 65 67–69^

### Risk of bias

Overall, 12 studies were assessed as high risk of bias, 22 as moderate risk of bias and 5 as low risk of bias. Most studies had a high risk of bias with regard to the generalisability of their results to the wider population with COVID-19. High risk of bias ratings were most common for external validity, with item 1 (representation of target population) and item 3 (random selection) having the most high risk of bias ratings (online supplemental file 2). Further, the recruitment process and response rates were often not well described and several studies applied different data collection methods. Although many studies applied validated measurement methods to assess participants, most were not designed to detect symptoms arising from COVID-19. Only four studies included a comparative control group.^35 36 43 44^

### Symptoms and signs

**Figure 2:**
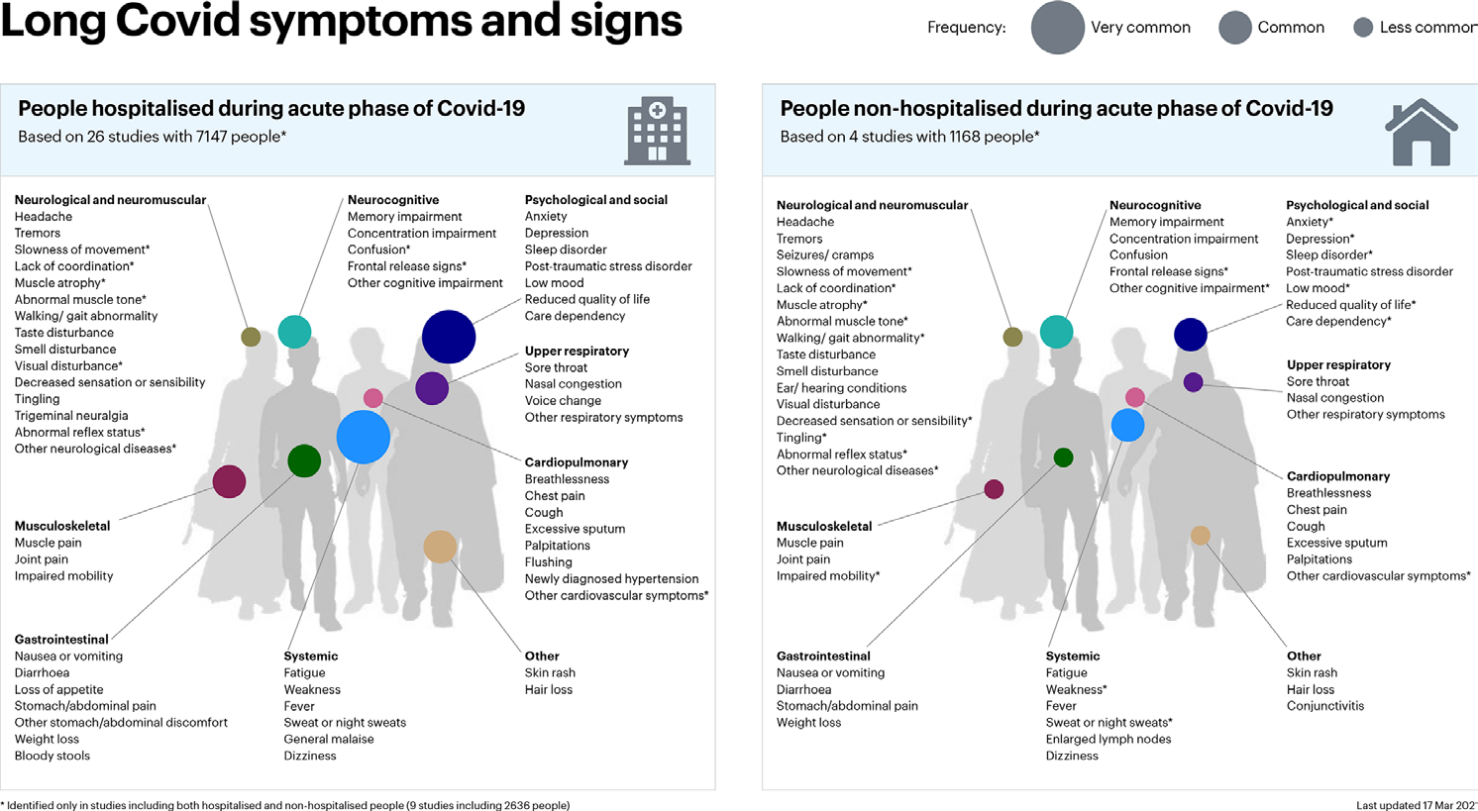
Long COVID signs and symptoms.

Patients suffering from long COVID report a wide range of new or persistent symptoms, in both the hospitalised and non-hospitalised populations. Symptoms were broadly organised into physiological ‘clusters’ for the purpose of presentation and interpretation of this review (figure 2).

The focus of each study included in our analysis varied. Some authors focused solely on a specialty, such as dentistry, or a specific symptom, such as cognition, making comparative analysis difficult. Even among those studies which took a broad approach, the prevalence of symptoms was diverse. Similarly, the prevalence of the more commonly reported symptoms varied markedly.

Within these limitations, we performed a meta-analysis of the most commonly reported symptoms and signs of long COVID. The most commonly described symptoms (with prevalence of 25% or greater) were weakness (41%, 95% CI 25.43 to 59.01), general malaise (33%, 95% CI 14.91 to 57.36), fatigue (31%, 95% CI 23.91 to 39.03), concentration impairment (26%, 95% CI 20.96 to 31.73) and breathlessness (25%, 95% CI 17.86 to 33.97). Across studies, 37% (95% CI 18.43 to 59.93) of patients reported reduced quality of life. Although high I^2^ values (>80%) were observed, they resulted from narrow dispersions in the estimates and well-separated estimates and CIs between studies (online supplemental file 4). The differences between these symptoms and the heterogeneity within them are likely to be, to some extent, due to other factors (eg, study settings, populations and different measurement tools used).

**Figure 3:**
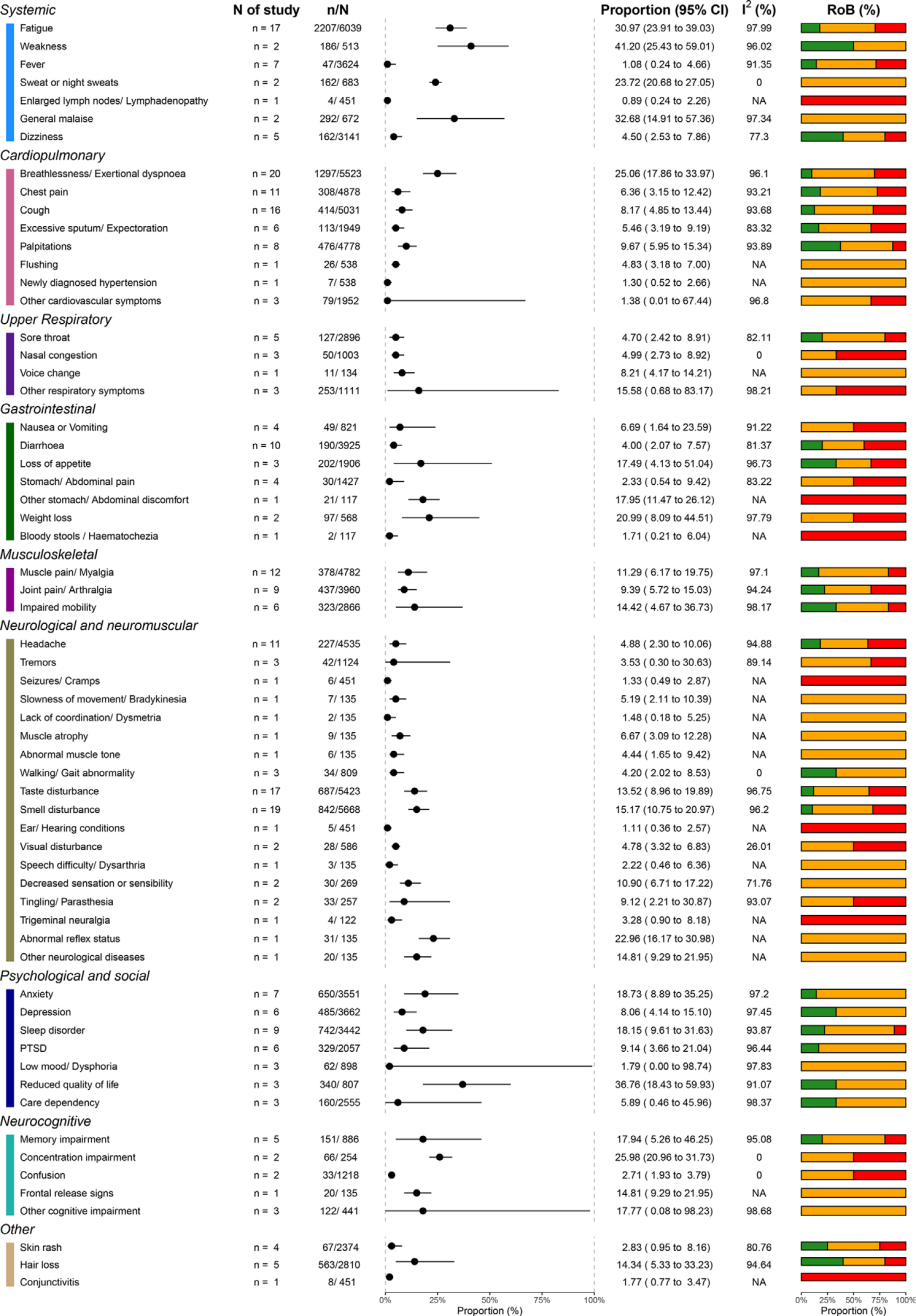
Signs and symptoms in all studies. RoB, risk of bias.

Patients also reported a diverse array of less prevalent symptoms and signs, including sweating, chest pain, sore throat, anxiety and headaches, among others. The prevalence of these symptoms was lower, usually less than 20%. Figure 3 presents the range of documented patient symptoms and signs, including all the studies.

**Figure 4:**
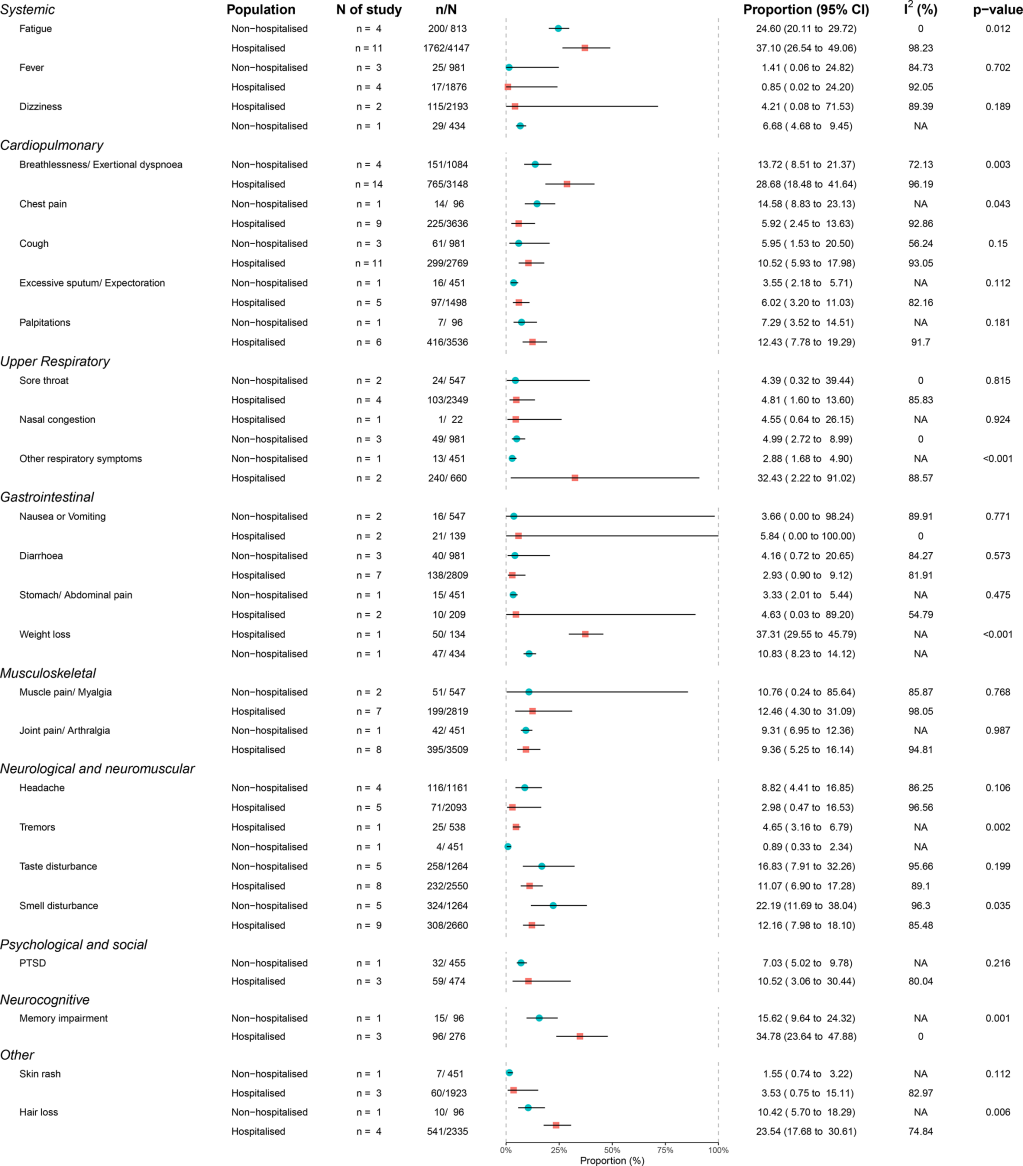
Sign and symptoms in hospitalised and non-hospitalised cohorts. Note: The data on sign and symptoms from studies with data on hospitalised or non-hospitalised cohorts, it does not include studies that included mixed cohorts without subcategorisation. PTSD, post-traumatic stress disorder.

Figure 4 displays these data by population, including the studies that specified hospitalised and non-hospitalised cohorts. We also performed subgroup analysis based on setting (hospitalised vs non-hospitalised) and follow-up time. In several symptoms and signs, the heterogeneity of the results was found to be associated with level of hospitalisation, hospital settings, location of the studies and follow-up timing using subgroup analysis (online supplemental files 5-8). Using meta-regression, the proportion of female patients in the studies was positively associated with headache and smell and taste disturbance (online supplemental file 9), while the proportion of ICU patients in the studies was positively associated with muscle pain (online supplemental file 10). No major difference was found in the sensitivity analyses (online supplemental files 11 and 12). Asymmetries found in the funnel plots suggest reporting biases and poor methodological quality in the included studies (online supplemental file 13).

### Imaging and diagnostics

Multiple studies assessed lung sequelae and respiratory performance through outpatient visits follow-up (49%, 19/39).^31 37–43 46 48 49 51–54 56 60 61 66^ Imaging results were reported in 33% (13/39)^31 37–39 43 46 48 52–54 56 61 66^ of the cohort studies, with one including controls^43^ and one with a population including children.^31^ Authors used heterogenous measurement techniques with an observed tendency towards novel imaging, including artificial intelligence and point‐of‐care ultrasound.^43 54^ Studies found abnormal CT results, including consolidation, reticulation, residual ground glass opacity, interstitial thickening and fibrotic changes. Some of these studies presented comparisons between initial CT findings and those at follow-up, showing improvements in pulmonary clinical measures and radiologic resolutions at follow-up visits.^37 39 46 48 54^ One study assessing thrombotic complications in COVID-19 with a minimum of 90-day follow-up from critical care admission found low rates of hospital-associated venous thromboembolism post discharge.^66^

Pulmonary function tests were reported in 26% (10/39) of studies,^37 38 41–43 48 49 51 53 61^ including spirometry, diffusion capacity, lung volume and exercise tests. These studies found evidence of altered pulmonary function, most frequently significant reduction of carbon monoxide transfer factor.

One study assessed kidney function in people with COVID-19-associated acute kidney injury (AKI) compared with people with non-COVID-19-associated AKI, found that COVID-19-related AKI was associated with decreased kidney recovery during outpatient follow-up.^36^

### Risk factors

Exploring the literature, we sought to produce a meta-analysis of risk factors for long COVID. We found a considerable diversity of reported risk factors, including age, sex, comorbidities, ethnicity and severity of the acute phase.

Several cohorts (64%, 25/39) assessed whether there was an association between the severity of initial COVID-19, including symptom load, level of hospital care, need for mechanical ventilation and the risk of persisting sequelae. An association between female gender and long COVID risk has also been noted in longitudinal studies (20.5%, 8/39), as has the association between presence of comorbidity,^40 55 57 63 68 70^ increasing age^32 34 50 55 62 63^ and minority ethnicity,^40 67^ with long COVID and long COVID risk.

The limitations of the existing evidence base and inconsistency of reported findings preclude confident conclusions at this time. Instead, we have summarised the reported significant associations to date (online supplemental file 14) and suggest that these associations be explored in prospective controlled trials.

## Discussion

Our work represents the most comprehensive review of evidence regarding long COVID yet produced. Accurate to 17 March 2021, this LSR captures the breadth of persistent symptoms reported in 39 studies, including over 10 000 people. These data suggest long COVID is a syndrome affecting both previously hospitalised and non-hospitalised people, characterised by marked fatigue, weakness, general malaise, breathlessness and concentration impairment lasting for a prolonged period of time. Besides these common symptoms, there is a diverse array of secondary symptoms. The findings in this review show symptoms and prevalence aligned to current knowledge on long COVID. The Office for National Statistics (ONS) Cohort Study, including control participants, reports the most common symptoms persisting for 12 or more weeks included fatigue (8.3%), headache (7.2%), cough (7%) and myalgia (5.6%).^7^

A deeper understanding of long COVID is currently prevented by the limitations of the published literature. The studies included in our review were highly heterogeneous due to differences in their study designs, settings, populations, follow-up time and symptom ascertainment methods. In addition, studies used inconsistent terminology describing symptoms and limited details and stratification on pre-existing comorbidities, the severity of COVID-19 and treatment methods. This inconsistency and limited reporting partly explain the high degree of variability observed. The lack of case–control studies prevent a direct attribution of symptoms solely to COVID-19; larger prospective studies with matched control groups are needed. We note that there are large, robust prospective cohort studies of hospitalised patients^71^ and non-hospitalised people.^72^ Simultaneously, qualitative studies are ongoing to better explore the long COVID patient experience.^73^

The findings have identified several research gaps and priorities. The majority of long COVID cohorts were conducted in Western Europe on patients recently discharged from hospital. There is a paucity of evidence on the long-term effects of COVID-19 in low-to-middle income countries and in people who were not hospitalised. Similarly, there were no studies identified focusing on children, despite evidence showing that children and young people are also affected by long COVID.^74^ Additionally, no study stratified by ethnicity, an important risk factor for the acute phase.

Our review also highlights a need for standardised and validated COVID-19 research tools to harmonise data collection, improve quality and reduce reporting variability. For instance, fatigue is one of the most commonly reported symptoms of long COVID. However, the symptom alone is not clearly defined and it is open to different interpretations, hence it requires a validated tool such as the Visual Analogue Scale, graded fatigue scale for robust, objective and comparative analysis. ISARIC has developed open access research tools available to sites globally to facilitate standardisation of data collection, analysis and interpretation for adults and children of an age.^75^ We support the broader use of this tool as well as initiatives to standardise outcome measures for long COVID.

Similarly, our study highlights the need for further research to refine the many circulating interim case definitions and precisely characterise long COVID, including the potential impacts of variants of concern and vaccination on long COVID.

As this is an LSR, emerging themes from this first version will inform future updates. The LSR will be updated periodically, as new research is published internationally, in order to provide relevant up to date information for clinicians, patients, researchers, policy-makers and health-service commissioners. Version changes will be identified and previous reports will be archived.

## Conclusion

This LSR summarises published evidence on the spectrum of long-term COVID-19-associated symptoms and sequelae (as of 17 March 2021). It is clear that long COVID affects different populations, with a wide range of symptomatology. Our findings suggest this multiorgan syndrome is characterised by fatigue, weakness, malaise, breathlessness and concentration impairment, among other less frequent symptoms. Currently, the strength of the available evidence is limited and prone to bias. The long-term effects of COVID-19, in both hospitalised and non-hospitalised individuals, including children and at-risk populations, should be a priority for future research using standardised and controlled study designs. Robust research is needed to characterise and define long COVID and identify risk factors and underlying aetiology, in order to inform prevention, rehabilitation, clinical and public health management to improve recovery and long-term COVID-19 outcomes. This LSR will be updated approximately every 6 months as new evidence emerges for up to 2 years.

## Data Availability

All data relevant to the study are included in the article or uploaded as supplementary information.
