# Supplementary material for: Characterising long COVID: a living systematic review
Source: BMJ Glob Health. 2021 Sep 27;6(9):e005427. doi: 10.1136/bmjgh-2021-005427 (PMC8478580; doi:10.1136/bmjgh-2021-005427)
Supplement: Supplementary data [file bmjgh-2021-005427supp001.pdf]

Table of Contents

Supplement 1: Search strategy summary ... 2

Appendix 1 ... 4

Appendix 2 ... 4

Appendix 3 ... 5

Supplement 2: Risk of bias assessment ... 10

Supplement 3: PRISMA diagram ... 11

Supplement 4: Individual forest plots in main results ... 12

Supplement 5: Subgroup analysis: hospitalisation ... 24

Supplement 6: Subgroup analysis: Setting ... 28

Supplement 7: Subgroup analysis: Continents ... 31

Supplement 8: Subgroup analysis: Follow-up timing ... 35

Supplement 9: Meta-regression: % Female ... 37

Supplement 10: Meta-regression: % ICU patients ... 40

Supplement 11: Sensitivity analysis: versus removing high risk of bias studies ... 41

Supplement 12: Sensitivity analysis: versus statistical methods ... 43

Supplement 13: Funnel plots ... 45

Supplement 14: Risk factors ... 54

Michelen M, *et al. BMJ Global Health* 2021; 6:e005427. doi: 10.1136/bmjgh-2021-005427

Supplement 1: Search strategy summary

Database	Strategy	Results (16/3/2021)
**Medline (EBSCOhost)**	(COVID-19 OR covid OR SARS-CoV-2. ab)<br>AND<br>(symptom* OR "clinical features" OR signs OR characteristic* OR sequela* OR complication*.ab)<br>AND<br>("long-term Covid" OR long-term OR consequence* OR "long-term impact" OR "long-term effect" OR "post-acute" OR long-tail OR persist* OR "chronic-COVID" OR "long-COVID" OR post-discharge OR postdischarge OR "prolonged symptom" OR "long-haul" .ab)<br>**Limits**: 2020-	1952
**CINAHL (EBSCOhost)**	(COVID-19 OR covid OR SARS-CoV-2. ab)<br>AND<br>(symptom* OR "clinical features" OR signs OR characteristic* OR sequela* OR complication*.ab)<br>AND<br>("long-term Covid" OR long-term OR consequence* OR "long-term impact" OR "long-term effect" OR "post-acute" OR long-tail OR persist* OR "chronic-COVID" OR "long-COVID" OR post-discharge OR postdischarge OR "prolonged symptom" OR "long-haul" .ab)<br>**Limits**: 2020-	384
**Global Health**	(COVID-19 or covid or SARS-CoV-2)<br>AND<br>(symptom* or "clinical features" or signs or characteristic* or sequelae or complication*)<br>AND<br>((("long-term Covid" or long-term) adj2 consequence*) or "long-term impact" or "long-term effect" or "post-acute" or long-tail or persist* or "chronic-COVID" or "long-COVID" or post-discharge or postdischarge or "prolonged symptom" or "long-haul")).ab.<br>**Limits**: 2020-	35
**WHO COVID-19 (WHO COVID, ELSEVIER and Lanzhou University/CNKI)**	tw:((ab:(covid-19 OR covid OR sars-cov-2)) AND (ab:(symptom OR "clinical features" OR signs OR characteristic OR sequela OR complication)) AND (ab:("long-term Covid" OR "long-term consequence" OR "long-term impact" OR "long-term effect" OR "post-acute" OR long-tail OR persist* OR "chronic-COVID" OR "long-COVID" OR post-discharge OR postdischarge OR "prolonged symptom" OR "long-haul"))) AND db:("COVIDWHO" OR "ELSEVIER" OR "CNKI_Lanzhou")	195

Michelen M, *et al. BMJ Global Health* 2021; 6:e005427. doi: 10.1136/bmjgh-2021-005427

**Lit Covid**	(“persistent symptoms” OR “after covid-19 infection").ti,ab,kw<br>OR<br>((“outcomes “ OR “characteristics” OR “features” OR “symptoms” OR “inflammation” OR “function” OR “complications” OR “syndrome” OR “manifestation”) ADJ10 (“long-haul” OR “recovery” OR “recovered” OR “recovering” OR “survivors” OR “post-discharge” OR “postdischarge” OR “discharge” OR “persisting” OR “prolonged” OR “long-term” OR “after admission” OR “post-COVID-19” OR “post-COVID”)).ti,ab.<br>OR<br>((“outcomes “ OR “characteristics” OR “features” OR “symptoms” OR “inflammation” OR “function” OR “complications” OR “syndrome” OR “manifestation”) ADJ/10 (“after admission” OR “after hospital” OR “after hospitalisation" OR “after hospitalization" OR “after COVID-19” OR “after SARS-CoV-2")).ti,ab.	1432
**Google Scholar**	post COVID after discharge persistent symptom	1000
**Ovid Embase (top-up) [17 Mar 2021]**	See Appendix 1<br>**Limit**: 2020-	483
**Ovid Medline (top-up) [17 Mar 2021]**	See Appendix 2<br>**Limit**: 2020-	336
**WHO (top-up)<br>[19 Mar 2021]<br>(excluded: PREPRINT-BIORXVI, PREPRINT-MEDRXVI, PREPRINT-other preprint, ITCRP)**	See Appendix 3	340

Appendix 1

Database(s): **Embase** 1974 to 2021 March 17

#	Searches	Results
1	(long* adj3 (covid* or ncov* or novel coronavirus or novel betacoronavirus or sars-ncov-2 or sars-cov-2)).mp.	462
2	(persist* adj3 (covid* or ncov* or novel coronavirus or novel betacoronavirus or sars-ncov-2 or sars-cov-2)).mp.	265
3	(chronic adj3 (covid* or ncov* or novel coronavirus or novel betacoronavirus or sars-ncov-2 or sars-cov-2)).mp.	191
4	((long term or long-term or longterm) adj3 effect* adj3 (covid* or ncov* or novel coronavirus or novel betacoronavirus or sars-ncov-2 or sars-cov-2)).mp.	59
5	(sequela* adj3 (covid* or ncov* or novel coronavirus or novel betacoronavirus or sars-ncov-2 or sars-cov-2)).mp.	150
6	((post acute or post-acute or postacute) adj3 (covid* or ncov* or novel coronavirus or novel betacoronavirus or sars-ncov-2 or sars-cov-2)).mp.	38
7	((longhaul* or long haul* or long-haul*) adj3 (covid* or ncov* or novel coronavirus or novel betacoronavirus or sars-ncov-2 or sars-cov-2)).mp.	18
8	((post-covid* or postcovid*) adj2 (syndrome or condition)).mp.	32
9	1 or 2 or 3 or 4 or 5 or 6 or 7 or 8	1123
10	symptom/	151132
11	symptom assessment/	8515
12	exp complication/	1237024
13	(symptom* or "clinical feature*" or signs or characteristic* or sequela* or complication*).mp.	6906139
14	exp physical disease by body function/	9306422
15	10 or 11 or 12 or 13 or 14	13028765
16	9 and 15	748
17	limit 16 to yr="2020"	483

Appendix 2

Database(s): **Ovid MEDLINE(R) and Epub Ahead of Print, In-Process, In-Data-Review & Other Non-Indexed Citations and Daily** 1946 to March 17, 2021

#	Searches	Results
1	(long* adj3 (covid* or ncov* or novel coronavirus or novel betacoronavirus or sars-ncov-2 or sars-cov-2)).mp.	507
2	(persist* adj3 (covid* or ncov* or novel coronavirus or novel betacoronavirus or sars-ncov-2 or sars-cov-2)).mp.	273
3	(chronic adj3 (covid* or ncov* or novel coronavirus or novel betacoronavirus or sars-ncov-2 or sars-cov-2)).mp.	173
4	((long term or long-term or longterm) adj3 effect* adj3 (covid* or ncov* or novel coronavirus or novel betacoronavirus or sars-ncov-2 or sars-cov-2)).mp.	62
5	(sequela* adj3 (covid* or ncov* or novel coronavirus or novel betacoronavirus or sars-ncov-2 or sars-cov-2)).mp.	159
6	((post acute or post-acute or postacute) adj3 (covid* or ncov* or novel coronavirus or novel betacoronavirus or sars-ncov-2 or sars-cov-2)).mp.	66
7	((longhaul* or long haul* or long-haul*) adj3 (covid* or ncov* or novel coronavirus or novel betacoronavirus or sars-ncov-2 or sars-cov-2)).mp.	25
8	((post-covid* or postcovid*) adj2 (syndrome or condition)).mp.	36

9	1 or 2 or 3 or 4 or 5 or 6 or 7 or 8	1179
10	exp "signs and symptoms"/	2127623
11	(symptom* or "clinical feature*" or signs or characteristic* or sequela* or complication*).tw.	3644236
12	10 or 11	5362470
13	9 and 12	591
14	limit 13 to yr="2020"	336

Appendix 3

There are only three strings of syntax:

1. keywords and phrases associated with “long COVID”
2. keywords and phrases associated with “hospitalisation” and “quarantine”
3. keywords and phrases associated with “symptoms” and “complications”

The combinations of 1 AND 3 and 2 AND 3 were used to search in the combinations of title, abstract and TW (title + abstract + subjects) in the database. These consist of 18 searches as follows:

#	Syntax	Hits
1	(**ti**:(post-COVID* OR post-nCov* OR "post novel coronavirus" OR "post novel betacoronavirus" OR "post SARS-nCoV-2" OR "post SARS-CoV-2" OR postCOVID* OR "post nCov*" OR "long* COVID*" OR "long* nCov*" OR "long* novel coronavirus" OR "long* novel betacoronavirus" OR "long* SARS-nCoV-2" OR "long* SARS-CoV-2" OR "long-term COVID*" OR "long-term nCov*" OR "long-term novel coronavirus" OR "long-term novel betacoronavirus" OR "long-term SARS-nCoV-2" OR "long-term SARS-CoV-2" OR "long-term COVID*" OR "longterm COVID*" OR "post acute COVID*" OR "postacute COVID*" OR "longhaul* COVID*" OR "long haul* COVID*" OR "long-haul* COVID*" OR "chronic* COVID*" OR "prolonged* COVID*" OR "presist* COVID*" OR "long-term nCov*" OR "longterm nCov*" OR "post acute nCov*" OR "postacute nCov*" OR "longhaul* nCov*" OR "long haul* nCov*" OR "long-haul* nCov*" OR "chronic* nCov*" OR "prolonged* nCov*" OR "presist* nCov*" OR "long-term novel coronavirus" OR "longterm novel coronavirus" OR "post acute novel coronavirus" OR "postacute novel coronavirus" OR "longhaul* novel coronavirus" OR "long haul* novel coronavirus" OR "long-haul* novel coronavirus" OR "chronic* novel coronavirus" OR "prolonged* novel coronavirus" OR "presist* novel coronavirus" OR "long-term novel betacoronavirus" OR "longterm novel betacoronavirus" OR "post acute novel betacoronavirus" OR "postacute novel betacoronavirus" OR "longhaul* novel betacoronavirus" OR "long haul* novel betacoronavirus" OR "long-haul* novel betacoronavirus" OR "chronic* novel betacoronavirus" OR "prolonged* novel betacoronavirus" OR "presist* novel betacoronavirus" OR "long-term SARS-nCoV-2" OR "longterm SARS-nCoV-2" OR "post acute SARS-nCoV-2" OR "postacute SARS-nCoV-2" OR "longhaul* SARS-nCoV-2" OR "long haul* SARS-nCoV-2" OR "long-haul* SARS-nCoV-2" OR "chronic* SARS-nCoV-2" OR "prolonged* SARS-nCoV-2" OR "presist* SARS-nCoV-2" OR "long-term SARS-CoV-2" OR "longterm SARS-CoV-2" OR "post acute SARS-CoV-2" OR "postacute SARS-CoV-2" OR "longhaul* SARS-CoV-2" OR "long haul* SARS-CoV-2" OR "long-haul* SARS-CoV-2" OR "chronic* SARS-CoV-2" OR "prolonged* SARS-CoV-2" OR "presist* SARS-CoV-2") **AND ti**:("clinical feature*" OR abnormal* OR characteristic* OR complication* OR condition* OR convalescence OR disorder* OR dysfunction OR illness* OR impair* OR inflammation OR manifestation OR outcome* OR prevalence OR problem* OR sequela* OR sign* OR symptom* OR syndrome))	135
2	(**ti**:("after admission" OR "after discharg*" OR "after hospital*" OR "after isolat*" OR "after quarantine" OR "after self isolat*" OR "after self-isolat*" OR "after self-quarantine" OR "post admission" OR "post discharg*" OR "post hospital*" OR "post isolat*" OR "post quarantine" OR "post self isolat*" OR "post self-isolat*" OR "post self-quarantine") **AND ti**:("clinical feature*" OR abnormal* OR characteristic* OR complication* OR condition* OR convalescence OR disorder* OR dysfunction OR illness* OR impair* OR inflammation OR manifestation OR outcome* OR prevalence OR problem* OR sequela* OR sign* OR symptom* OR syndrome))	11

Michelen M, *et al. BMJ Global Health* 2021; 6:e005427. doi: 10.1136/bmjgh-2021-005427

3	(**ti**:(post-COVID* OR post-nCov* OR "post novel coronavirus" OR "post novel betacoronavirus" OR "post SARS-nCoV-2" OR "post SARS-CoV-2" OR postCOVID* OR "post nCov*" OR "long* COVID*" OR "long* nCov*" OR "long* novel coronavirus" OR "long* novel betacoronavirus" OR "long* SARS-nCoV-2" OR "long* SARS-CoV-2" OR "long-term COVID*" OR "long-term nCov*" OR "long-term novel coronavirus" OR "long-term novel betacoronavirus" OR "long-term SARS-nCoV-2" OR "long-term SARS-CoV-2" OR "long-term COVID*" OR "longterm COVID*" OR "post acute COVID*" OR "postacute COVID*" OR "longhaul* COVID*" OR "long haul* COVID*" OR "long-haul* COVID*" OR "chronic* COVID*" OR "prolonged* COVID*" OR "presist* COVID*" OR "long-term nCov*" OR "longterm nCov*" OR "post acute nCov*" OR "postacute nCov*" OR "longhaul* nCov*" OR "long haul* nCov*" OR "long-haul* nCov*" OR "chronic* nCov*" OR "prolonged* nCov*" OR "presist* nCov*" OR "long-term novel coronavirus" OR "longterm novel coronavirus" OR "post acute novel coronavirus" OR "postacute novel coronavirus" OR "longhaul* novel coronavirus" OR "long haul* novel coronavirus" OR "long-haul* novel coronavirus" OR "chronic* novel coronavirus" OR "prolonged* novel coronavirus" OR "presist* novel coronavirus" OR "long-term novel betacoronavirus" OR "longterm novel betacoronavirus" OR "post acute novel betacoronavirus" OR "postacute novel betacoronavirus" OR "longhaul* novel betacoronavirus" OR "long haul* novel betacoronavirus" OR "long-haul* novel betacoronavirus" OR "chronic* novel betacoronavirus" OR "prolonged* novel betacoronavirus" OR "presist* novel betacoronavirus" OR "long-term SARS-nCoV-2" OR "longterm SARS-nCoV-2" OR "post acute SARS-nCoV-2" OR "postacute SARS-nCoV-2" OR "longhaul* SARS-nCoV-2" OR "long haul* SARS-nCoV-2" OR "long-haul* SARS-nCoV-2" OR "chronic* SARS-nCoV-2" OR "prolonged* SARS-nCoV-2" OR "presist* SARS-nCoV-2" OR "long-term SARS-CoV-2" OR "longterm SARS-CoV-2" OR "post acute SARS-CoV-2" OR "postacute SARS-CoV-2" OR "longhaul* SARS-CoV-2" OR "long haul* SARS-CoV-2" OR "long-haul* SARS-CoV-2" OR "chronic* SARS-CoV-2" OR "prolonged* SARS-CoV-2" OR "presist* SARS-CoV-2") **AND ab**:("clinical feature*" OR abnormal* OR characteristic* OR complication* OR condition* OR convalescence OR disorder* OR dysfunction OR illness* OR impair* OR inflammation OR manifestation OR outcome* OR prevalence OR problem* OR sequela* OR sign* OR symptom* OR syndrome))	352
4	(**ti**:("after admission" OR "after discharg*" OR "after hospital*" OR "after isolat*" OR "after quarantine" OR "after self isolat*" OR "after self-isolat*" OR "after self-quarantine" OR "post admission" OR "post discharg*" OR "post hospital*" OR "post isolat*" OR "post quarantine" OR "post self isolat*" OR "post self-isolat*" OR "post self-quarantine") **AND ab**:("clinical feature*" OR abnormal* OR characteristic* OR complication* OR condition* OR convalescence OR disorder* OR dysfunction OR illness* OR impair* OR inflammation OR manifestation OR outcome* OR prevalence OR problem* OR sequela* OR sign* OR symptom* OR syndrome))	35
5	(**ti**:(post-COVID* OR post-nCov* OR "post novel coronavirus" OR "post novel betacoronavirus" OR "post SARS-nCoV-2" OR "post SARS-CoV-2" OR postCOVID* OR "post nCov*" OR "long* COVID*" OR "long* nCov*" OR "long* novel coronavirus" OR "long* novel betacoronavirus" OR "long* SARS-nCoV-2" OR "long* SARS-CoV-2" OR "long-term COVID*" OR "long-term nCov*" OR "long-term novel coronavirus" OR "long-term novel betacoronavirus" OR "long-term SARS-nCoV-2" OR "long-term SARS-CoV-2" OR "long-term COVID*" OR "longterm COVID*" OR "post acute COVID*" OR "postacute COVID*" OR "longhaul* COVID*" OR "long haul* COVID*" OR "long-haul* COVID*" OR "chronic* COVID*" OR "prolonged* COVID*" OR "presist* COVID*" OR "long-term nCov*" OR "longterm nCov*" OR "post acute nCov*" OR "postacute nCov*" OR "longhaul* nCov*" OR "long haul* nCov*" OR "long-haul* nCov*" OR "chronic* nCov*" OR "prolonged* nCov*" OR "presist* nCov*" OR "long-term novel coronavirus" OR "longterm novel coronavirus" OR "post acute novel coronavirus" OR "postacute novel coronavirus" OR "longhaul* novel coronavirus" OR "long haul* novel coronavirus" OR "long-haul* novel coronavirus" OR "chronic* novel coronavirus" OR "prolonged* novel coronavirus" OR "presist* novel coronavirus" OR "long-term novel betacoronavirus" OR "longterm novel betacoronavirus" OR "post acute novel betacoronavirus" OR "postacute novel betacoronavirus" OR "longhaul* novel betacoronavirus" OR "long haul* novel betacoronavirus" OR "long-haul* novel betacoronavirus" OR "chronic* novel betacoronavirus" OR "prolonged* novel betacoronavirus" OR "presist* novel betacoronavirus" OR "long-term SARS-nCoV-2" OR "longterm SARS-nCoV-2" OR "post acute SARS-nCoV-2" OR "postacute SARS-nCoV-2" OR "longhaul* SARS-nCoV-2" OR "long haul* SARS-nCoV-2" OR "long-haul* SARS-nCoV-2" OR "chronic* SARS-nCoV-2" OR "prolonged* SARS-nCoV-2" OR "presist* SARS-nCoV-2" OR "long-term SARS-CoV-2" OR "longterm SARS-CoV-2" OR "post acute SARS-CoV-2" OR "postacute SARS-CoV-2" OR "longhaul* SARS-CoV-2" OR "long haul* SARS-CoV-2" OR "long-haul* SARS-CoV-2" OR "chronic* SARS-CoV-2" OR "prolonged* SARS-CoV-2" OR "presist* SARS-CoV-2") **AND tw**:("clinical feature*" OR abnormal* OR characteristic* OR complication* OR condition* OR convalescence OR disorder* OR dysfunction OR illness* OR impair* OR inflammation OR manifestation OR outcome* OR prevalence OR problem* OR sequela* OR sign* OR symptom* OR syndrome))	508
6	(**ti**:("after admission" OR "after discharg*" OR "after hospital*" OR "after isolat*" OR "after quarantine" OR "after self isolat*" OR "after self-isolat*" OR "after self-quarantine" OR "post admission" OR "post discharg*" OR "post hospital*" OR "post isolat*" OR "post quarantine" OR "post self isolat*" OR "post self-isolat*" OR "post self-quarantine") **AND tw**:("clinical feature*" OR abnormal* OR characteristic* OR complication* OR condition* OR convalescence OR disorder* OR dysfunction OR illness* OR impair* OR inflammation OR manifestation OR outcome* OR prevalence OR problem* OR sequela* OR sign* OR symptom* OR syndrome))	38
7	(**ab**:(post-COVID* OR post-nCov* OR "post novel coronavirus" OR "post novel betacoronavirus" OR "post SARS-nCoV-2" OR "post SARS-CoV-2" OR postCOVID* OR "post nCov*" OR "long* COVID*" OR "long* nCov*" OR "long* novel coronavirus" OR "long* novel betacoronavirus" OR "long* SARS-nCoV-2" OR "long* SARS-CoV-2" OR "long-term COVID*"	159

	OR "long-term nCov*" OR "long-term novel coronavirus" OR "long-term novel betacoronavirus" OR "long-term SARS-nCoV-2" OR "long-term SARS-CoV-2" OR "long-term COVID*" OR "longterm COVID*" OR "post acute COVID*" OR "postacute COVID*" OR "longhaul* COVID*" OR "long haul* COVID*" OR "long-haul* COVID*" OR "chronic* COVID*" OR "prolonged* COVID*" OR "presist* COVID*" OR "long-term nCov*" OR "longterm nCov*" OR "post acute nCov*" OR "postacute nCov*" OR "longhaul* nCov*" OR "long haul* nCov*" OR "long-haul* nCov*" OR "chronic* nCov*" OR "prolonged* nCov*" OR "presist* nCov*" OR "long-term novel coronavirus" OR "longterm novel coronavirus" OR "post acute novel coronavirus" OR "postacute novel coronavirus" OR "longhaul* novel coronavirus" OR "long haul* novel coronavirus" OR "long-haul* novel coronavirus" OR "chronic* novel coronavirus" OR "prolonged* novel coronavirus" OR "presist* novel coronavirus" OR "long-term novel betacoronavirus" OR "longterm novel betacoronavirus" OR "post acute novel betacoronavirus" OR "postacute novel betacoronavirus" OR "longhaul* novel betacoronavirus" OR "long haul* novel betacoronavirus" OR "long-haul* novel betacoronavirus" OR "chronic* novel betacoronavirus" OR "prolonged* novel betacoronavirus" OR "presist* novel betacoronavirus" OR "long-term SARS-nCoV-2" OR "longterm SARS-nCoV-2" OR "post acute SARS-nCoV-2" OR "postacute SARS-nCoV-2" OR "longhaul* SARS-nCoV-2" OR "long haul* SARS-nCoV-2" OR "long-haul* SARS-nCoV-2" OR "chronic* SARS-nCoV-2" OR "prolonged* SARS-nCoV-2" OR "presist* SARS-nCoV-2" OR "long-term SARS-CoV-2" OR "longterm SARS-CoV-2" OR "post acute SARS-CoV-2" OR "postacute SARS-CoV-2" OR "longhaul* SARS-CoV-2" OR "long haul* SARS-CoV-2" OR "long-haul* SARS-CoV-2" OR "chronic* SARS-CoV-2" OR "prolonged* SARS-CoV-2" OR "presist* SARS-CoV-2") **AND ti**:("clinical feature*" OR abnormal* OR characteristic* OR complication* OR condition* OR convalescence OR disorder* OR dysfunction OR illness* OR impair* OR inflammation OR manifestation OR outcome* OR prevalence OR problem* OR sequela* OR sign* OR symptom* OR syndrome))	
8	(**ab**:("after admission" OR "after discharg*" OR "after hospital*" OR "after isolat*" OR "after quarantine" OR "after self isolat*" OR "after self-isolat*" OR "after self-quarantine" OR "post admission" OR "post discharg*" OR "post hospital*" OR "post isolat*" OR "post quarantine" OR "post self isolat*" OR "post self-isolat*" OR "post self-quarantine") **AND ti**:("clinical feature*" OR abnormal* OR characteristic* OR complication* OR condition* OR convalescence OR disorder* OR dysfunction OR illness* OR impair* OR inflammation OR manifestation OR outcome* OR prevalence OR problem* OR sequela* OR sign* OR symptom* OR syndrome))	202
9	(**ab**:(post-COVID* OR post-nCov* OR "post novel coronavirus" OR "post novel betacoronavirus" OR "post SARS-nCoV-2" OR "post SARS-CoV-2" OR postCOVID* OR "post nCov*" OR "long* COVID*" OR "long* nCov*" OR "long* novel coronavirus" OR "long* novel betacoronavirus" OR "long* SARS-nCoV-2" OR "long* SARS-CoV-2" OR "long-term COVID*" OR "long-term nCov*" OR "long-term novel coronavirus" OR "long-term novel betacoronavirus" OR "long-term SARS-nCoV-2" OR "long-term SARS-CoV-2" OR "long-term COVID*" OR "longterm COVID*" OR "post acute COVID*" OR "postacute COVID*" OR "longhaul* COVID*" OR "long haul* COVID*" OR "long-haul* COVID*" OR "chronic* COVID*" OR "prolonged* COVID*" OR "presist* COVID*" OR "long-term nCov*" OR "longterm nCov*" OR "post acute nCov*" OR "postacute nCov*" OR "longhaul* nCov*" OR "long haul* nCov*" OR "long-haul* nCov*" OR "chronic* nCov*" OR "prolonged* nCov*" OR "presist* nCov*" OR "long-term novel coronavirus" OR "longterm novel coronavirus" OR "post acute novel coronavirus" OR "postacute novel coronavirus" OR "longhaul* novel coronavirus" OR "long haul* novel coronavirus" OR "long-haul* novel coronavirus" OR "chronic* novel coronavirus" OR "prolonged* novel coronavirus" OR "presist* novel coronavirus" OR "long-term novel betacoronavirus" OR "longterm novel betacoronavirus" OR "post acute novel betacoronavirus" OR "postacute novel betacoronavirus" OR "longhaul* novel betacoronavirus" OR "long haul* novel betacoronavirus" OR "long-haul* novel betacoronavirus" OR "chronic* novel betacoronavirus" OR "prolonged* novel betacoronavirus" OR "presist* novel betacoronavirus" OR "long-term SARS-nCoV-2" OR "longterm SARS-nCoV-2" OR "post acute SARS-nCoV-2" OR "postacute SARS-nCoV-2" OR "longhaul* SARS-nCoV-2" OR "long haul* SARS-nCoV-2" OR "long-haul* SARS-nCoV-2" OR "chronic* SARS-nCoV-2" OR "prolonged* SARS-nCoV-2" OR "presist* SARS-nCoV-2" OR "long-term SARS-CoV-2" OR "longterm SARS-CoV-2" OR "post acute SARS-CoV-2" OR "postacute SARS-CoV-2" OR "longhaul* SARS-CoV-2" OR "long haul* SARS-CoV-2" OR "long-haul* SARS-CoV-2" OR "chronic* SARS-CoV-2" OR "prolonged* SARS-CoV-2" OR "presist* SARS-CoV-2") **AND ab**:("clinical feature*" OR abnormal* OR characteristic* OR complication* OR condition* OR convalescence OR disorder* OR dysfunction OR illness* OR impair* OR inflammation OR manifestation OR outcome* OR prevalence OR problem* OR sequela* OR sign* OR symptom* OR syndrome))	800
10	(**ab**:("after admission" OR "after discharg*" OR "after hospital*" OR "after isolat*" OR "after quarantine" OR "after self isolat*" OR "after self-isolat*" OR "after self-quarantine" OR "post admission" OR "post discharg*" OR "post hospital*" OR "post isolat*" OR "post quarantine" OR "post self isolat*" OR "post self-isolat*" OR "post self-quarantine") **AND ab**:("clinical feature*" OR abnormal* OR characteristic* OR complication* OR condition* OR convalescence OR disorder* OR dysfunction OR illness* OR impair* OR inflammation OR manifestation OR outcome* OR prevalence OR problem* OR sequela* OR sign* OR symptom* OR syndrome))	680
11	(**ab**:(post-COVID* OR post-nCov* OR "post novel coronavirus" OR "post novel betacoronavirus" OR "post SARS-nCoV-2" OR "post SARS-CoV-2" OR postCOVID* OR "post nCov*" OR "long* COVID*" OR "long* nCov*" OR "long* novel coronavirus" OR "long* novel betacoronavirus" OR "long* SARS-nCoV-2" OR "long* SARS-CoV-2" OR "long-term COVID*" OR "long-term nCov*" OR "long-term novel coronavirus" OR "long-term novel betacoronavirus" OR "long-term SARS-nCoV-2" OR "long-term SARS-CoV-2" OR "long-term COVID*" OR "longterm COVID*" OR "post acute COVID*" OR "postacute COVID*" OR "longhaul* COVID*" OR "long haul* COVID*" OR "long-haul* COVID*" OR "chronic*	830

	COVID*" OR "prolonged* COVID*" OR "presist* COVID*" OR "long-term nCov*" OR "longterm nCov*" OR "post acute nCov*" OR "postacute nCov*" OR "longhaul* nCov*" OR "long haul* nCov*" OR "long-haul* nCov*" OR "chronic* nCov*" OR "prolonged* nCov*" OR "presist* nCov*" OR "long-term novel coronavirus" OR "longterm novel coronavirus" OR "post acute novel coronavirus" OR "postacute novel coronavirus" OR "longhaul* novel coronavirus" OR "long haul* novel coronavirus" OR "long-haul* novel coronavirus" OR "chronic* novel coronavirus" OR "prolonged* novel coronavirus" OR "presist* novel coronavirus" OR "long-term novel betacoronavirus" OR "longterm novel betacoronavirus" OR "post acute novel betacoronavirus" OR "postacute novel betacoronavirus" OR "longhaul* novel betacoronavirus" OR "long haul* novel betacoronavirus" OR "long-haul* novel betacoronavirus" OR "chronic* novel betacoronavirus" OR "prolonged* novel betacoronavirus" OR "presist* novel betacoronavirus" OR "long-term SARS-nCoV-2" OR "longterm SARS-nCoV-2" OR "post acute SARS-nCoV-2" OR "postacute SARS-nCoV-2" OR "longhaul* SARS-nCoV-2" OR "long haul* SARS-nCoV-2" OR "long-haul* SARS-nCoV-2" OR "chronic* SARS-nCoV-2" OR "prolonged* SARS-nCoV-2" OR "presist* SARS-nCoV-2" OR "long-term SARS-CoV-2" OR "longterm SARS-CoV-2" OR "post acute SARS-CoV-2" OR "postacute SARS-CoV-2" OR "longhaul* SARS-CoV-2" OR "long haul* SARS-CoV-2" OR "long-haul* SARS-CoV-2" OR "chronic* SARS-CoV-2" OR "prolonged* SARS-CoV-2" OR "presist* SARS-CoV-2") **AND tw**:("clinical feature*" OR abnormal* OR characteristic* OR complication* OR condition* OR convalescence OR disorder* OR dysfunction OR illness* OR impair* OR inflammation OR manifestation OR outcome* OR prevalence OR problem* OR sequela* OR sign* OR symptom* OR syndrome))	
12	(**ab**:("after admission" OR "after discharg*" OR "after hospital*" OR "after isolat*" OR "after quarantine" OR "after self isolat*" OR "after self-isolat*" OR "after self-quarantine" OR "post admission" OR "post discharg*" OR "post hospital*" OR "post isolat*" OR "post quarantine" OR "post self isolat*" OR "post self-isolat*" OR "post self-quarantine") **AND tw**:("clinical feature*" OR abnormal* OR characteristic* OR complication* OR condition* OR convalescence OR disorder* OR dysfunction OR illness* OR impair* OR inflammation OR manifestation OR outcome* OR prevalence OR problem* OR sequela* OR sign* OR symptom* OR syndrome))	681
13	(**tw**:(post-COVID* OR post-nCov* OR "post novel coronavirus" OR "post novel betacoronavirus" OR "post SARS-nCoV-2" OR "post SARS-CoV-2" OR postCOVID* OR "post nCov*" OR "long* COVID*" OR "long* nCov*" OR "long* novel coronavirus" OR "long* novel betacoronavirus" OR "long* SARS-nCoV-2" OR "long* SARS-CoV-2" OR "long-term COVID*" OR "long-term nCov*" OR "long-term novel coronavirus" OR "long-term novel betacoronavirus" OR "long-term SARS-nCoV-2" OR "long-term SARS-CoV-2" OR "long-term COVID*" OR "longterm COVID*" OR "post acute COVID*" OR "postacute COVID*" OR "longhaul* COVID*" OR "long haul* COVID*" OR "long-haul* COVID*" OR "chronic* COVID*" OR "prolonged* COVID*" OR "presist* COVID*" OR "long-term nCov*" OR "longterm nCov*" OR "post acute nCov*" OR "postacute nCov*" OR "longhaul* nCov*" OR "long haul* nCov*" OR "long-haul* nCov*" OR "chronic* nCov*" OR "prolonged* nCov*" OR "presist* nCov*" OR "long-term novel coronavirus" OR "longterm novel coronavirus" OR "post acute novel coronavirus" OR "postacute novel coronavirus" OR "longhaul* novel coronavirus" OR "long haul* novel coronavirus" OR "long-haul* novel coronavirus" OR "chronic* novel coronavirus" OR "prolonged* novel coronavirus" OR "presist* novel coronavirus" OR "long-term novel betacoronavirus" OR "longterm novel betacoronavirus" OR "post acute novel betacoronavirus" OR "postacute novel betacoronavirus" OR "longhaul* novel betacoronavirus" OR "long haul* novel betacoronavirus" OR "long-haul* novel betacoronavirus" OR "chronic* novel betacoronavirus" OR "prolonged* novel betacoronavirus" OR "presist* novel betacoronavirus" OR "long-term SARS-nCoV-2" OR "longterm SARS-nCoV-2" OR "post acute SARS-nCoV-2" OR "postacute SARS-nCoV-2" OR "longhaul* SARS-nCoV-2" OR "long haul* SARS-nCoV-2" OR "long-haul* SARS-nCoV-2" OR "chronic* SARS-nCoV-2" OR "prolonged* SARS-nCoV-2" OR "presist* SARS-nCoV-2" OR "long-term SARS-CoV-2" OR "longterm SARS-CoV-2" OR "post acute SARS-CoV-2" OR "postacute SARS-CoV-2" OR "longhaul* SARS-CoV-2" OR "long haul* SARS-CoV-2" OR "long-haul* SARS-CoV-2" OR "chronic* SARS-CoV-2" OR "prolonged* SARS-CoV-2" OR "presist* SARS-CoV-2") **AND** ti:("clinical feature*" OR abnormal* OR characteristic* OR complication* OR condition* OR convalescence OR disorder* OR dysfunction OR illness* OR impair* OR inflammation OR manifestation OR outcome* OR prevalence OR problem* OR sequela* OR sign* OR symptom* OR syndrome))	167
14	(**tw**:("after admission" OR "after discharg*" OR "after hospital*" OR "after isolat*" OR "after quarantine" OR "after self isolat*" OR "after self-isolat*" OR "after self-quarantine" OR "post admission" OR "post discharg*" OR "post hospital*" OR "post isolat*" OR "post quarantine" OR "post self isolat*" OR "post self-isolat*" OR "post self-quarantine") **AND** ti:("clinical feature*" OR abnormal* OR characteristic* OR complication* OR condition* OR convalescence OR disorder* OR dysfunction OR illness* OR impair* OR inflammation OR manifestation OR outcome* OR prevalence OR problem* OR sequela* OR sign* OR symptom* OR syndrome))	93
15	(**tw**:(post-COVID* OR post-nCov* OR "post novel coronavirus" OR "post novel betacoronavirus" OR "post SARS-nCoV-2" OR "post SARS-CoV-2" OR postCOVID* OR "post nCov*" OR "long* COVID*" OR "long* nCov*" OR "long* novel coronavirus" OR "long* novel betacoronavirus" OR "long* SARS-nCoV-2" OR "long* SARS-CoV-2" OR "long-term COVID*" OR "long-term nCov*" OR "long-term novel coronavirus" OR "long-term novel betacoronavirus" OR "long-term SARS-nCoV-2" OR "long-term SARS-CoV-2" OR "long-term COVID*" OR "longterm COVID*" OR "post acute COVID*" OR "postacute COVID*" OR "longhaul* COVID*" OR "long haul* COVID*" OR "long-haul* COVID*" OR "chronic* COVID*" OR "prolonged* COVID*" OR "presist* COVID*" OR "long-term nCov*" OR "longterm nCov*" OR "post acute nCov*" OR "postacute nCov*" OR "longhaul* nCov*" OR "long haul* nCov*" OR "long-haul* nCov*" OR "chronic* nCov*" OR "prolonged* nCov*" OR "presist* nCov*" OR "long-term novel coronavirus" OR "longterm novel	752

	coronavirus" OR "post acute novel coronavirus" OR "postacute novel coronavirus" OR "longhaul* novel coronavirus" OR "long haul* novel coronavirus" OR "long-haul* novel coronavirus" OR "chronic* novel coronavirus" OR "prolonged* novel coronavirus" OR "presist* novel coronavirus" OR "long-term novel betacoronavirus" OR "longterm novel betacoronavirus" OR "post acute novel betacoronavirus" OR "postacute novel betacoronavirus" OR "longhaul* novel betacoronavirus" OR "long haul* novel betacoronavirus" OR "long-haul* novel betacoronavirus" OR "chronic* novel betacoronavirus" OR "prolonged* novel betacoronavirus" OR "presist* novel betacoronavirus" OR "long-term SARS-nCoV-2" OR "longterm SARS-nCoV-2" OR "post acute SARS-nCoV-2" OR "postacute SARS-nCoV-2" OR "longhaul* SARS-nCoV-2" OR "long haul* SARS-nCoV-2" OR "long-haul* SARS-nCoV-2" OR "chronic* SARS-nCoV-2" OR "prolonged* SARS-nCoV-2" OR "presist* SARS-nCoV-2" OR "long-term SARS-CoV-2" OR "longterm SARS-CoV-2" OR "post acute SARS-CoV-2" OR "postacute SARS-CoV-2" OR "longhaul* SARS-CoV-2" OR "long haul* SARS-CoV-2" OR "long-haul* SARS-CoV-2" OR "chronic* SARS-CoV-2" OR "prolonged* SARS-CoV-2" OR "presist* SARS-CoV-2") **AND ab**:("clinical feature*" OR abnormal* OR characteristic* OR complication* OR condition* OR convalescence OR disorder* OR dysfunction OR illness* OR impair* OR inflammation OR manifestation OR outcome* OR prevalence OR problem* OR sequela* OR sign* OR symptom* OR syndrome))	
16	(**tw**:("after admission" OR "after discharg*" OR "after hospital*" OR "after isolat*" OR "after quarantine" OR "after self isolat*" OR "after self-isolat*" OR "after self-quarantine" OR "post admission" OR "post discharg*" OR "post hospital*" OR "post isolat*" OR "post quarantine" OR "post self isolat*" OR "post self-isolat*" OR "post self-quarantine") **AND ab**:("clinical feature*" OR abnormal* OR characteristic* OR complication* OR condition* OR convalescence OR disorder* OR dysfunction OR illness* OR impair* OR inflammation OR manifestation OR outcome* OR prevalence OR problem* OR sequela* OR sign* OR symptom* OR syndrome))	339
17	(**tw**:(post-COVID* OR post-nCov* OR "post novel coronavirus" OR "post novel betacoronavirus" OR "post SARS-nCoV-2" OR "post SARS-CoV-2" OR postCOVID* OR "post nCov*" OR "long* COVID*" OR "long* nCov*" OR "long* novel coronavirus" OR "long* novel betacoronavirus" OR "long* SARS-nCoV-2" OR "long* SARS-CoV-2" OR "long-term COVID*" OR "long-term nCov*" OR "long-term novel coronavirus" OR "long-term novel betacoronavirus" OR "long-term SARS-nCoV-2" OR "long-term SARS-CoV-2" OR "long-term COVID*" OR "longterm COVID*" OR "post acute COVID*" OR "postacute COVID*" OR "longhaul* COVID*" OR "long haul* COVID*" OR "long-haul* COVID*" OR "chronic* COVID*" OR "prolonged* COVID*" OR "presist* COVID*" OR "long-term nCov*" OR "longterm nCov*" OR "post acute nCov*" OR "postacute nCov*" OR "longhaul* nCov*" OR "long haul* nCov*" OR "long-haul* nCov*" OR "chronic* nCov*" OR "prolonged* nCov*" OR "presist* nCov*" OR "long-term novel coronavirus" OR "longterm novel coronavirus" OR "post acute novel coronavirus" OR "postacute novel coronavirus" OR "longhaul* novel coronavirus" OR "long haul* novel coronavirus" OR "long-haul* novel coronavirus" OR "chronic* novel coronavirus" OR "prolonged* novel coronavirus" OR "presist* novel coronavirus" OR "long-term novel betacoronavirus" OR "longterm novel betacoronavirus" OR "post acute novel betacoronavirus" OR "postacute novel betacoronavirus" OR "longhaul* novel betacoronavirus" OR "long haul* novel betacoronavirus" OR "long-haul* novel betacoronavirus" OR "chronic* novel betacoronavirus" OR "prolonged* novel betacoronavirus" OR "presist* novel betacoronavirus" OR "long-term SARS-nCoV-2" OR "longterm SARS-nCoV-2" OR "post acute SARS-nCoV-2" OR "postacute SARS-nCoV-2" OR "longhaul* SARS-nCoV-2" OR "long haul* SARS-nCoV-2" OR "long-haul* SARS-nCoV-2" OR "chronic* SARS-nCoV-2" OR "prolonged* SARS-nCoV-2" OR "presist* SARS-nCoV-2" OR "long-term SARS-CoV-2" OR "longterm SARS-CoV-2" OR "post acute SARS-CoV-2" OR "postacute SARS-CoV-2" OR "longhaul* SARS-CoV-2" OR "long haul* SARS-CoV-2" OR "long-haul* SARS-CoV-2" OR "chronic* SARS-CoV-2" OR "prolonged* SARS-CoV-2" OR "presist* SARS-CoV-2") **AND tw**:("clinical feature*" OR abnormal* OR characteristic* OR complication* OR condition* OR convalescence OR disorder* OR dysfunction OR illness* OR impair* OR inflammation OR manifestation OR outcome* OR prevalence OR problem* OR sequela* OR sign* OR symptom* OR syndrome))	906
18	(**tw**:("after admission" OR "after discharg*" OR "after hospital*" OR "after isolat*" OR "after quarantine" OR "after self isolat*" OR "after self-isolat*" OR "after self-quarantine" OR "post admission" OR "post discharg*" OR "post hospital*" OR "post isolat*" OR "post quarantine" OR "post self isolat*" OR "post self-isolat*" OR "post self-quarantine") **AND tw**:("clinical feature*" OR abnormal* OR characteristic* OR complication* OR condition* OR convalescence OR disorder* OR dysfunction OR illness* OR impair* OR inflammation OR manifestation OR outcome* OR prevalence OR problem* OR sequela* OR sign* OR symptom* OR syndrome))	340

Michelen M, *et al. BMJ Global Health* 2021; 6:e005427. doi: 10.1136/bmjgh-2021-005427

BMJ Publishing Group Limited (BMJ) disclaims all liability and responsibility arising from any reliance placed on this supplemental material which has been supplied by the author(s)

Supplement 2: Risk of bias assessment

Study	Representation of national population (e.g. age, sex, occupation)	Sampling frame true or close representation of target population	Random selection used to select sample, OR, census undertaken	Likelihood of non-response bias minimal	Data collected directly from subjects (opposed to proxy)	Acceptable case definition used	Instrument to measure parameter of interest has reliability and validity (if necessary)	Same mode of data collection used for all subjects	Length of shortest prevalence period for parameter of interest appropriate	Numerator(s)/ denominator(s) for parameter of interest appropriate	Overall risk of bias
Alharthy et al.	High	High	High	Low	Low	Low	Low	Low	Low	Low	Moderate
Anastasio et al.	Low	Low	Low	Low	Low	Low	Low	Low	Low	Low	Low
Arnold et al.	High	Low	High	Low	Low	Low	Low	Low	Low	Low	Moderate
Baricich et al.	High	High	High	Low	Low	Low	Low	Low	Low	High	High
Bellan et al.	High	Low	High	Low	Low	Low	Low	Low	Low	Low	Moderate
Blanco et al.	High	High	High	Low	Low	Low	Low	Low	Low	Low	Moderate
Doyle et al.	High	High	High	Low	Low	Low	Low	Low	Low	Low	Moderate
Einvik et al.	High	Low	High	Low	Low	Low	Low	Low	Low	Low	Moderate
Garrigues et al.	High	High	High	High	Low	Low	Low	Low	Low	Low	High
Gherlone et al.	High	High	High	Low	Low	Low	Low	Low	Low	High	High
Han et al.	High	Low	High	Low	Low	Low	Low	Low	Low	Low	Moderate
Hopkins et al.	High	High	High	Low	Low	Low	High	Low	Low	Low	High
Huang et al.	Low	Low	Low	High	Low	Low	Low	Low	Low	Low	Low
Jacobson et al.	High	Low	High	Low	Low	Low	Low	Low	Low	Low	Moderate
Klein et al.	High	High	High	Low	Low	Low	High	Low	Low	Low	High
Lerum et al.	High	Low	High	Low	Low	Low	Low	Low	High	Low	Moderate
Logue et al.	High	Low	High	Low	Low	Low	Low	Low	Low	Low	Moderate
Mazza et al.	High	High	High	Low	Low	Low	Low	Low	Low	Low	Moderate
Mendez et al.	High	High	High	Low	Low	Low	Low	Low	High	Low	High
Nguyen et al.	High	Low	Low	Low	Low	Low	High	Low	Low	Low	Moderate
Nugent et al.	High	Low	High	Low	Low	Low	Low	Low	Low	Low	Moderate
Parente-Arias et al.	High	High	High	Low	Low	Low	Low	Low	Low	Low	Moderate
Petersen et al.	Low	Low	High	High	Low	Low	High	Low	High	Low	High
Qin et al.	High	High	High	High	Low	Low	Low	Low	Low	Low	High
Qu et al.	Low	Low	High	Low	Low	Low	Low	Low	Low	Low	Low
Rass et al.	High	High	High	Low	Low	Low	Low	Low	Low	Low	Moderate
Sibila et al.	High	High	High	Low	Low	Low	Low	Low	Low	High	High
Simani et al.	Low	Low	Low	High	Low	Low	Low	Low	Low	Low	Low
Sonnweber et al.	High	Low	High	Low	Low	Low	Low	Low	Low	Low	Moderate
Stavem et al.	Low	High	High	Low	Low	Low	High	Low	High	Low	High
Suarez-Robles et al.	High	High	High	Low	Low	Low	Low	Low	Low	Low	Moderate
Sykes et al.	High	Low	Low	High	Low	Low	Low	Low	Low	Low	Moderate
Taboada et al.	High	High	High	Low	Low	Low	Low	Low	Low	Low	Moderate
Venturelli et al.	Low	High	High	High	Low	Low	Low	Low	Low	Low	Moderate
Weng et al.	High	High	High	High	Low	Low	Low	Low	Low	High	High
Xiong et al.	Low	High	High	Low	Low	Low	Low	Low	Low	Low	Moderate
Xu et al.	High	High	Low	Low	Low	Low	Low	Low	Low	Low	Moderate
Zhang et al. (a)	High	High	High	High	Low	Low	Low	Low	Low	High	High
Zhang et al. (b)	High	Low	Low	Low	Low	Low	Low	Low	Low	Low	Low

Low Risk | Moderate risk | High risk

Michelen M, *et al. BMJ Global Health* 2021; 6:e005427. doi: 10.1136/bmjgh-2021-005427

Supplement 3: PRISMA diagram

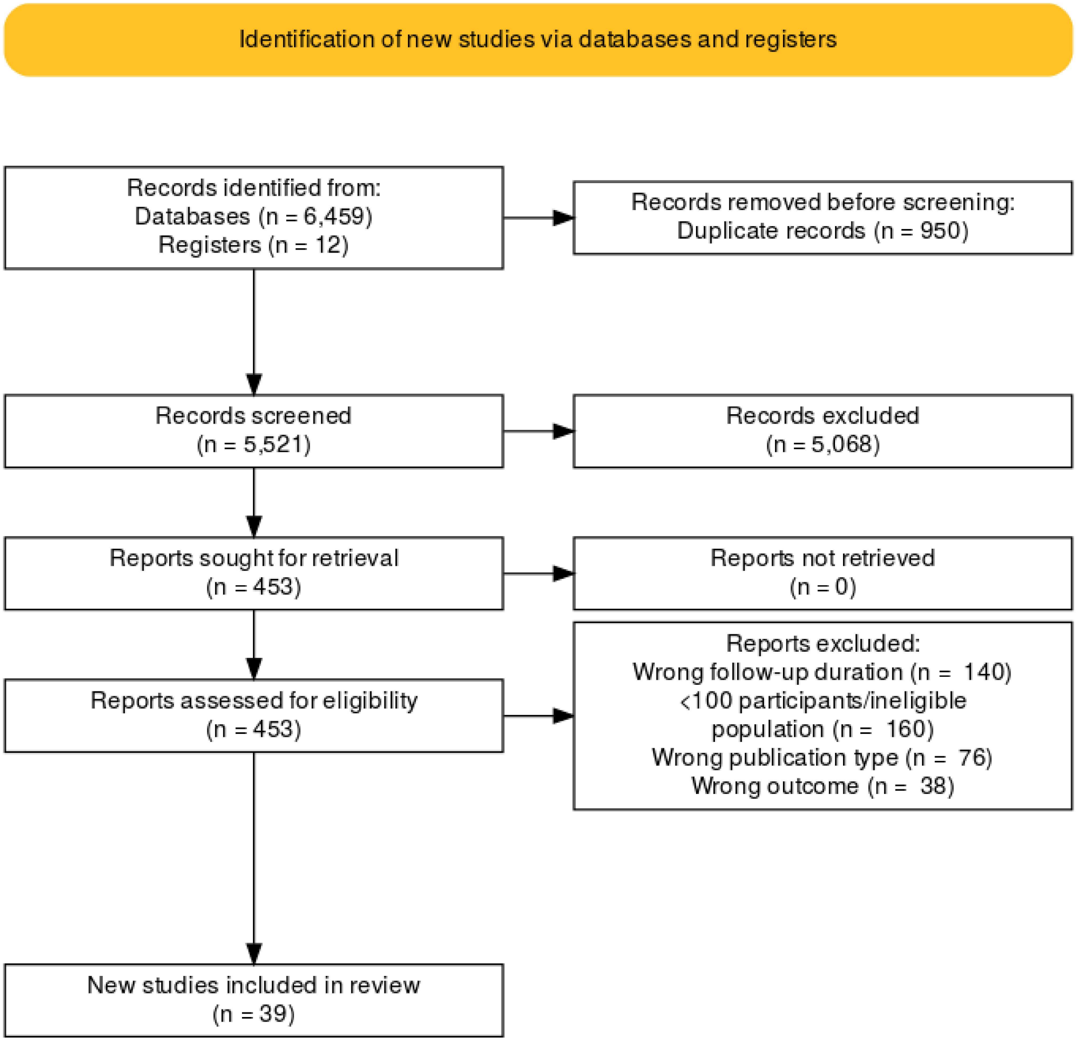

Supplement 4: Individual forest plots in main results

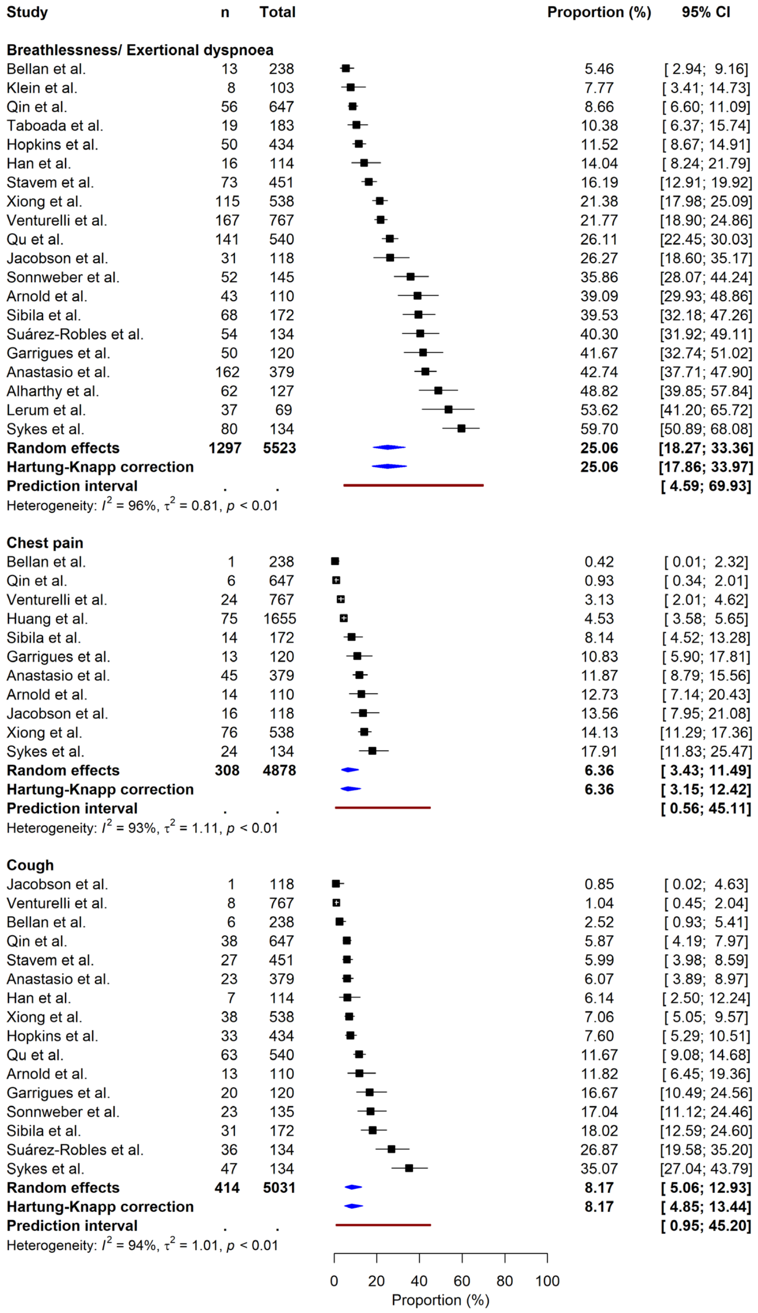

Figure 1. Cardiopulmonary

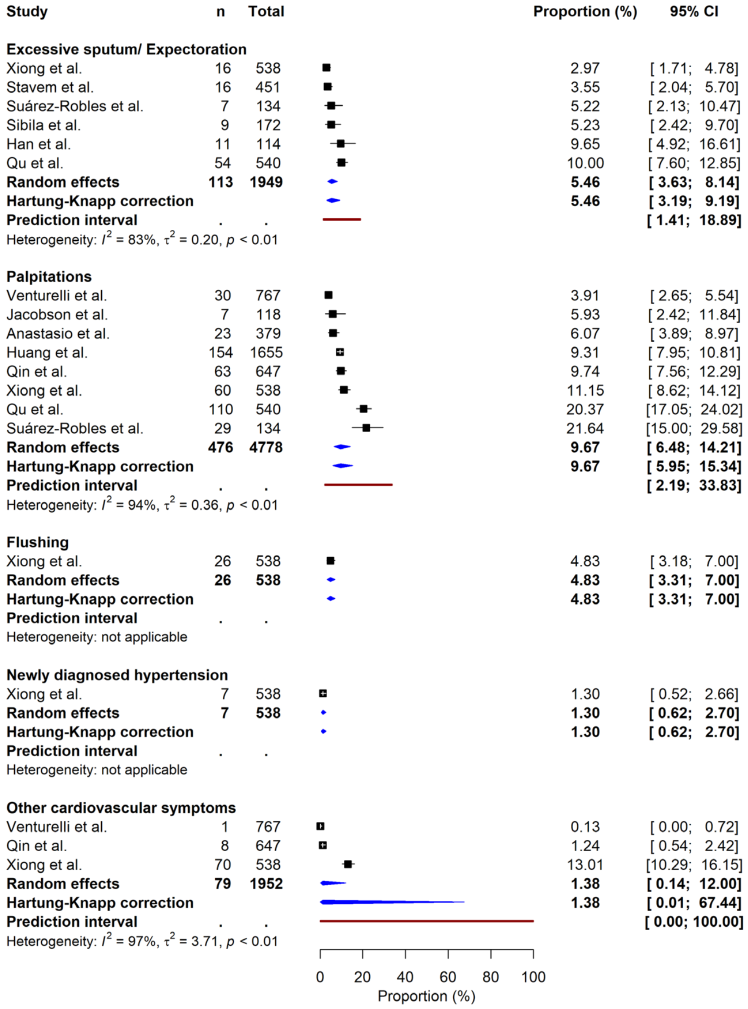

Figure 2.Cardiopulmonary (page 2)

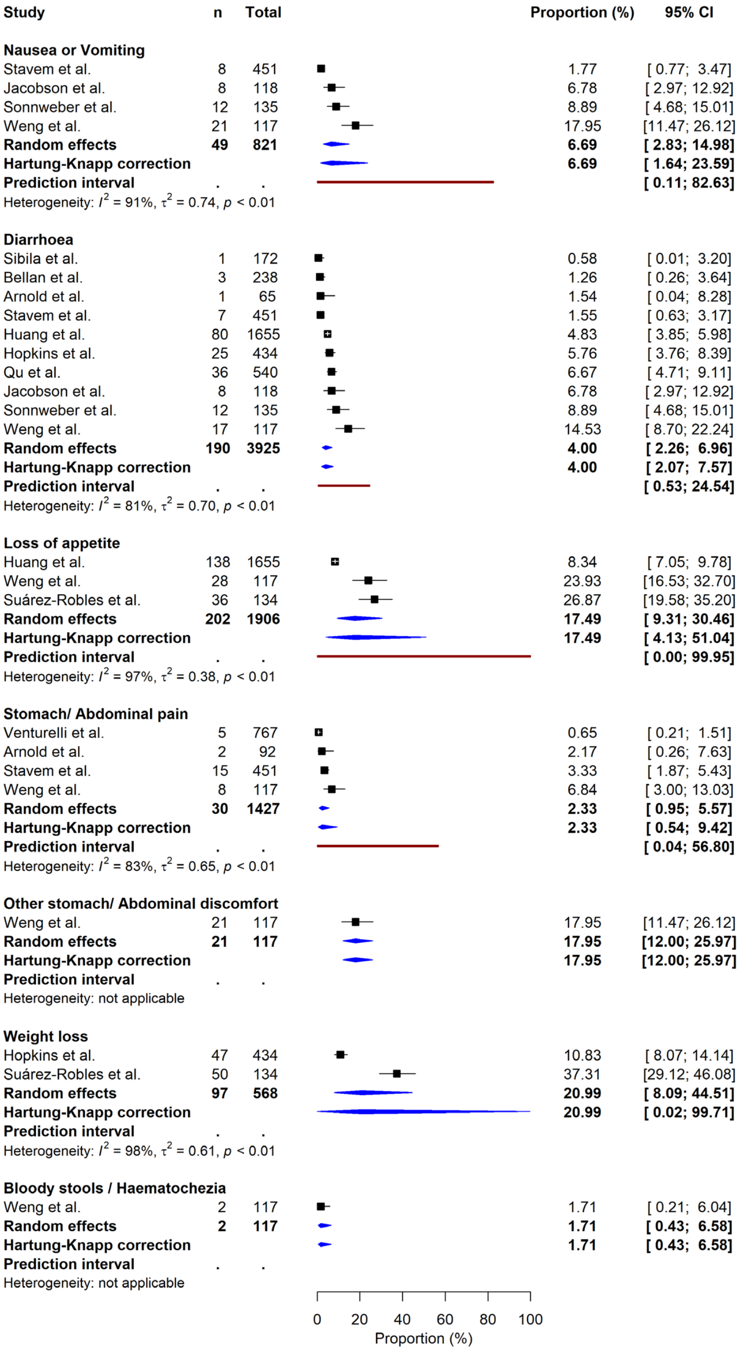

Figure 3. Gastrointestinal

Michelen M, *et al. BMJ Global Health* 2021; 6:e005427. doi: 10.1136/bmjgh-2021-005427

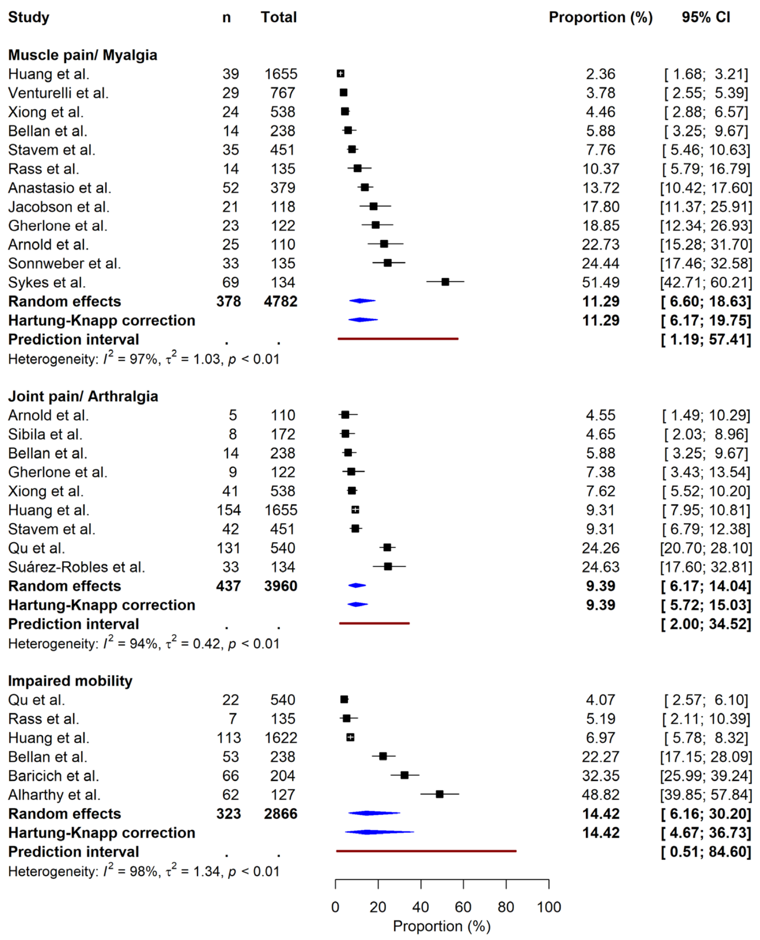

Figure 4. Musculoskeletal

Michelen M, *et al. BMJ Global Health* 2021; 6:e005427. doi: 10.1136/bmjgh-2021-005427

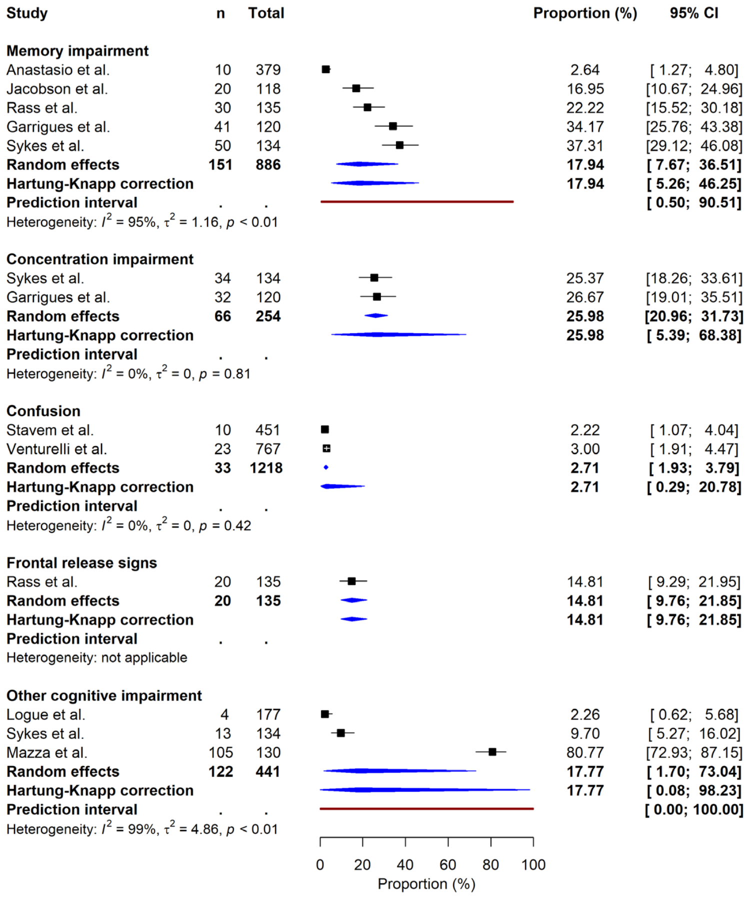

Figure 5. Neurocognitive

Michelen M, *et al. BMJ Global Health* 2021; 6:e005427. doi: 10.1136/bmjgh-2021-005427

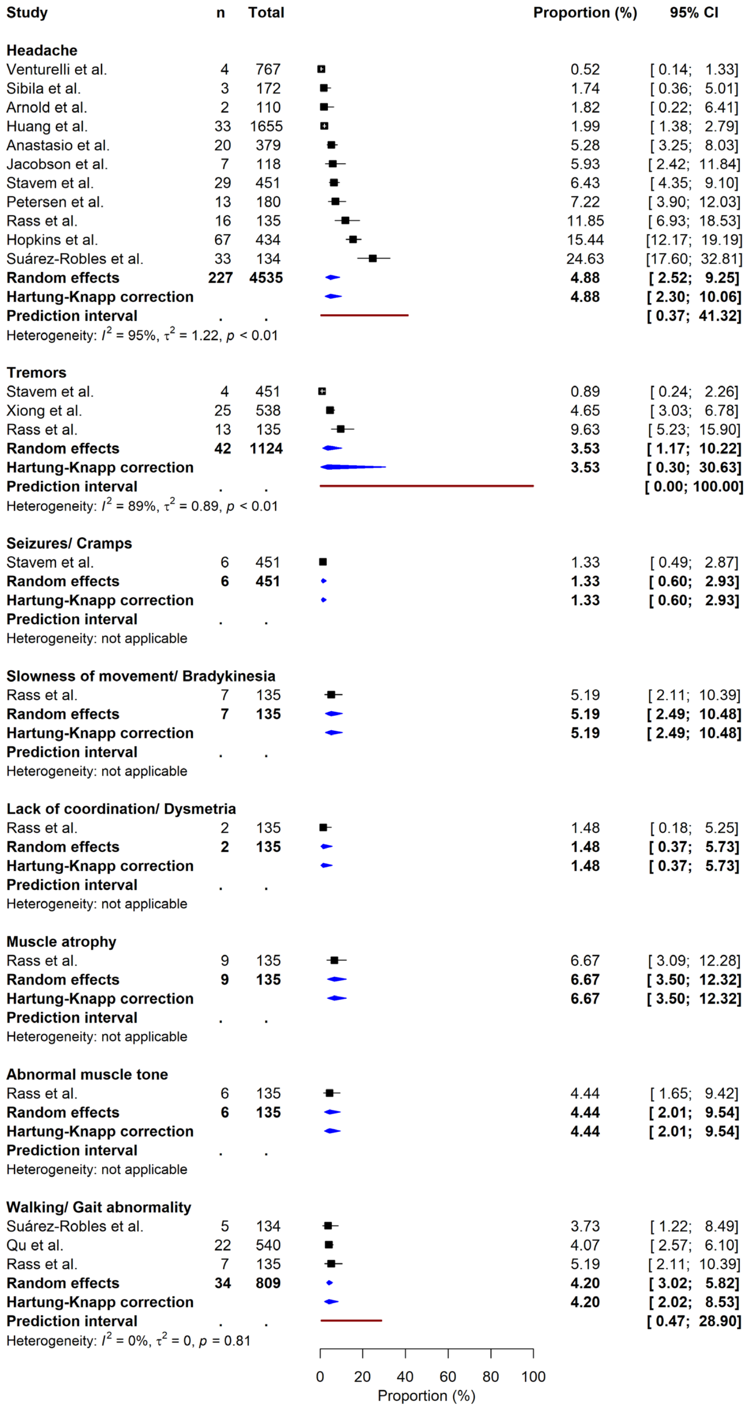

Figure 6. Neurological and neuromuscular

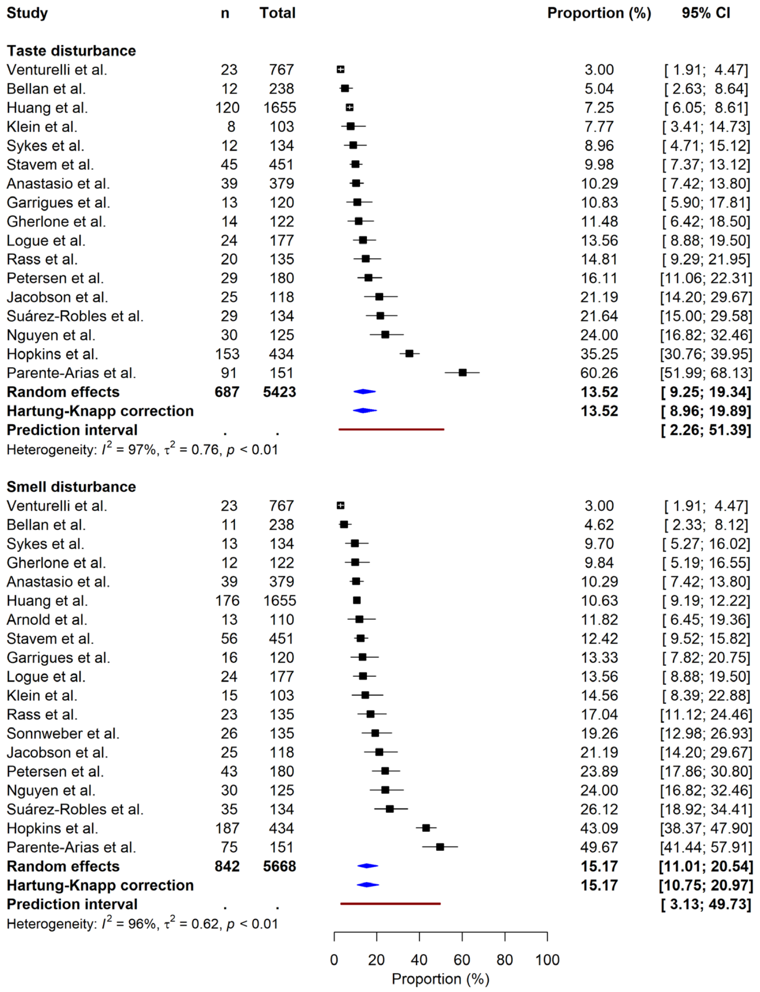

Figure 7.Neurological and neuromuscular (page 2)

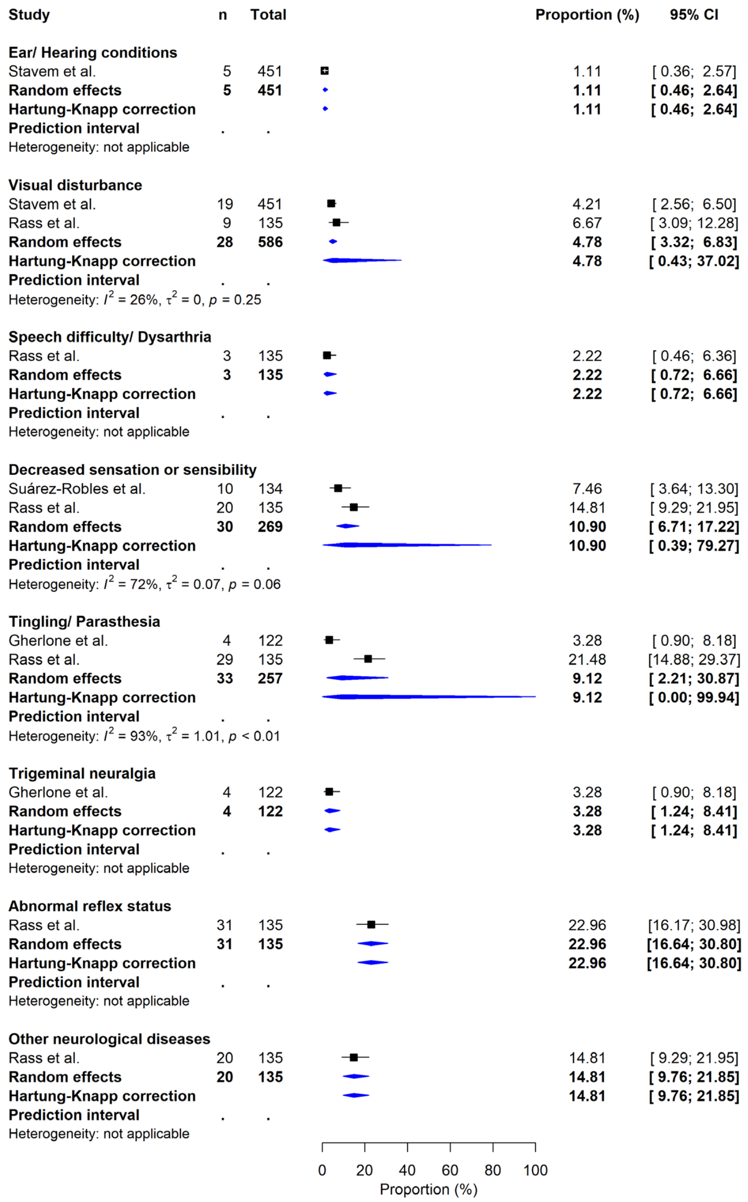

Figure 8. Neurological and neuromuscular (page 3)

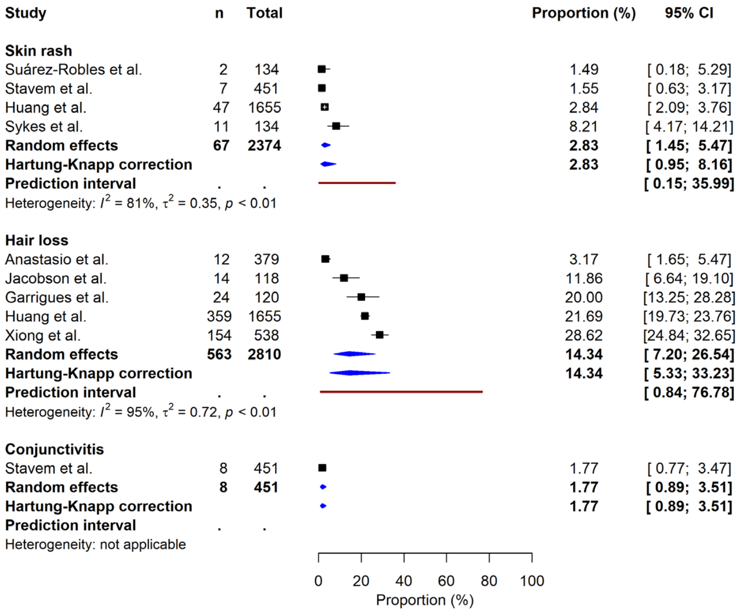

Figure 9. Other

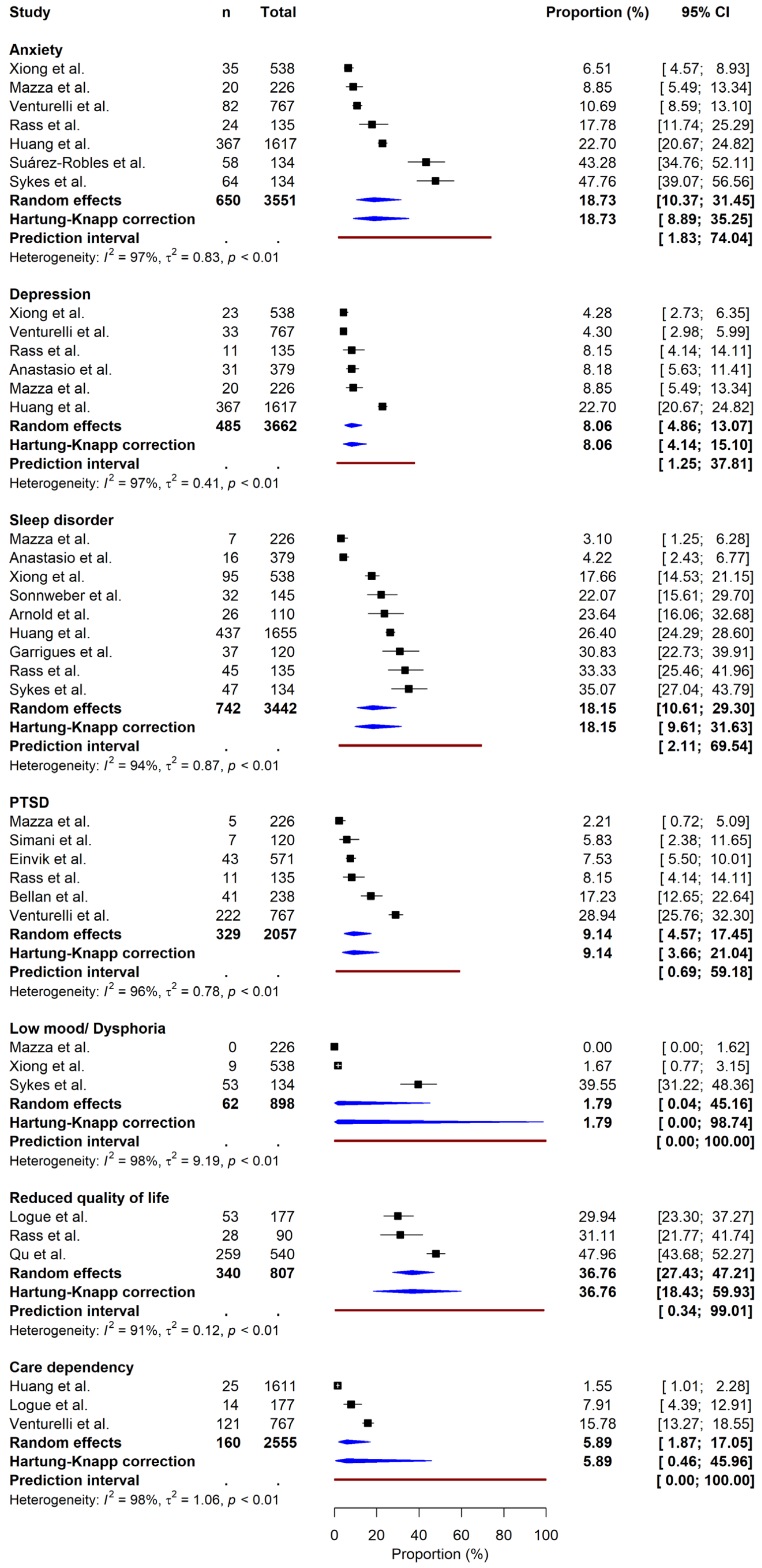

Figure 10. Psychological and social

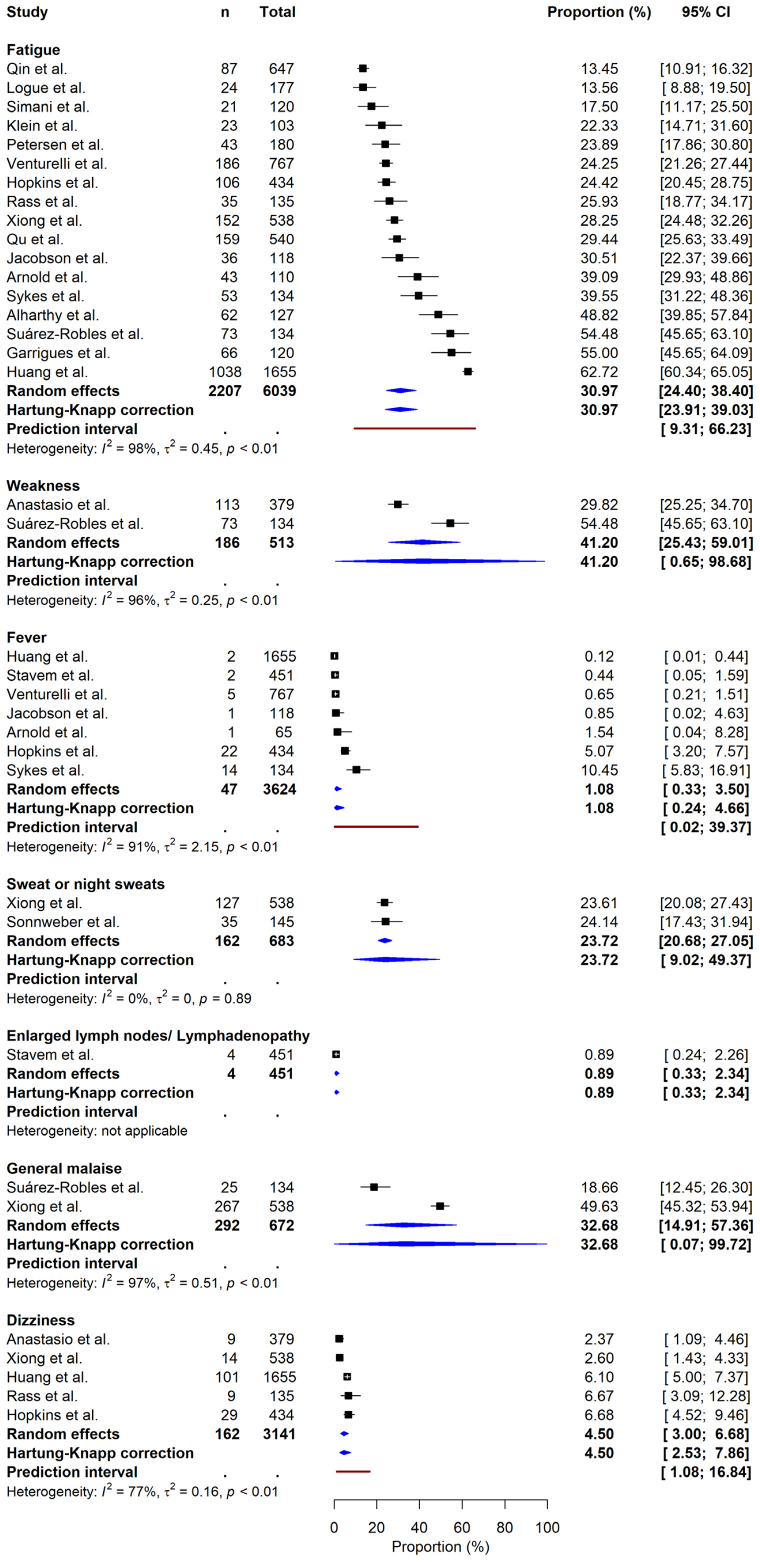

Figure 11. Systemic

Michelen M, *et al. BMJ Global Health* 2021; 6:e005427. doi: 10.1136/bmjgh-2021-005427

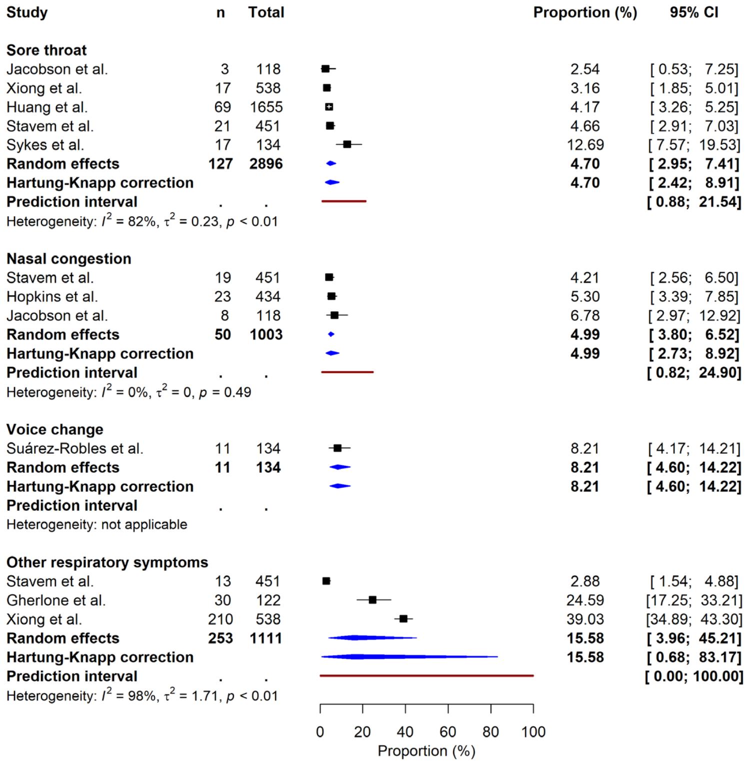

Figure 12. Upper respiratory

Supplement 5: Subgroup analysis: hospitalisation

Classification	Symptom	Subgroup	N Studies	Event/total	Proportion% (95% CIs)	Heterogeneity (%)	P value
Cardiopulmonary	Breathlessness/ Exertional dyspnoea	Hospitalised	n = 14	765/3148	28.68 (18.48 to 41.64)	96.19	<0.001
		Mixed	n = 3	381/1291	32.57 (14.26 to 58.38)	96.38	
		Non-hospitalised	n = 4	151/1084	13.72 (8.51 to 21.37)	72.13	
	Chest pain	Mixed	n = 2	69/1146	6.18 (0.01 to 97.66)	96.65	0.071
		Hospitalised	n = 9	225/3636	5.92 (2.45 to 13.63)	92.86	
		Non-hospitalised	n = 1	14/ 96	14.58 (8.83 to 23.13)	NA	
	Cough	Mixed	n = 3	54/1281	4.91 (0.25 to 51.82)	96.03	0.265
		Hospitalised	n = 11	299/2769	10.52 (5.93 to 17.98)	93.05	
		Non-hospitalised	n = 3	61/981	5.95 (1.53 to 20.50)	56.24	
	Excessive sputum/ Expectoration	Hospitalised	n = 5	97/1498	6.02 (3.20 to 11.03)	82.16	0.112
		Non-hospitalised	n = 1	16/451	3.55 (2.18 to 5.71)	NA	
	Other cardiovascular symptoms	Hospitalised	n = 2	78/1185	4.20 (0.00 to 99.97)	97.68	0.009
		Mixed	n = 1	1/767	0.13 (0.02 to 0.92)	NA	
	Palpitations	Mixed	n = 2	53/1146	4.67 (0.60 to 28.47)	62.05	<0.001
		Hospitalised	n = 6	416/3536	12.43 (7.78 to 19.29)	91.7	
		Non-hospitalised	n = 1	7/ 96	7.29 (3.52 to 14.51)	NA	
Gastrointestinal	Diarrhoea	Hospitalised	n = 7	138/2809	2.93 (0.90 to 9.12)	81.91	0.077
		Non-hospitalised	n = 3	40/981	4.16 (0.72 to 20.65)	84.27	
		Mixed	n = 1	12/135	8.89 (5.12 to 15.00)	NA	
	Nausea or Vomiting	Hospitalised	n = 2	21/139	5.84 (0.00 to 100.00)	0	0.343
		Non-hospitalised	n = 2	16/547	3.66 (0.00 to 98.24)	89.91	
		Mixed	n = 1	12/135	8.89 (5.12 to 15.00)	NA	
	Stomach/ Abdominal pain	Hospitalised	n = 2	10/209	4.63 (0.03 to 89.20)	54.79	0.002
		Non-hospitalised	n = 1	15/451	3.33 (2.01 to 5.44)	NA	
		Mixed	n = 1	5/767	0.65 (0.27 to 1.56)	NA	
	Weight loss	Non-hospitalised	n = 1	47/434	10.83 (8.23 to 14.12)	NA	<0.001
		Hospitalised	n = 1	50/134	37.31 (29.55 to 45.79)	NA	
Musculoskeletal	Impaired mobility	Hospitalised	n = 5	316/2731	17.33 (4.75 to 46.83)	98.49	0.038
		Mixed	n = 1	7/135	5.19 (2.49 to 10.48)	NA	

	Joint pain/ Arthralgia	Hospitalised	n = 8	395/3509	9.36 (5.25 to 16.14)	94.81	0.987
		Non-hospitalised	n = 1	42/451	9.31 (6.95 to 12.36)	NA	
	Muscle pain/ Myalgia	Mixed	n = 4	128/1416	10.86 (3.45 to 29.36)	95.21	0.954
		Hospitalised	n = 7	199/2819	12.46 (4.30 to 31.09)	98.05	
		Non-hospitalised	n = 2	51/547	10.76 (0.24 to 85.64)	85.87	
Neurocognitive	Confusion	Non-hospitalised	n = 1	10/451	2.22 (1.20 to 4.07)	NA	0.419
		Mixed	n = 1	23/767	3.00 (2.00 to 4.47)	NA	
	Memory impairment	Mixed	n = 2	40/514	8.06 (0.00 to 99.97)	97.38	<0.001
		Hospitalised	n = 3	96/276	34.78 (23.64 to 47.88)	0	
		Non-hospitalised	n = 1	15/ 96	15.62 (9.64 to 24.32)	NA	
	Other cognitive impairment	Mixed	n = 2	109/307	23.55 (0.00 to 100.00)	98.87	0.581
		Hospitalised	n = 1	13/134	9.70 (5.72 to 15.99)	NA	
Neurological and neuromuscular	Decreased sensation or sensibility	Mixed	n = 1	20/135	14.81 (9.76 to 21.85)	NA	0.060
		Hospitalised	n = 1	10/134	7.46 (4.06 to 13.31)	NA	
	Headache	Mixed	n = 3	40/1281	3.30 (0.12 to 50.20)	93.93	0.145
		Hospitalised	n = 5	71/2093	2.98 (0.47 to 16.53)	96.56	
		Non-hospitalised	n = 4	116/1161	8.82 (4.41 to 16.85)	86.25	
	Smell disturbance	Mixed	n = 6	210/1744	14.63 (5.46 to 33.72)	97.32	0.108
		Hospitalised	n = 9	308/2660	12.16 (7.98 to 18.10)	85.48	
		Non-hospitalised	n = 5	324/1264	22.19 (11.69 to 38.04)	96.3	
	Taste disturbance	Mixed	n = 5	197/1609	14.50 (3.40 to 44.98)	98.32	0.425
		Hospitalised	n = 8	232/2550	11.07 (6.90 to 17.28)	89.1	
		Non-hospitalised	n = 5	258/1264	16.83 (7.91 to 32.26)	95.66	
	Tingling/ Paraesthesia	Hospitalised	n = 1	4/122	3.28 (1.24 to 8.41)	NA	<0.001
		Mixed	n = 1	29/135	21.48 (15.36 to 29.21)	NA	
	Tremors	Mixed	n = 1	13/135	9.63 (5.67 to 15.88)	NA	<0.001
		Non-hospitalised	n = 1	4/451	0.89 (0.33 to 2.34)	NA	
		Hospitalised	n = 1	25/538	4.65 (3.16 to 6.79)	NA	
	Visual disturbance	Mixed	n = 1	9/135	6.67 (3.50 to 12.32)	NA	0.245
		Non-hospitalised	n = 1	19/451	4.21 (2.70 to 6.51)	NA	
	Walking/ Gait abnormality	Hospitalised	n = 2	27/674	4.01 (0.34 to 33.61)	0	0.534
		Mixed	n = 1	7/135	5.19 (2.49 to 10.48)	NA	

Other	Hair loss	Mixed	n = 1	12/379	3.17 (1.81 to 5.49)	NA	<0.001
		Hospitalised	n = 4	541/2335	23.54 (17.68 to 30.61)	74.84	
		Non-hospitalised	n = 1	10/ 96	10.42 (5.70 to 18.29)	NA	
	Skin rash	Hospitalised	n = 3	60/1923	3.53 (0.75 to 15.11)	82.97	0.112
		Non-hospitalised	n = 1	7/451	1.55 (0.74 to 3.22)	NA	
Psychological and social	Anxiety	Hospitalised	n = 4	524/2423	25.58 (6.36 to 63.49)	97.85	0.072
		Mixed	n = 3	126/1128	11.60 (6.03 to 21.15)	72	
	Care dependency	Hospitalised	n = 1	25/1611	1.55 (1.05 to 2.29)	NA	<0.001
		Mixed	n = 2	135/944	12.00 (0.39 to 82.45)	85.63	
	Depression	Mixed	n = 4	95/1507	6.80 (3.99 to 11.37)	71	0.506
		Hospitalised	n = 2	390/2155	10.38 (0.00 to 99.83)	98.62	
	Low mood/ Dysphoria	Mixed	n = 1	0/226	0.00 (0.00 to 100.00)	NA	1.000
		Hospitalised	n = 2	62/672	9.49 (0.00 to 100.00)	98.92	
	PTSD	Hospitalised	n = 3	59/474	10.52 (3.06 to 30.44)	80.04	0.458
		Non-hospitalised	n = 1	32/455	7.03 (5.02 to 9.78)	NA	
		Mixed	n = 3	238/1128	8.73 (0.46 to 66.23)	96.63	
	Reduced quality of life	Mixed	n = 2	81/267	30.34 (7.43 to 70.27)	0	<0.001
		Hospitalised	n = 1	259/540	47.96 (43.77 to 52.18)	NA	
	Sleep disorder	Mixed	n = 4	100/885	10.66 (1.76 to 44.22)	96.51	0.081
		Hospitalised	n = 5	642/2557	25.81 (18.85 to 34.26)	84.7	
Systemic	Dizziness	Mixed	n = 2	18/514	3.78 (0.03 to 83.74)	79.93	0.224
		Non-hospitalised	n = 1	29/434	6.68 (4.68 to 9.45)	NA	
		Hospitalised	n = 2	115/2193	4.21 (0.08 to 71.53)	89.39	
	Fatigue	Hospitalised	n = 11	1762/4147	37.10 (26.54 to 49.06)	98.23	0.017
		Non-hospitalised	n = 4	200/813	24.60 (20.11 to 29.72)	0	
		Mixed	n = 3	245/1079	21.04 (10.48 to 37.75)	79.86	
	Fever	Hospitalised	n = 4	17/1876	0.85 (0.02 to 24.20)	92.05	0.661
		Non-hospitalised	n = 3	25/981	1.41 (0.06 to 24.82)	84.73	
		Mixed	n = 1	5/767	0.65 (0.27 to 1.56)	NA	
	Sweat or night sweats	Mixed	n = 1	35/145	24.14 (17.87 to 31.76)	NA	0.894
		Hospitalised	n = 1	127/538	23.61 (20.21 to 27.38)	NA	
	Weakness	Mixed	n = 1	113/379	29.82 (25.42 to 34.61)	NA	<0.001

		Hospitalised	n = 1	73/134	54.48 (46.00 to 62.70)	NA	
Upper respiratory	Nasal congestion	Non-hospitalised	n = 3	49/981	4.99 (2.72 to 8.99)	0	0.924
		Hospitalised	n = 1	1/ 22	4.55 (0.64 to 26.15)	NA	
	Other respiratory symptoms	Hospitalised	n = 2	240/660	32.43 (2.22 to 91.02)	88.57	<0.001
		Non-hospitalised	n = 1	13/451	2.88 (1.68 to 4.90)	NA	
	Sore throat	Hospitalised	n = 4	103/2349	4.81 (1.60 to 13.60)	85.83	0.815
		Non-hospitalised	n = 2	24/547	4.39 (0.32 to 39.44)	0	

Supplement 6: Subgroup analysis: Setting

Classification	Symptom	Subgroup	N Studies	Event/total	Proportion% (95% CIs)	Heterogeneity (%)	P value
Cardiopulmonary	Cough	Multicentre	n = 5	121/1358	6.90 (2.46 to 17.92)	84.05	0.842
		Single-centre	n = 10	260/3239	9.07 (4.23 to 18.41)	95.62	
		Online survey	n = 1	33/ 434	7.60 (5.46 to 10.50)	NA	
	Chest pain	Single-centre	n = 10	292/4760	5.87 (2.70 to 12.26)	93.7	0.039
		Multicentre	n = 1	16/ 118	13.56 (8.48 to 20.99)	NA	
	Breathlessness/ Exertional dyspnoea	Multicentre	n = 6	350/1437	26.79 (15.81 to 41.63)	91.82	<0.001
		Single-centre	n = 12	889/3549	27.78 (17.16 to 41.67)	96.93	
		Online survey	n = 2	58/ 537	10.80 (2.03 to 41.47)	16.75	
	Palpitations	Single-centre	n = 6	359/4120	9.02 (5.17 to 15.27)	90.72	0.571
		Multicentre	n = 2	117/ 658	11.96 (0.02 to 98.84)	91.67	
	Excessive sputum/ Expectoration	Multicentre	n = 3	81/1105	6.97 (2.02 to 21.38)	86.45	0.066
		Single-centre	n = 3	32/ 844	3.79 (1.78 to 7.88)	24.53	
Gastrointestinal	Weight loss	Online survey	n = 1	47/ 434	10.83 (8.23 to 14.12)	NA	<0.001
		Single-centre	n = 1	50/ 134	37.31 (29.55 to 45.79)	NA	
	Stomach/ Abdominal pain	Single-centre	n = 2	7/ 859	0.81 (0.01 to 50.51)	52.12	<0.001
		Multicentre	n = 2	23/ 568	4.05 (0.28 to 38.69)	64.62	
	Loss of appetite	Single-centre	n = 2	174/1789	15.09 (0.03 to 98.93)	97.64	0.288
		Multicentre	n = 1	28/ 117	23.93 (17.06 to 32.48)	NA	
	Diarrhoea	Single-centre	n = 4	85/2130	1.81 (0.36 to 8.72)	72.7	0.081
		Multicentre	n = 5	80/1361	6.23 (2.49 to 14.76)	85.33	
		Online survey	n = 1	25/ 434	5.76 (3.92 to 8.39)	NA	
Musculoskeletal	Impaired mobility	Single-centre	n = 4	294/2191	23.71 (6.53 to 58.01)	98.62	<0.001
		Multicentre	n = 2	29/ 675	4.30 (0.40 to 33.37)	0	
	Joint pain/ Arthralgia	Single-centre	n = 7	264/2969	8.03 (4.64 to 13.55)	86.94	0.116
		Multicentre	n = 2	173/ 991	15.45 (0.11 to 96.89)	97.19	
	Muscle pain/ Myalgia	Multicentre	n = 4	103/ 839	13.72 (6.26 to 27.48)	89.49	0.508
		Single-centre	n = 8	275/3943	10.23 (4.03 to 23.63)	97.97	
Neurocognitive	Other cognitive impairment	Single-centre	n = 2	118/ 264	40.19 (0.00 to 100.00)	99	0.017
		Multicentre	n = 1	4/ 177	2.26 (0.85 to 5.86)	NA	

	Confusion	Multicentre	n = 1	10/ 451	2.22 (1.20 to 4.07)	NA	0.419
		Single-centre	n = 1	23/ 767	3.00 (2.00 to 4.47)	NA	
	Memory impairment	Multicentre	n = 2	50/ 253	19.76 (3.21 to 64.68)	8.97	0.821
		Single-centre	n = 3	101/ 633	16.93 (0.58 to 87.62)	97.39	
Neurological and neuromuscular	Walking/ Gait abnormality	Multicentre	n = 2	29/ 675	4.30 (0.40 to 33.37)	0	0.766
		Single-centre	n = 1	5/ 134	3.73 (1.56 to 8.65)	NA	
	Tremors	Multicentre	n = 2	17/ 586	2.98 (0.00 to 99.96)	94.5	0.615
		Single-centre	n = 1	25/ 538	4.65 (3.16 to 6.79)	NA	
	Headache	Single-centre	n = 6	95/3217	2.82 (0.69 to 10.88)	96.4	<0.001
		Multicentre	n = 4	65/ 884	7.35 (5.00 to 10.68)	37.26	
		Online survey	n = 1	67/ 434	15.44 (12.34 to 19.15)	NA	
	Smell disturbance	Multicentre	n = 6	197/1196	17.21 (13.03 to 22.38)	68.78	0.235
		Single-centre	n = 11	443/3935	12.49 (7.13 to 20.97)	95.97	
		Online survey	n = 2	202/ 537	27.06 (0.04 to 99.70)	96.07	
	Taste disturbance	Single-centre	n = 10	383/3825	12.21 (6.16 to 22.76)	97.3	0.793
		Multicentre	n = 5	143/1061	14.27 (10.13 to 19.73)	65.8	
		Online survey	n = 2	161/ 537	18.21 (0.00 to 99.91)	95.82	
	Tingling/ Paraesthesia	Single-centre	n = 1	4/ 122	3.28 (1.24 to 8.41)	NA	<0.001
		Multicentre	n = 1	29/ 135	21.48 (15.36 to 29.21)	NA	
	Decreased sensation or sensibility	Multicentre	n = 1	20/ 135	14.81 (9.76 to 21.85)	NA	0.060
		Single-centre	n = 1	10/ 134	7.46 (4.06 to 13.31)	NA	
Other	Hair loss	Multicentre	n = 1	14/ 118	11.86 (7.15 to 19.04)	NA	0.630
		Single-centre	n = 4	549/2692	14.99 (3.66 to 45.01)	95.58	
	Skin rash	Single-centre	n = 3	60/1923	3.53 (0.75 to 15.11)	82.97	0.112
		Multicentre	n = 1	7/ 451	1.55 (0.74 to 3.22)	NA	
Psychological and social	Care dependency	Single-centre	n = 2	146/2378	5.16 (0.00 to 99.97)	99.18	0.621
		Multicentre	n = 1	14/ 177	7.91 (4.74 to 12.91)	NA	
	PTSD	Multicentre	n = 2	54/ 706	7.65 (1.35 to 33.36)	0	0.653
		Single-centre	n = 4	275/1351	9.73 (1.74 to 39.56)	95.56	
	Sleep disorder	Single-centre	n = 7	665/3162	15.96 (6.78 to 33.15)	95.15	0.119
		Multicentre	n = 2	77/ 280	27.41 (2.84 to 82.98)	77.28	
	Depression	Single-centre	n = 5	474/3527	8.06 (3.47 to 17.62)	97.89	0.979

		Multicentre	n = 1	11/ 135	8.15 (4.57 to 14.11)	NA	
	Anxiety	Single-centre	n = 6	626/3416	18.92 (7.55 to 40.00)	97.66	0.870
		Multicentre	n = 1	24/ 135	17.78 (12.21 to 25.16)	NA	
Systemic	Dizziness	Single-centre	n = 3	124/2572	3.55 (1.05 to 11.30)	87.17	0.138
		Online survey	n = 1	29/ 434	6.68 (4.68 to 9.45)	NA	
		Multicentre	n = 1	9/ 135	6.67 (3.50 to 12.32)	NA	
	Sweat or night sweats	Multicentre	n = 1	35/ 145	24.14 (17.87 to 31.76)	NA	0.894
		Single-centre	n = 1	127/ 538	23.61 (20.21 to 27.38)	NA	
	Fever	Single-centre	n = 4	22/2621	0.98 (0.06 to 14.94)	94.58	<0.001
		Online survey	n = 1	22/ 434	5.07 (3.36 to 7.58)	NA	
		Multicentre	n = 2	3/ 569	0.53 (0.00 to 89.24)	0	
	Fatigue	Single-centre	n = 10	1781/4352	36.55 (25.00 to 49.88)	98.57	0.067
		Multicentre	n = 5	297/1150	24.28 (17.14 to 33.19)	78.24	
		Online survey	n = 2	129/ 537	24.02 (8.05 to 53.30)	0	
Upper respiratory	Other respiratory symptoms	Single-centre	n = 2	240/ 660	32.43 (2.22 to 91.02)	88.57	<0.001
		Multicentre	n = 1	13/ 451	2.88 (1.68 to 4.90)	NA	
	Nasal congestion	Online survey	n = 1	23/ 434	5.30 (3.55 to 7.85)	NA	0.690
		Multicentre	n = 2	27/ 569	4.75 (0.41 to 37.90)	25.27	
	Sore throat	Single-centre	n = 3	103/2327	5.38 (1.20 to 20.94)	90.55	0.538
		Multicentre	n = 2	24/ 569	4.22 (0.31 to 38.40)	0.31	

Michelen M, *et al. BMJ Global Health* 2021; 6:e005427. doi: 10.1136/bmjgh-2021-005427

Supplement 7: Subgroup analysis: Continents

Classification	Symptom	Subgroup	N Studies	Event/total	Proportion% (95% CIs)	Heterogeneity (%)	P value
Cardiopulmonary	Cough	Europe	n = 11	267/3074	9.71 (4.88 to 18.39)	94.92	0.057
		Asia	n = 4	146/1839	7.64 (4.60 to 12.44)	79.54	
		North America	n = 1	1/ 118	0.85 (0.12 to 5.77)	NA	
	Chest pain	Asia	n = 3	157/2840	4.11 (0.23 to 44.11)	97.52	0.097
		Europe	n = 7	135/1920	6.93 (2.77 to 16.31)	89.56	
		North America	n = 1	16/ 118	13.56 (8.48 to 20.99)	NA	
	Breathlessness/ Exertional dyspnoea	Europe	n = 13	868/3336	28.59 (18.52 to 41.35)	96.32	0.227
		Asia	n = 4	328/1839	16.53 (7.91 to 31.34)	95.25	
		Middle East	n = 2	70/ 230	22.35 (0.00 to 99.99)	97.16	
		North America	n = 1	31/ 118	26.27 (19.13 to 34.93)	NA	
	Other cardiovascular symptoms	Europe	n = 1	1/ 767	0.13 (0.02 to 0.92)	NA	0.009
		Asia	n = 2	78/1185	4.20 (0.00 to 99.97)	97.68	
	Palpitations	Asia	n = 4	387/3380	12.04 (7.03 to 19.85)	93.93	0.168
		Europe	n = 3	82/1280	8.10 (1.14 to 40.21)	95.9	
		North America	n = 1	7/ 118	5.93 (2.85 to 11.92)	NA	
	Excessive sputum/ Expectoration	Asia	n = 3	81/1192	6.56 (1.54 to 23.96)	90.08	0.238
		Europe	n = 3	32/ 757	4.23 (1.99 to 8.76)	0	
Gastrointestinal	Stomach/ Abdominal pain	Europe	n = 3	22/1310	1.61 (0.22 to 10.84)	80.31	0.011
		Asia	n = 1	8/ 117	6.84 (3.46 to 13.08)	NA	
	Loss of appetite	Asia	n = 2	166/1772	13.98 (0.06 to 97.66)	96.44	0.088
		Europe	n = 1	36/ 134	26.87 (20.05 to 34.99)	NA	
	Diarrhoea	Europe	n = 6	49/1495	2.37 (0.80 to 6.77)	80.93	0.055
		Asia	n = 3	133/2312	7.38 (2.34 to 20.91)	89.09	
		North America	n = 1	8/ 118	6.78 (3.43 to 12.97)	NA	
	Nausea or Vomiting	Europe	n = 2	20/ 586	3.92 (0.00 to 98.84)	92.31	0.004
		Asia	n = 1	21/ 117	17.95 (12.00 to 25.97)	NA	
		North America	n = 1	8/ 118	6.78 (3.43 to 12.97)	NA	
Musculoskeletal	Impaired mobility	Asia	n = 2	135/2162	5.63 (0.46 to 43.57)	82.31	<0.001
		Europe	n = 3	126/ 577	16.90 (2.10 to 65.88)	92.96	

		Middle East	n = 1	62/ 127	48.82 (40.25 to 57.46)	NA	
	Joint pain/ Arthralgia	Asia	n = 3	326/2733	12.25 (3.07 to 38.13)	97.8	0.295
		Europe	n = 6	111/1227	8.03 (4.01 to 15.42)	88.36	
	Muscle pain/ Myalgia	Europe	n = 9	294/2471	14.02 (7.27 to 25.34)	96.38	<0.001
		Asia	n = 2	63/2193	3.11 (0.16 to 38.54)	83.96	
		North America	n = 1	21/ 118	17.80 (11.90 to 25.76)	NA	
Neurocognitive	Other cognitive impairment	Europe	n = 2	118/ 264	40.19 (0.00 to 100.00)	99	0.017
		North America	n = 1	4/ 177	2.26 (0.85 to 5.86)	NA	
	Memory impairment	North America	n = 1	20/ 118	16.95 (11.20 to 24.82)	NA	0.898
		Europe	n = 4	131/ 768	18.19 (3.02 to 61.38)	96.14	
Neurological and neuromuscular	Walking/ Gait abnormality	Europe	n = 2	12/ 269	4.46 (0.11 to 66.56)	0	0.796
		Asia	n = 1	22/ 540	4.07 (2.70 to 6.11)	NA	
	Tremors	Europe	n = 2	17/ 586	2.98 (0.00 to 99.96)	94.5	0.615
		Asia	n = 1	25/ 538	4.65 (3.16 to 6.79)	NA	
	Headache	Asia	n = 1	33/1655	1.99 (1.42 to 2.79)	NA	0.005
		Europe	n = 9	187/2762	5.30 (2.12 to 12.66)	92.94	
		North America	n = 1	7/ 118	5.93 (2.85 to 11.92)	NA	
	Smell disturbance	Europe	n = 15	602/3615	15.35 (9.88 to 23.06)	96.43	0.027
		Asia	n = 1	176/1655	10.63 (9.24 to 12.21)	NA	
		North America	n = 2	49/ 295	16.74 (1.75 to 69.42)	65.9	
		Middle East	n = 1	15/ 103	14.56 (8.97 to 22.76)	NA	
	Taste disturbance	Asia	n = 1	120/1655	7.25 (6.10 to 8.60)	NA	<0.001
		Europe	n = 13	510/3370	14.25 (8.44 to 23.06)	96.81	
		North America	n = 2	49/ 295	16.74 (1.75 to 69.42)	65.9	
		Middle East	n = 1	8/ 103	7.77 (3.93 to 14.77)	NA	
Other	Hair loss	North America	n = 1	14/ 118	11.86 (7.15 to 19.04)	NA	0.005
		Asia	n = 2	513/2193	24.69 (5.86 to 63.32)	90.76	
		Europe	n = 2	36/ 499	8.21 (0.00 to 99.89)	96.66	
	Skin rash	Asia	n = 1	47/1655	2.84 (2.14 to 3.76)	NA	0.952
		Europe	n = 3	20/ 719	2.75 (0.30 to 20.89)	86.08	
Psychosological and social	Care dependency	Asia	n = 1	25/1611	1.55 (1.05 to 2.29)	NA	<0.001
		North America	n = 1	14/ 177	7.91 (4.74 to 12.91)	NA	

		Europe	n = 1	121/ 767	15.78 (13.36 to 18.53)	NA	
	Reduced quality of life	Europe	n = 1	28/90	31.11 (22.42 to 41.37)	NA	<0.001
		North America	n = 1	53/ 177	29.94 (23.66 to 37.09)	NA	
		Asia	n = 1	259/ 540	47.96 (43.77 to 52.18)	NA	
	Low mood/ Dysphoria	Europe	n = 2	53/ 360	0.86 (0.00 to 100.00)	0	0.868
		Asia	n = 1	9/ 538	1.67 (0.87 to 3.18)	NA	
	PTSD	Europe	n = 5	322/1937	9.93 (3.21 to 26.84)	96.87	0.322
		Middle East	n = 1	7/ 120	5.83 (2.81 to 11.73)	NA	
	Sleep disorder	Asia	n = 2	532/2193	22.00 (2.69 to 74.18)	94	0.486
		Europe	n = 7	210/1249	17.09 (7.01 to 36.03)	94.68	
	Depression	Asia	n = 2	390/2155	10.38 (0.00 to 99.83)	98.62	0.506
		Europe	n = 4	95/1507	6.80 (3.99 to 11.37)	71	
	Anxiety	Asia	n = 2	402/2155	12.63 (0.02 to 98.99)	98.36	0.320
		Europe	n = 5	248/1396	21.85 (8.03 to 47.22)	97.39	
Systemic	Dizziness	Asia	n = 2	115/2193	4.21 (0.08 to 71.53)	89.39	0.764
		Europe	n = 3	47/ 948	4.76 (1.43 to 14.71)	75.61	
	General malaise	Europe	n = 1	25/ 134	18.66 (12.93 to 26.16)	NA	<0.001
		Asia	n = 1	267/ 538	49.63 (45.42 to 53.85)	NA	
	Sweat or night sweats	Europe	n = 1	35/ 145	24.14 (17.87 to 31.76)	NA	0.894
		Asia	n = 1	127/ 538	23.61 (20.21 to 27.38)	NA	
	Fever	Europe	n = 5	44/1851	1.91 (0.36 to 9.62)	90.51	0.011
		Asia	n = 1	2/1655	0.12 (0.03 to 0.48)	NA	
		North America	n = 1	1/ 118	0.85 (0.12 to 5.77)	NA	
	Fatigue	Europe	n = 8	605/2014	34.68 (25.12 to 45.66)	93	0.382
		North America	n = 2	60/ 295	20.71 (0.25 to 96.48)	91.68	
		Asia	n = 4	1436/3380	31.33 (10.49 to 63.98)	99.42	
		Middle East	n = 3	106/ 350	28.07 (6.94 to 67.15)	93.59	
Upper respiratory	Other respiratory symptoms	Europe	n = 2	43/ 573	8.88 (0.00 to 99.98)	97.85	0.029
		Asia	n = 1	210/ 538	39.03 (35.00 to 43.22)	NA	
	Nasal congestion	Europe	n = 2	42/ 885	4.75 (0.66 to 27.08)	0	0.343
		North America	n = 1	8/ 118	6.78 (3.43 to 12.97)	NA	
	Sore throat	Asia	n = 2	86/2193	3.92 (1.00 to 14.17)	8.33	0.165

	Europe	n = 2	38/ 585	7.48 (0.06 to 91.61)	90.13
	North America	n = 1	3/ 118	2.54 (0.82 to 7.59)	NA

Supplement 8: Subgroup analysis: Follow-up timing

Classification	Symptom	Subgroup	N Studies	Event/total	Proportion% (95% CIs)	Heterogeneity (%)	P value
Cardiopulmonary	Cough	< 4 months	n = 14	374/4483	8.35 (4.56 to 14.81)	94.34	0.669
		> 4 months	n = 2	40/548	7.30 (0.97 to 38.82)	0	
	Chest pain	> 4 months	n = 1	75/1655	4.53 (3.63 to 5.65)	NA	0.311
		< 4 months	n = 10	233/3223	6.55 (2.97 to 13.84)	91.52	
	Breathlessness/ Exertional dyspnoea	< 4 months	n = 15	1142/4562	28.92 (20.29 to 39.41)	96.15	0.075
		> 4 months	n = 5	155/961	15.41 (5.74 to 35.30)	95.81	
	Palpitations	> 4 months	n = 1	154/1655	9.31 (8.00 to 10.80)	NA	0.863
		< 4 months	n = 7	322/3123	9.71 (5.42 to 16.78)	94.42	
	Excessive sputum/ Expectoration	> 4 months	n = 1	11/114	9.65 (5.42 to 16.59)	NA	0.069
		< 4 months	n = 5	102/1835	4.95 (2.64 to 9.09)	85.74	
Gastrointestinal	Weight loss	> 4 months	n = 1	47/434	10.83 (8.23 to 14.12)	NA	<0.001
		< 4 months	n = 1	50/134	37.31 (29.55 to 45.79)	NA	
	Loss of appetite	> 4 months	n = 1	138/1655	8.34 (7.10 to 9.77)	NA	<0.001
		< 4 months	n = 2	64/251	25.50 (5.15 to 68.31)	0	
	Diarrhoea	< 4 months	n = 8	85/1836	3.53 (1.41 to 8.59)	84.4	0.371
		> 4 months	n = 2	105/2089	5.03 (1.46 to 15.89)	0	
Musculoskeletal	Impaired mobility	> 4 months	n = 3	241/1953	24.29 (2.15 to 82.41)	99.05	0.108
		< 4 months	n = 3	82/913	8.07 (0.95 to 44.49)	96.49	
	Joint pain/ Arthralgia	> 4 months	n = 1	154/1655	9.31 (8.00 to 10.80)	NA	0.986
		< 4 months	n = 8	283/2305	9.35 (5.22 to 16.17)	94.04	
	Muscle pain/ Myalgia	< 4 months	n = 11	339/3127	12.95 (7.31 to 21.91)	96.15	<0.001
		> 4 months	n = 1	39/1655	2.36 (1.73 to 3.21)	NA	
Neurocognitive	Other cognitive impairment	< 4 months	n = 2	118/264	40.19 (0.00 to 100.00)	99	0.017
		> 4 months	n = 1	4/177	2.26 (0.85 to 5.86)	NA	
Neurological and neuromuscular	Headache	> 4 months	n = 3	113/2269	6.11 (0.65 to 39.33)	97.98	0.620
		< 4 months	n = 8	114/2266	4.42 (1.60 to 11.59)	92.68	
	Smell disturbance	< 4 months	n = 13	367/2994	13.26 (8.37 to 20.37)	94.8	0.166
		> 4 months	n = 6	475/2674	19.96 (11.27 to 32.87)	97.79	
	Taste disturbance	> 4 months	n = 6	364/2674	15.36 (7.94 to 27.63)	97.61	0.580

		< 4 months	n = 11	323/2749	12.61 (6.95 to 21.81)	96.44	
Other	Hair loss	< 4 months	n = 4	204/1155	12.72 (3.10 to 39.89)	95.98	0.181
		> 4 months	n = 1	359/1655	21.69 (19.77 to 23.74)	NA	
	Skin rash	> 4 months	n = 1	47/1655	2.84 (2.14 to 3.76)	NA	0.952
		< 4 months	n = 3	20/719	2.75 (0.30 to 20.89)	86.08	
Psychological and social	Care dependency	> 4 months	n = 2	39/1788	3.38 (0.00 to 98.56)	95.89	0.006
		< 4 months	n = 1	121/767	15.78 (13.36 to 18.53)	NA	
	Reduced quality of life	< 4 months	n = 2	287/630	40.64 (2.65 to 94.52)	88.36	0.119
		> 4 months	n = 1	53/177	29.94 (23.66 to 37.09)	NA	
	PTSD	< 4 months	n = 5	322/1937	9.93 (3.21 to 26.84)	96.87	0.322
		> 4 months	n = 1	7/120	5.83 (2.81 to 11.73)	NA	
	Sleep disorder	> 4 months	n = 1	437/1655	26.40 (24.34 to 28.58)	NA	0.130
		< 4 months	n = 8	305/1787	17.20 (8.21 to 32.54)	94.08	
	Depression	> 4 months	n = 1	367/1617	22.70 (20.72 to 24.80)	NA	<0.001
		< 4 months	n = 5	118/2045	6.16 (3.99 to 9.40)	71.69	
	Anxiety	> 4 months	n = 1	367/1617	22.70 (20.72 to 24.80)	NA	0.492
		< 4 months	n = 6	283/1934	18.10 (7.16 to 38.77)	97.51	
Systemic	Dizziness	> 4 months	n = 2	130/2089	6.22 (2.06 to 17.34)	0	0.011
		< 4 months	n = 3	32/1052	3.20 (1.08 to 9.12)	68.78	
	Fever	< 4 months	n = 5	23/1535	1.26 (0.22 to 7.01)	90.73	0.762
		> 4 months	n = 2	24/2089	0.79 (0.00 to 100.00)	96.18	
	Fatigue	< 4 months	n = 10	890/3243	32.50 (23.93 to 42.42)	94.57	0.608
		> 4 months	n = 7	1317/2796	28.61 (16.00 to 45.73)	98.47	
Upper respiratory	Nasal congestion	> 4 months	n = 1	23/434	5.30 (3.55 to 7.85)	NA	0.690
		< 4 months	n = 2	27/569	4.75 (0.41 to 37.90)	25.27	
	Sore throat	> 4 months	n = 1	69/1655	4.17 (3.31 to 5.25)	NA	0.655
		< 4 months	n = 4	58/1241	4.84 (1.76 to 12.65)	85.28	

Michelen M, *et al. BMJ Global Health* 2021; 6:e005427. doi: 10.1136/bmjgh-2021-005427

Supplement 9: Meta-regression: % Female

Classification	Symptom	N Studies	Constant (SE)	Beta (SE)	R^2	P value
Cardiopulmonary	Breathlessness/ Exertional dyspnoea	20	-0.23 (0.79)	-1.93 (1.71)	0.07	0.258
	Chest pain	11	-2.13 (1.85)	-1.27 (4.11)	0.01	0.758
	Cough	16	-2.43 (1.12)	0.02 (2.32)	0.00	0.994
Gastrointestinal	Diarrhoea	10	-3.9 (1.46)	1.46 (2.87)	0.00	0.612
Systemic	Fatigue	17	-0.08 (0.61)	-1.58 (1.29)	0.09	0.222
Musculoskeletal	Muscle pain/ Myalgia	12	-0.37 (1.33)	-3.97 (3.05)	0.13	0.194
Neurological and neuromuscular	Headache	11	-6.29 (1.27)	6.7 (2.46)	0.43	0.007
	Smell disturbance	19	-4.07 (0.53)	4.95 (1.08)	0.56	<0.001
	Taste disturbance	17	-4.29 (0.63)	5.04 (1.27)	0.51	<0.001

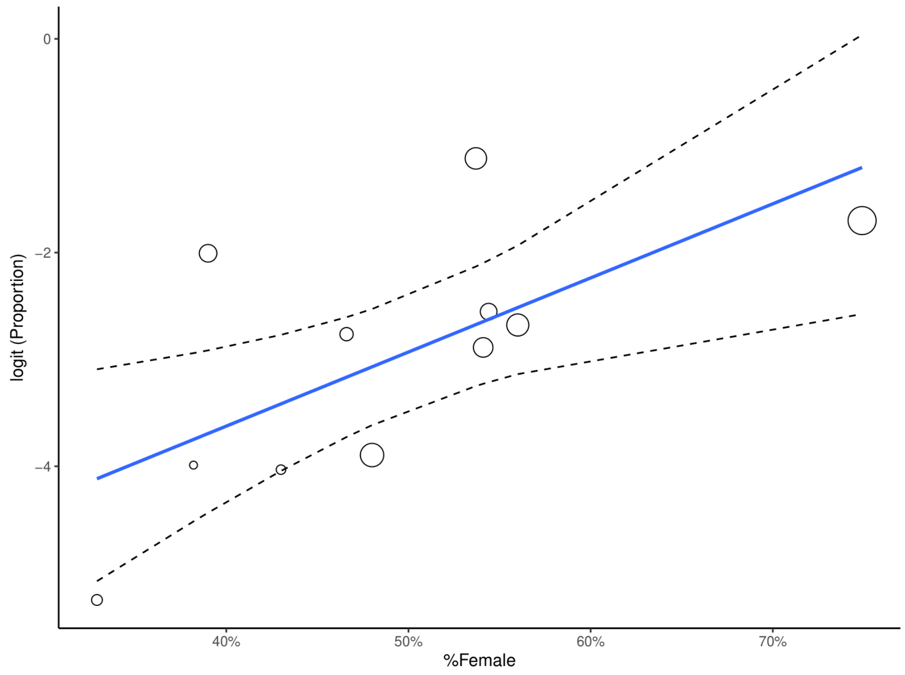

Figure 13. Metaregression on percentage of female. Neurological and neuromuscular (Headache)

The bubble plot presents the association between the proportions of females and people experienced headache (p<0.05). Each circle represents the value of an individual study, and the size of each circle is proportional to the study weight by inverse-variance weighting.

Michelen M, *et al. BMJ Global Health* 2021; 6:e005427. doi: 10.1136/bmjgh-2021-005427

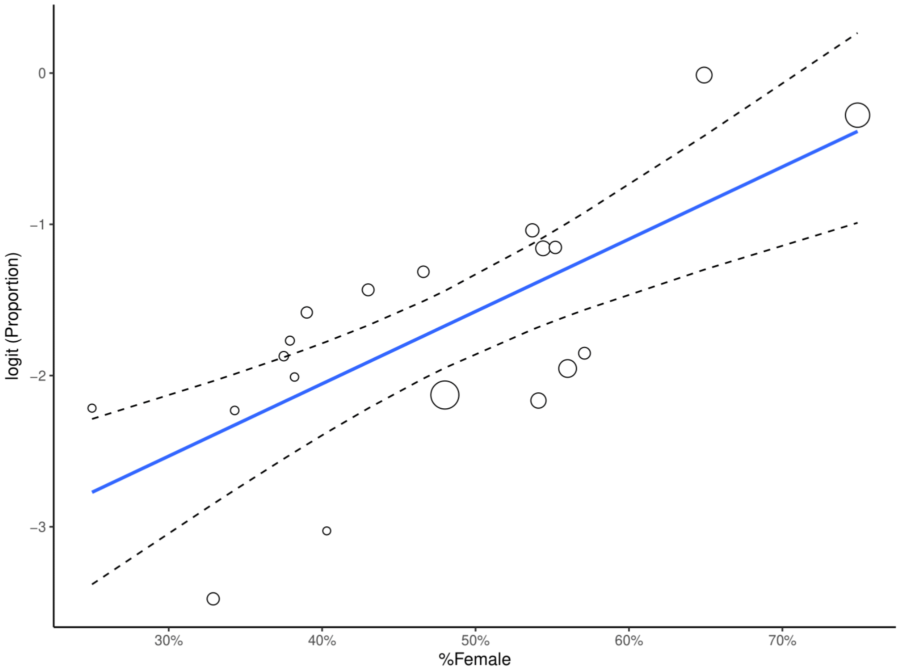

Figure 14. Metaregression on percentage of female. Neurological and neuromuscular (Small disturbance).

The bubble plot presents the association between the proportions of females and people experienced smell disturbance (p<0.05). Each circle represents the value of an individual study, and the size of each circle is proportional to the study weight by inverse-variance weighting.

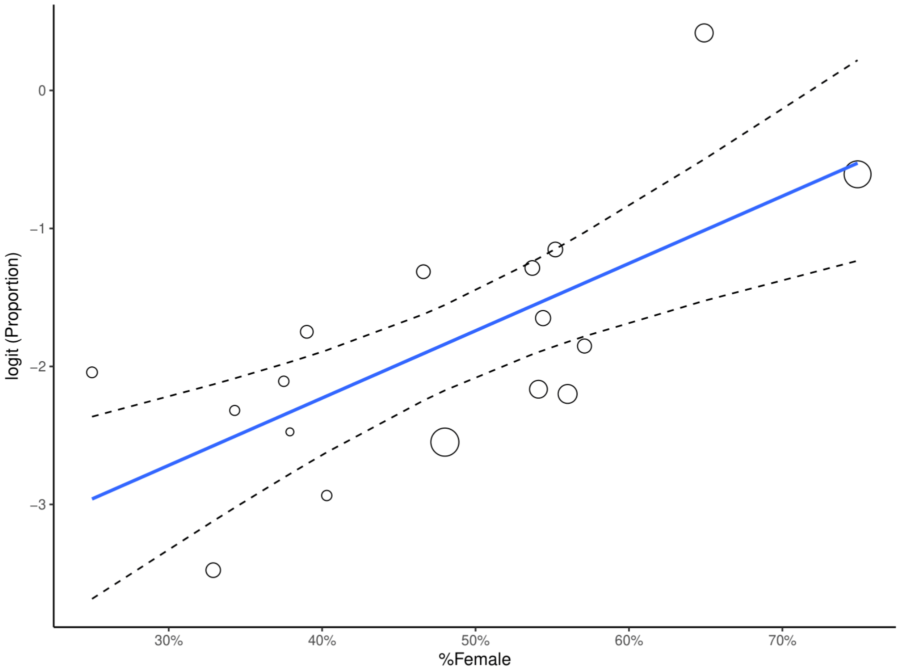

Figure 15. Metaregression on percentage of female. Neurological and neuromuscular (Taste disturbance).

The bubble plot presents the association between the proportions of females and people experienced taste disturbance (p<0.05). Each circle represents the value of an individual study, and the size of each circle is proportional to the study weight by inverse-variance weighting.

Supplement 10: Meta-regression: % ICU patients

Classification	Symptom	N Studies	Constant (SE)	Beta (SE)	R^2	P value
Cardiopulmonary	Breathlessness/ Exertional dyspnoea	14	-1.03 (0.31)	1.02 (0.89)	0.09	0.254
	Cough	11	-2.25 (0.63)	-0.75 (2.73)	-0.01*	0.783
Systemic	Fatigue	11	-0.67 (0.29)	0.4 (0.81)	0.02	0.620
Musculoskeletal	Muscle pain/ Myalgia	11	-2.71 (0.46)	4.19 (2.12)	0.27	0.048
Neurological and neuromuscular	Smell disturbance	14	-2.24 (0.26)	1.32 (1.3)	0.08	0.311
	Taste disturbance	12	-2.45 (0.25)	1.72 (1.23)	0.16	0.161

*poor fitting

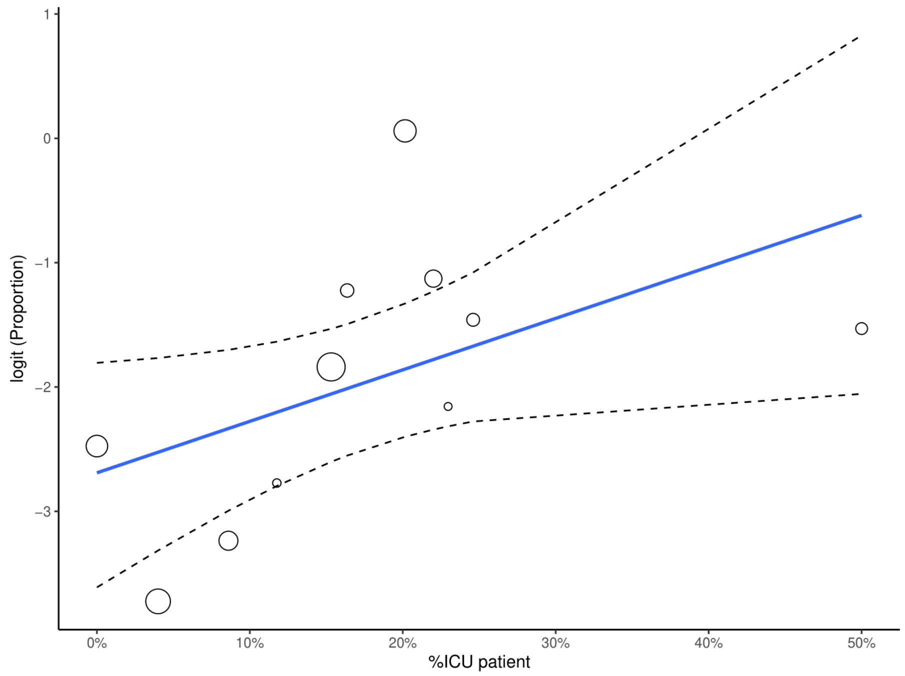

Figure 16. Metaregression on percentage of ICU patients. Musculoskeletal (Muscle pain/ Myalgia).

The bubble plot presents the association between the proportions of ICU patients and patients experienced muscle pain/myalgia (p<0.05). Each circle represents the value of an individual study, and the size of each circle is proportional to the study weight by inverse-variance weighting.

Michelen M, *et al. BMJ Global Health* 2021; 6:e005427. doi: 10.1136/bmjgh-2021-005427

Supplement 11: Sensitivity analysis: versus removing high risk of bias studies

Classification	Symptom	Main results			Main results after removing high risk of bias studies		
		N Studies	n/Total	Prop (95% CIs)	N Studies	n/Total	Prop (95% CIs)
Cardiopulmonary	Other cardiovascular symptoms	n = 3	79/1952	1.38 (0.01 to 67.44)	n = 2	71/1305	1.37 (0.04 to 32.38)
	Palpitations	n = 8	476/4778	9.67 (5.95 to 15.34)	n = 7	413/4131	9.65 (5.39 to 16.66)
	Excessive sputum/ Expectoration	n = 6	113/1949	5.46 (3.19 to 9.19)	n = 4	88/1326	6.22 (2.62 to 14.05)
	Cough	n = 16	414/5031	8.17 (4.85 to 13.44)	n = 11	265/3207	7.42 (3.38 to 15.55)
	Chest pain	n = 11	308/4878	6.36 (3.15 to 12.42)	n = 8	275/3939	7.33 (3.25 to 15.68)
	Breathlessness/ Exertional dyspnoea	n = 20	1297/5523	25.06 (17.86 to 33.97)	n = 14	992/3596	28.85 (19.67 to 40.16)
Gastrointestinal	Weight loss	n = 2	97/568	20.99 (8.09 to 44.51)	n = 1	50/134	37.31 (29.12 to 46.08)
	Stomach/ Abdominal pain	n = 4	30/1427	2.33 (0.54 to 9.42)	n = 2	7/859	0.81 (0.39 to 1.70)
	Loss of appetite	n = 3	202/1906	17.49 (4.13 to 51.04)	n = 2	174/1789	15.09 (6.35 to 31.80)
	Diarrhoea	n = 10	190/3925	4.00 (2.07 to 7.57)	n = 6	140/2751	4.68 (2.49 to 8.66)
	Nausea or Vomiting	n = 4	49/821	6.69 (1.64 to 23.59)	n = 2	20/253	7.91 (5.16 to 11.93)
Musculoskeletal	Impaired mobility	n = 6	323/2866	14.42 (4.67 to 36.73)	n = 5	257/2662	12.00 (3.02 to 37.39)
	Joint pain/ Arthralgia	n = 9	437/3960	9.39 (5.72 to 15.03)	n = 6	378/3215	10.79 (5.21 to 21.00)
	Muscle pain/ Myalgia	n = 12	378/4782	11.29 (6.17 to 19.75)	n = 10	320/4209	11.14 (5.35 to 21.75)
Neurocognitive	Other cognitive impairment	n = 3	122/441	17.77 (0.08 to 98.23)	n = 3	122/441	17.77 (0.08 to 98.23)
	Confusion	n = 2	33/1218	2.71 (1.93 to 3.79)	n = 1	23/767	3.00 (1.91 to 4.47)
	Concentration impairment	n = 2	66/254	25.98 (20.96 to 31.73)	n = 1	34/134	25.37 (18.26 to 33.61)
	Memory impairment	n = 5	151/886	17.94 (5.26 to 46.25)	n = 4	110/766	14.93 (2.77 to 51.97)
Neurological and neuromuscular	Tingling/ Paraesthesia	n = 2	33/257	9.12 (2.21 to 30.87)	n = 1	29/135	21.48 (14.88 to 29.37)
	Visual disturbance	n = 2	28/586	4.78 (3.32 to 6.83)	n = 1	9/135	6.67 (3.09 to 12.28)
	Smell disturbance	n = 19	842/5668	15.17 (10.75 to 20.97)	n = 13	513/4258	14.08 (8.87 to 21.62)
	Taste disturbance	n = 17	687/5423	13.52 (8.96 to 19.89)	n = 11	425/4013	13.44 (7.31 to 23.42)
	Tremors	n = 3	42/1124	3.53 (0.30 to 30.63)	n = 2	38/673	6.20 (3.68 to 10.26)
	Headache	n = 11	227/4535	4.88 (2.30 to 10.06)	n = 7	115/3298	4.19 (1.30 to 12.71)
Other	Hair loss	n = 5	563/2810	14.34 (5.33 to 33.23)	n = 4	539/2690	13.14 (3.17 to 41.12)

	Skin rash	n = 4	67/2374	2.83 (0.95 to 8.16)	n = 3	60/1923	3.53 (0.75 to 15.11)
Psychological and social	Sleep disorder	n = 9	742/3442	18.15 (9.61 to 31.63)	n = 8	705/3322	16.88 (8.18 to 31.65)
Systemic	Dizziness	n = 5	162/3141	4.50 (2.53 to 7.86)	n = 4	133/2707	4.02 (1.87 to 8.42)
	Fever	n = 7	47/3624	1.08 (0.24 to 4.66)	n = 5	23/2739	0.91 (0.11 to 7.18)
	Fatigue	n = 17	2207/6039	30.97 (23.91 to 39.03)	n = 12	1882/4555	33.24 (24.57 to 43.22)
Upper respiratory	Other respiratory symptoms	n = 3	253/1111	15.58 (0.68 to 83.17)	n = 1	210/538	39.03 (34.89 to 43.30)
	Nasal congestion	n = 3	50/1003	4.99 (2.73 to 8.92)	n = 1	8/118	6.78 (2.97 to 12.92)
	Sore throat	n = 5	127/2896	4.70 (2.42 to 8.91)	n = 4	106/2445	4.70 (1.73 to 12.10)

Supplement 12: Sensitivity analysis: versus statistical methods

Classification	Symptom	N Studies	n/Total	Main results	FTDAT/IV*
				Prop (95% CIs)	Prop (95% CIs)
Cardiopulmonary	Breathlessness/ Exertional dyspnoea	n = 20	1297/5523	25.06 (17.86 to 33.97)	26.68 (20.36 to 33.51)
	Palpitations	n = 8	476/4778	9.67 (5.95 to 15.34)	10.21 (6.76 to 14.26)
	Cough	n = 16	414/5031	8.17 (4.85 to 13.44)	9.52 (6.16 to 13.50)
	Chest pain	n = 11	308/4878	6.36 (3.15 to 12.42)	7.52 (4.29 to 11.52)
	Excessive sputum/ Expectoration	n = 6	113/1949	5.46 (3.19 to 9.19)	5.69 (3.23 to 8.75)
	Flushing	n = 1	26/538	4.83 (3.18 to 7.00)	4.83 (3.17 to 6.82)
	Newly diagnosed hypertension	n = 1	7/538	1.30 (0.52 to 2.66)	1.30 (0.49 to 2.46)
	Other cardiovascular symptoms	n = 3	79/1952	1.38 (0.01 to 67.44)	2.99 (0.00 to 12.59)
Gastrointestinal	Weight loss	n = 2	97/568	20.99 (8.09 to 44.51)	22.47 (3.00 to 52.50)
	Other stomach/ Abdominal discomfort	n = 1	21/117	17.95 (11.47 to 26.12)	17.95 (11.47 to 25.47)
	Loss of appetite	n = 3	202/1906	17.49 (4.13 to 51.04)	18.57 (6.35 to 35.21)
	Nausea or Vomiting	n = 4	49/821	6.69 (1.64 to 23.59)	7.69 (1.89 to 16.67)
	Diarrhoea	n = 10	190/3925	4.00 (2.07 to 7.57)	4.41 (2.65 to 6.57)
	Bloody stools / Haematochezia	n = 1	2/117	1.71 (0.21 to 6.04)	1.71 (0.03 to 5.08)
	Stomach/ Abdominal pain	n = 4	30/1427	2.33 (0.54 to 9.42)	2.63 (0.56 to 5.93)
Musculoskeletal	Impaired mobility	n = 6	323/2866	14.42 (4.67 to 36.73)	17.09 (7.35 to 29.77)
	Muscle pain/ Myalgia	n = 12	378/4782	11.29 (6.17 to 19.75)	13.09 (7.71 to 19.59)
	Joint pain/ Arthralgia	n = 9	437/3960	9.39 (5.72 to 15.03)	10.04 (6.33 to 14.46)
Neurocognitive	Concentration impairment	n = 2	66/254	25.98 (20.96 to 31.73)	25.98 (20.74 to 31.59)
	Memory impairment	n = 5	151/886	17.94 (5.26 to 46.25)	20.55 (6.54 to 39.62)
	Other cognitive impairment	n = 3	122/441	17.77 (0.08 to 98.23)	25.51 (0.00 to 79.72)
	Frontal release signs	n = 1	20/135	14.81 (9.29 to 21.95)	14.81 (9.27 to 21.35)
	Confusion	n = 2	33/1218	2.71 (1.93 to 3.79)	2.69 (1.84 to 3.69)
Neurological and neuromuscular	Abnormal reflex status	n = 1	31/135	22.96 (16.17 to 30.98)	22.96 (16.22 to 30.47)
	Other neurological diseases	n = 1	20/135	14.81 (9.29 to 21.95)	14.81 (9.27 to 21.35)
	Smell disturbance	n = 19	842/5668	15.17 (10.75 to 20.97)	16.48 (11.36 to 22.31)
	Taste disturbance	n = 17	687/5423	13.52 (8.96 to 19.89)	14.99 (9.76 to 21.09)
	Decreased sensation or sensibility	n = 2	30/269	10.90 (6.71 to 17.22)	10.88 (4.73 to 19.05)
	Tingling/ Paraesthesia	n = 2	33/257	9.12 (2.21 to 30.87)	10.74 (0.02 to 34.12)
	Muscle atrophy	n = 1	9/135	6.67 (3.09 to 12.28)	6.67 (2.98 to 11.58)
	Headache	n = 11	227/4535	4.88 (2.30 to 10.06)	6.12 (2.97 to 10.25)
	Slowness of movement/ Bradykinesia	n = 1	7/135	5.19 (2.11 to 10.39)	5.19 (1.98 to 9.67)
	Visual disturbance	n = 2	28/586	4.78 (3.32 to 6.83)	4.86 (2.79 to 7.43)
	Abnormal muscle tone	n = 1	6/135	4.44 (1.65 to 9.42)	4.44 (1.50 to 8.68)
	Tremors	n = 3	42/1124	3.53 (0.30 to 30.63)	4.12 (0.76 to 9.75)
	Walking/ Gait abnormality	n = 3	34/809	4.20 (2.02 to 8.53)	4.11 (2.80 to 5.63)

	Trigeminal neuralgia	n = 1	4/122	3.28 (0.90 to 8.18)	3.28 (0.71 to 7.33)
	Speech difficulty/ Dysarthria	n = 1	3/135	2.22 (0.46 to 6.36)	222 (0.28 to 5.56)
	Ear/ Hearing conditions	n = 1	5/451	1.11 (0.36 to 2.57)	1.11 (0.31 to 2.33)
	Lack of coordination/ Dysmetria	n = 1	2/135	1.48 (0.18 to 5.25)	1.48 (0.02 to 4.41)
	Seizures/ Cramps	n = 1	6/451	1.33 (0.49 to 2.87)	1.33 (0.44 to 2.63)
Other	Hair loss	n = 5	563/2810	14.34 (5.33 to 33.23)	15.86 (7.42 to 26.68)
	Skin rash	n = 4	67/2374	2.83 (0.95 to 8.16)	2.86 (1.29 to 4.96)
	Conjunctivitis	n = 1	8/451	1.77 (0.77 to 3.47)	1.77 (0.73 to 3.23)
Psychological and social	Reduced quality of life	n = 3	340/807	36.76 (18.43 to 59.93)	36.60 (23.89 to 50.32)
	Anxiety	n = 7	650/3551	18.73 (8.89 to 35.25)	20.39 (11.95 to 30.38)
	Sleep disorder	n = 9	742/3442	18.15 (9.61 to 31.63)	20.01 (12.32 to 28.99)
	PTSD	n = 6	329/2057	9.14 (3.66 to 21.04)	10.41 (3.36 to 20.59)
	Depression	n = 6	485/3662	8.06 (4.14 to 15.10)	8.72 (3.02 to 16.93)
	Care dependency	n = 3	160/2555	5.89 (0.46 to 45.96)	7.24 (0.36 to 21.24)
	Low mood/ Dysphoria	n = 3	62/898	1.79 (0.00 to 98.74)	7.49 (0.00 to 31.80)
Systemic	Weakness	n = 2	186/513	41.20 (25.43 to 59.01)	41.62 (19.16 to 66.08)
	General malaise	n = 2	292/672	32.68 (14.91 to 57.36)	33.47 (8.11 to 65.59)
	Fatigue	n = 17	2207/6039	30.97 (23.91 to 39.03)	31.75 (22.81 to 41.41)
	Sweat or night sweats	n = 2	162/683	23.72 (20.68 to 27.05)	23.68 (20.55 to 26.96)
	Dizziness	n = 5	162/3141	4.50 (2.53 to 7.86)	4.58 (2.80 to 6.76)
	Enlarged lymph nodes/ Lymphadenopathy	n = 1	4/451	0.89 (0.24 to 2.26)	0.89 (0.19 to 2.01)
	Fever	n = 7	47/3624	1.08 (0.24 to 4.66)	1.74 (0.28 to 4.14)
Upper respiratory	Other respiratory symptoms	n = 3	253/1111	15.58 (0.68 to 83.17)	19.30 (0.95 to 52.06)
	Voice change	n = 1	11/134	8.21 (4.17 to 14.21)	8.21 (4.08 to 13.53)
	Nasal congestion	n = 3	50/1003	4.99 (2.73 to 8.92)	4.89 (3.61 to 6.34)
	Sore throat	n = 5	127/2896	4.70 (2.42 to 8.91)	4.66 (2.94 to 6.74)

* FTDAT/IV: Freeman-Tukey Double arcsine transformation within inverse Variance Method

Supplement 13: Funnel plots

The following funnel plots present the proportion of people experienced with certain symptoms against the standard errors (Egger's method) or sample size (Peter's method) to assess potential publication bias and small study effects. Only symptoms reported 10 or more are presented here.

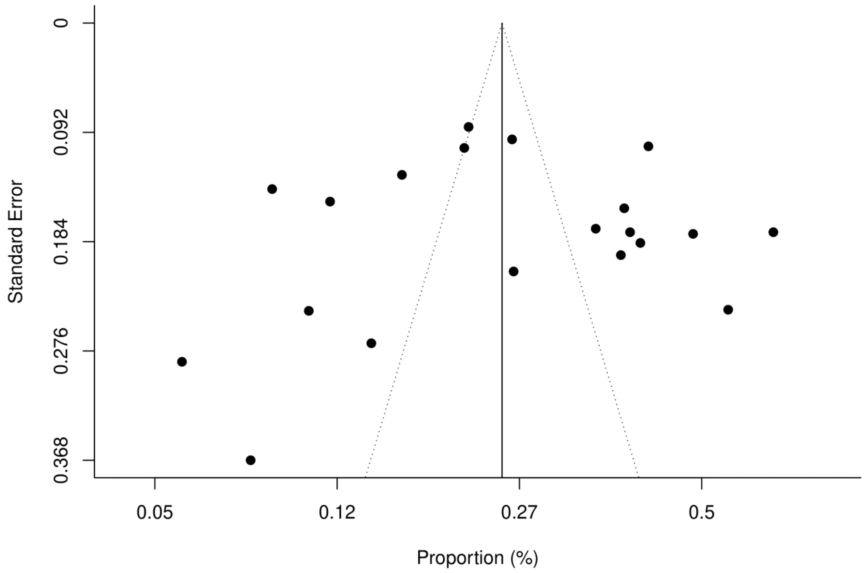

Figure 17. Funnel plot. Cardiopulmonary (Breathlessness or Exertional dyspnoea) by Egger's method

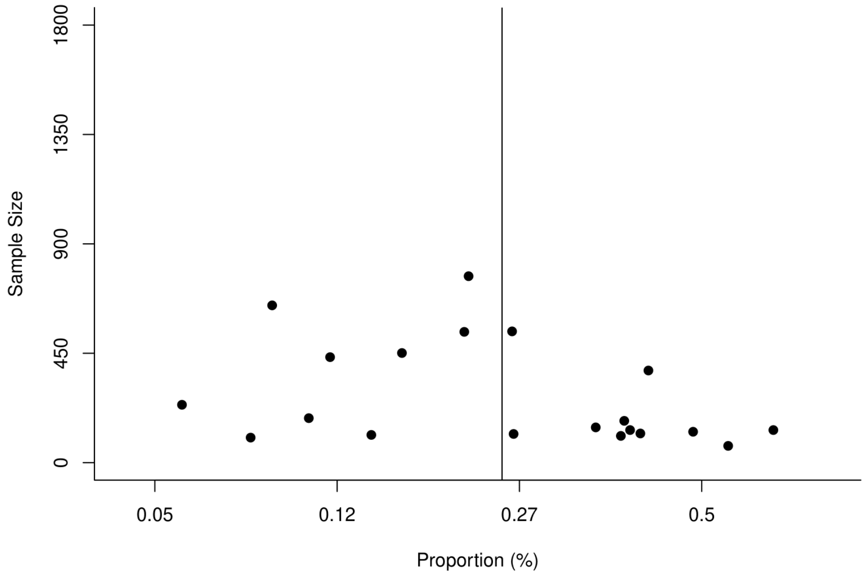

Figure 18. Funnel plot. Cardiopulmonary (Breathlessness or Exertional dyspnoea) by Peter's method

Michelen M, *et al. BMJ Global Health* 2021; 6:e005427. doi: 10.1136/bmjgh-2021-005427

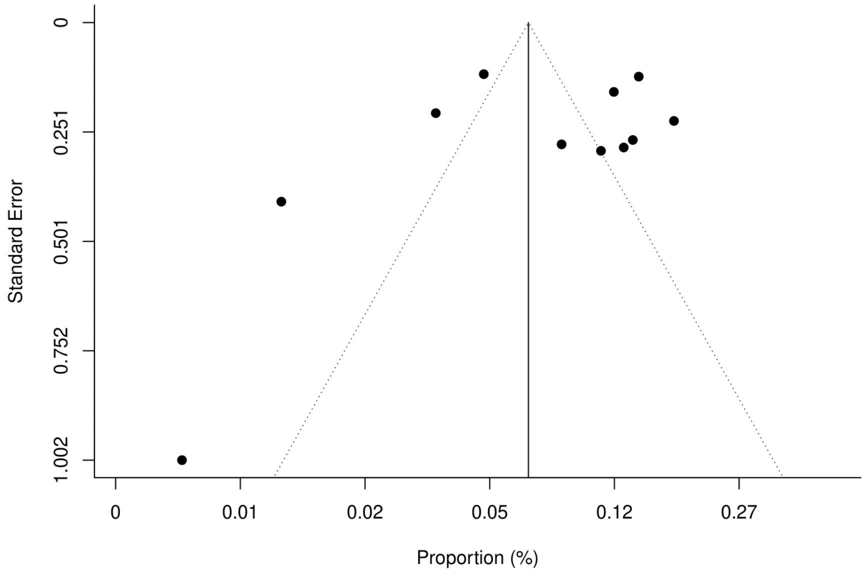

Figure 19. Funnel plot. Cardiopulmonary (Chest pain) by Egger's method

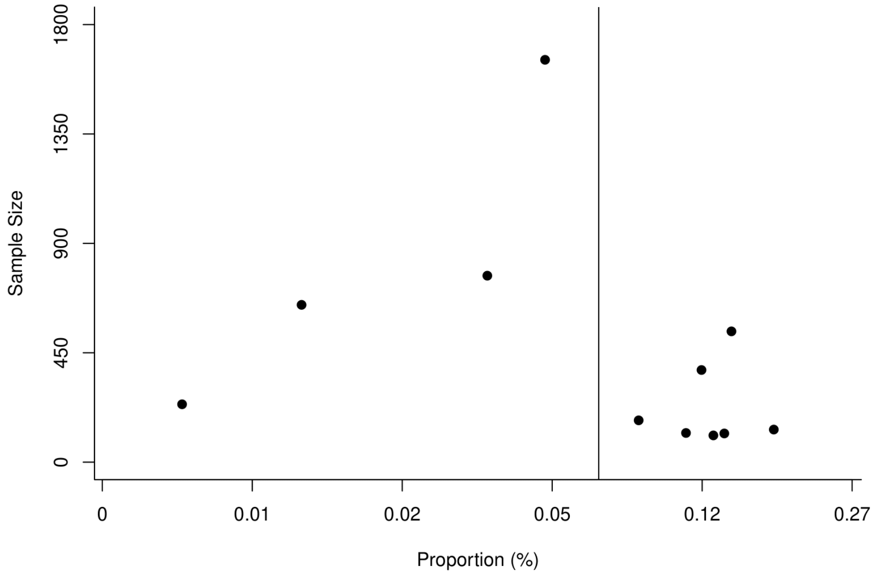

Figure 20. Funnel plot. Cardiopulmonary (Chest pain) by Peter's method

Michelen M, *et al. BMJ Global Health* 2021; 6:e005427. doi: 10.1136/bmjgh-2021-005427

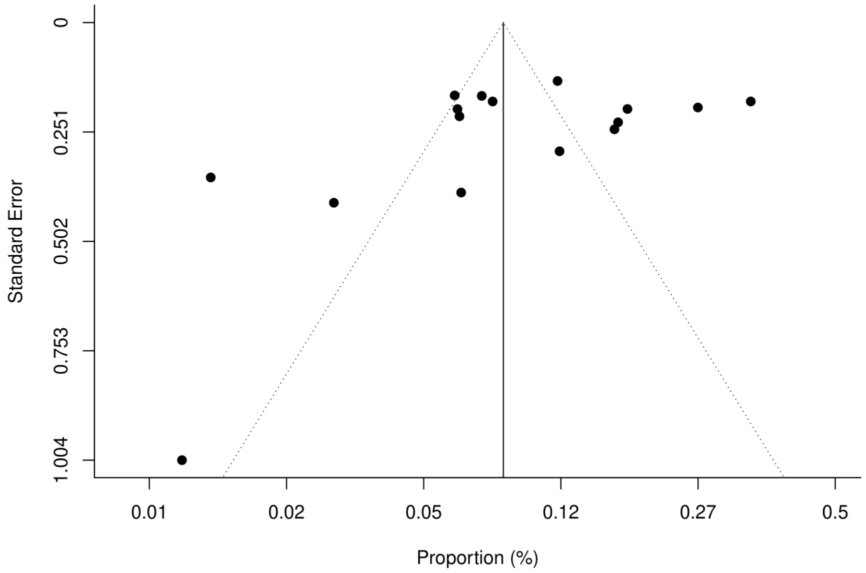

Figure 21. Funnel plot. Cardiopulmonary (Cough) by Egger's method

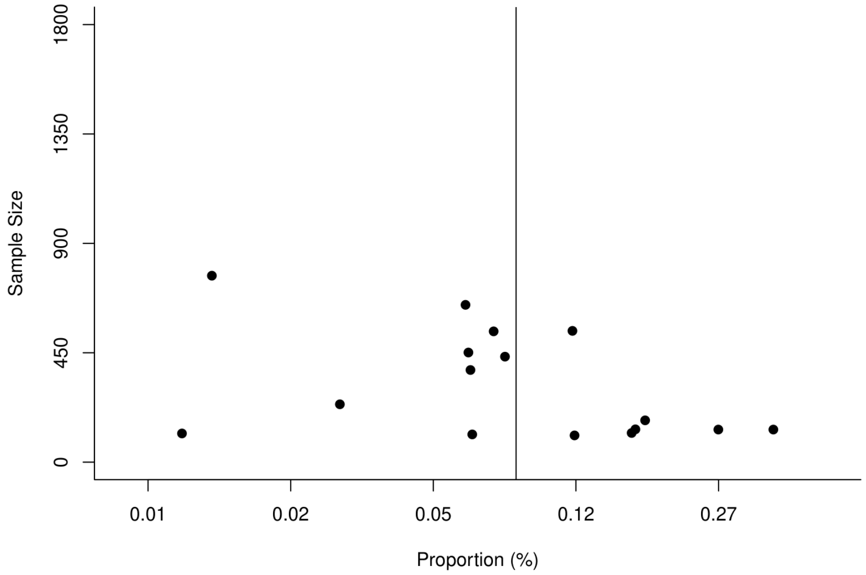

Figure 22. Funnel plot. Cardiopulmonary (Cough) by Peter's method

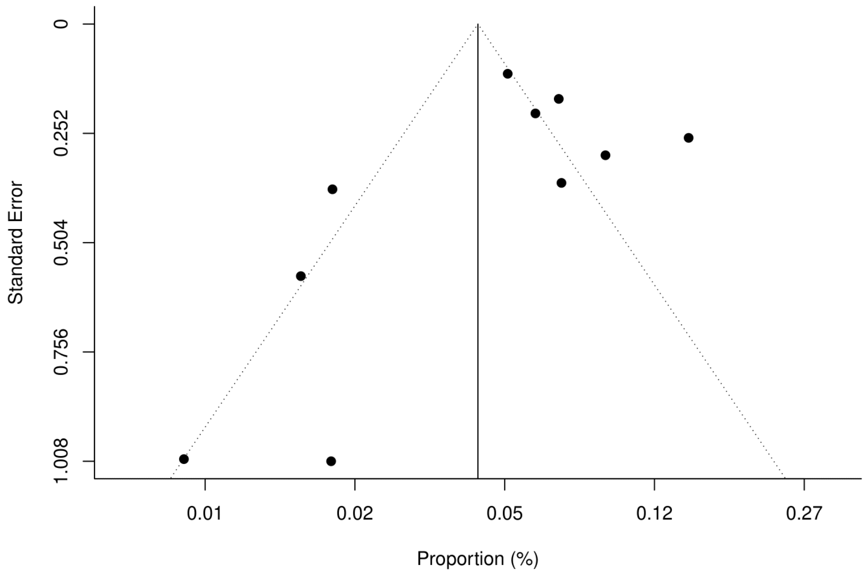

Figure 23. Funnel plot. Gastrointestinal (Diarrhoea) by Egger's method

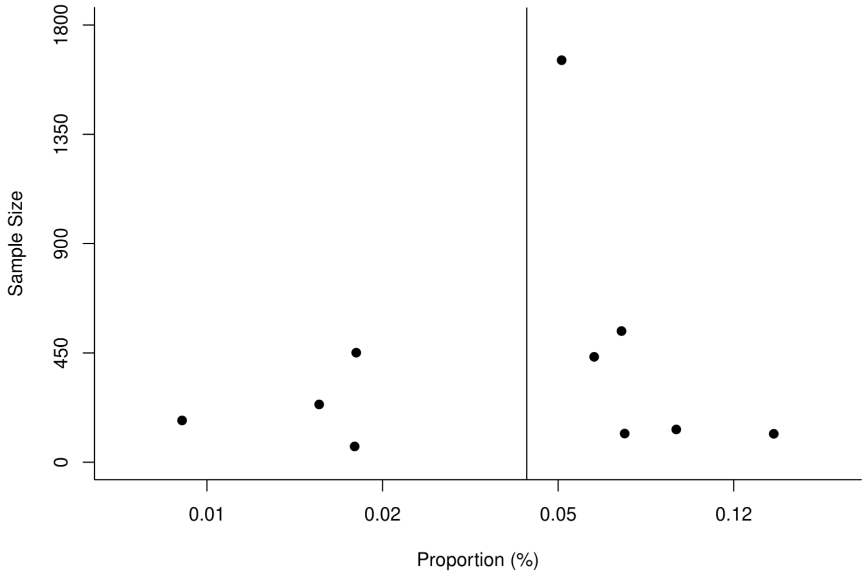

Figure 24. Funnel plot. Gastrointestinal (Diarrhoea) by Peter's method

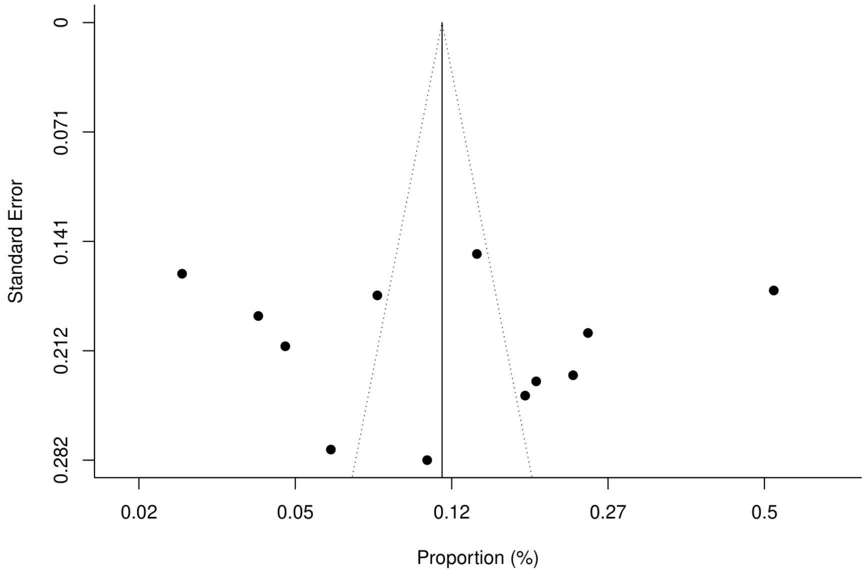

Figure 25. Funnel plot. Musculoskeletal (Muscle pain or Myalgia) by Egger's method

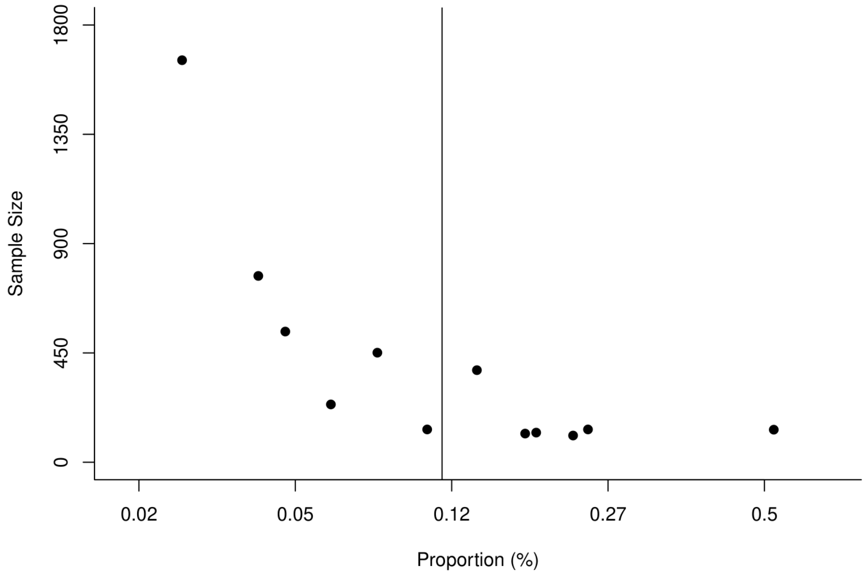

Figure 26. Funnel plot. Musculoskeletal (Muscle pain or Myalgia) by Peter's method

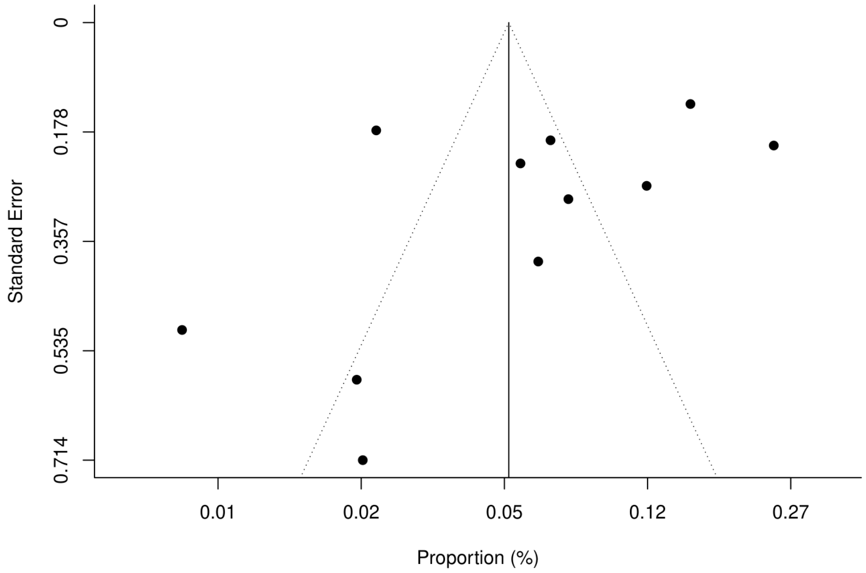

Figure 27. Funnel plot. Neurological and neuromuscular (Headache) by Egger's method

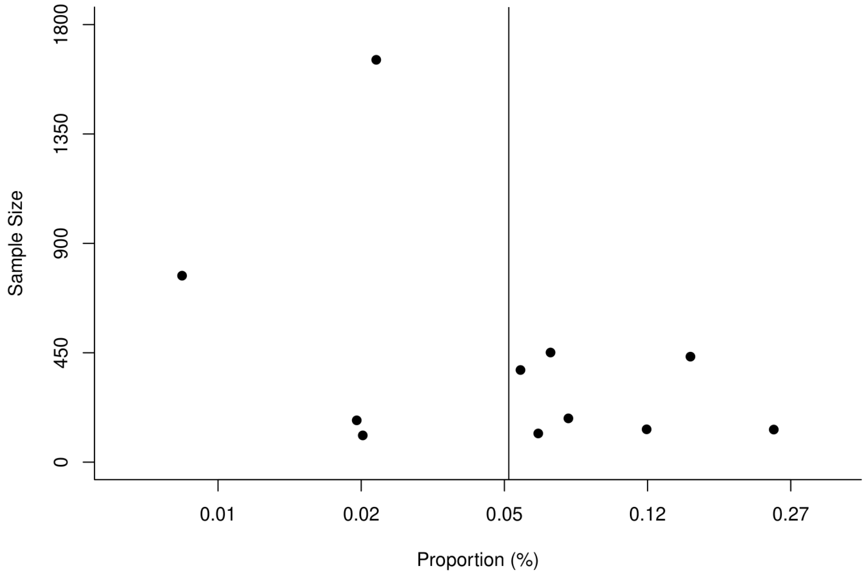

Figure 28. Neurological and neuromuscular (Headache) by Peter's method

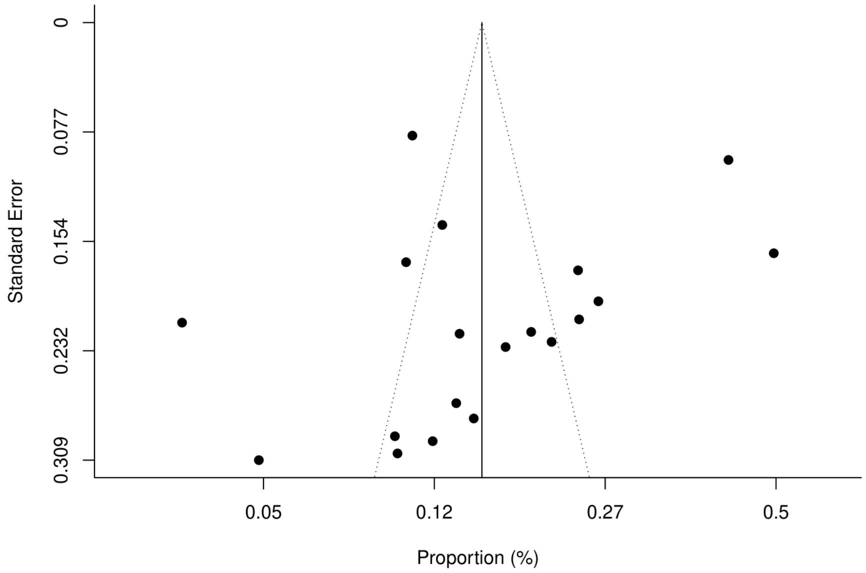

Figure 29. Funnel plot. Neurological and neuromuscular (Smell disturbance) by Egger's method

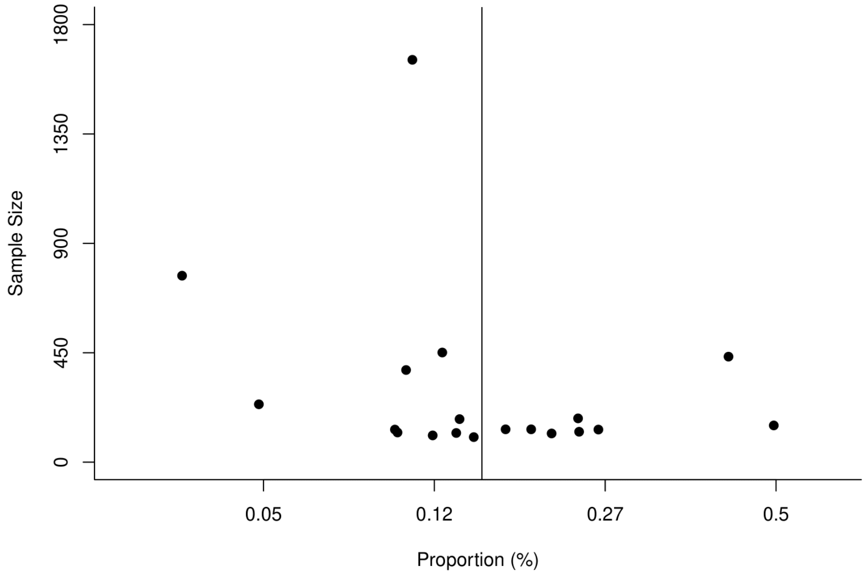

Figure 30. Neurological and neuromuscular (Smell disturbance) by Peter's method

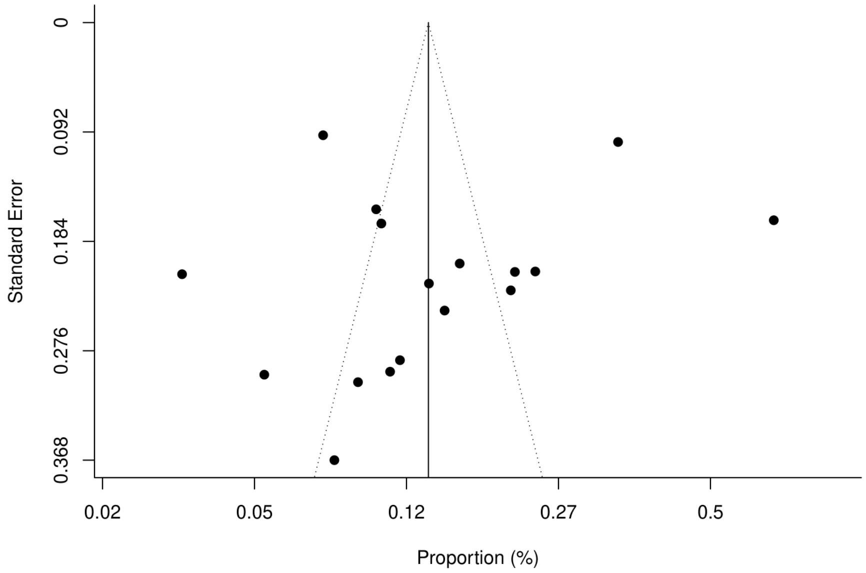

Figure 31. Funnel plot. Neurological and neuromuscular (Taste disturbance) by Egger's method

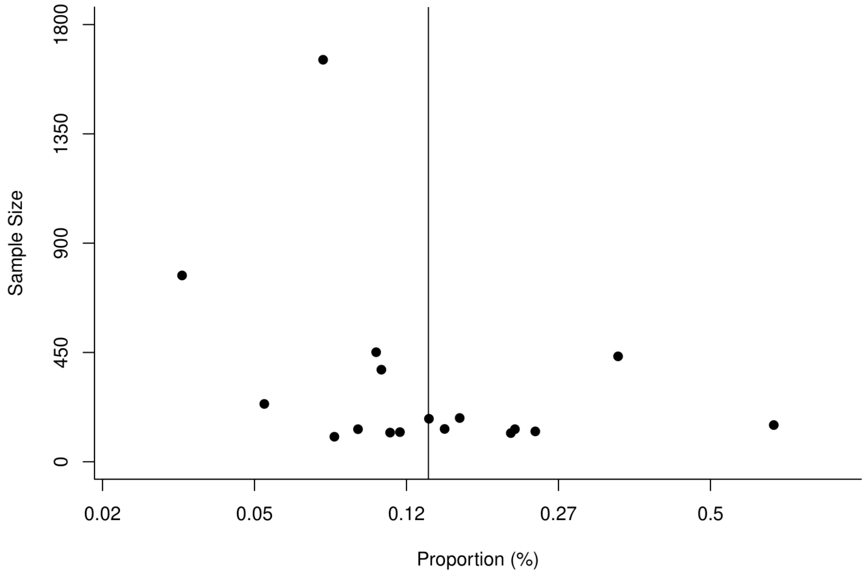

Figure 32. Funnel plot. Neurological and neuromuscular (Taste disturbance) by Peter's method

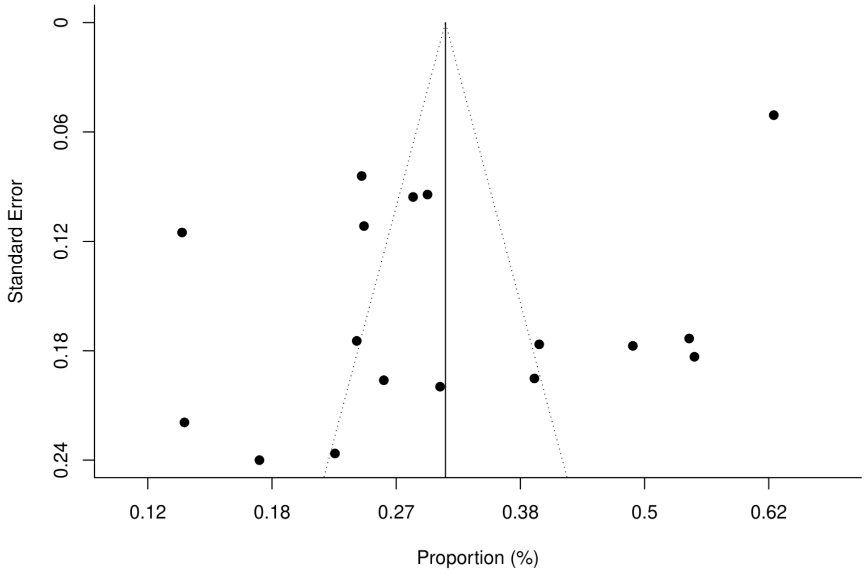

Figure 33. Funnel plot. Systemic (Fatigue) by Egger's method

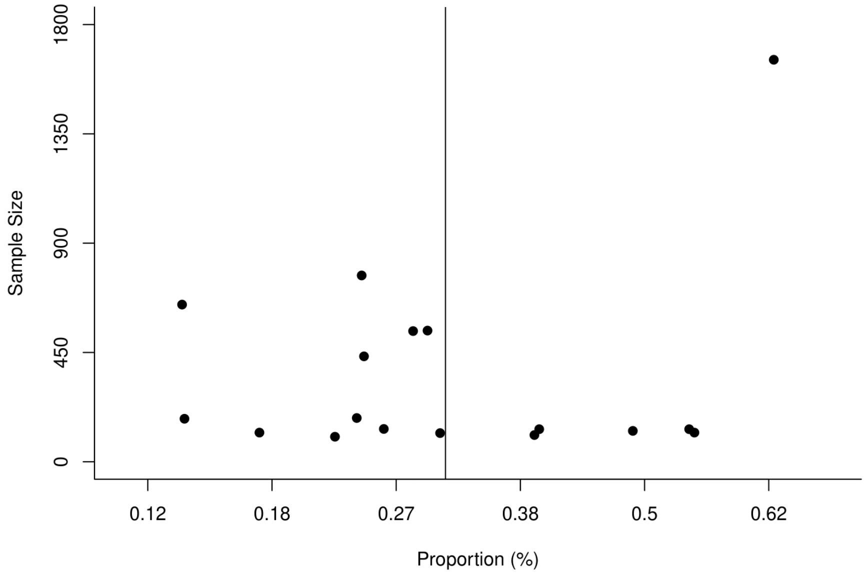

Figure 34. Funnel plot. Systemic (Fatigue) by Peter's method

Supplement 14: Risk factors

Study	Category	Risk factor	Associated with	Method	P Value/ CI
Nguyen et al.	Sex	Female sex	Persistent symptoms	Chi-squared or the Fisher exact test	p = 0.02
Mazza et al.	Sex	Female sex	Persistence of depressive symptomatology	Multivariate GLM analysis	(Wilks' λ = 0.92; F = 5.76; p = 0.003)
	Comorbidities	Previous psychiatric diagnosis			(Wilks' λ = 0.93; F = 5.29; p = 0.006)
	Severity	Presence of psychopathology at one-month			(Wilks' λ = 0.82; F = 15.16; p < 0.001)
Parentes-Arias et al.	Age	<60 years	Olfactory dysfunction	Multivariable-adjusted ORs	p = 0.028
	Sex	Female sex			p = 0.003
	Comorbidities	1 comorbidity			p = 0.031
Xiong et al.	Sex	Female sex	Covid-19 sequelae	Multivariable logistic regression model	Physical decline/fatigue (p < 0.01)<br>Postactivity polypnoea (p= 0.04)<br>Alopecia (p < 0.01)
	Severity	Dyspnea during hospitalisation	Physical decline/fatigue, postactivity polypnoea and resting heart rate increases	Univariate analysis	Physical decline/fatigue (p=.02)<br>Postactivity polypnoea (p=.01)<br>Resting heart rate increases (p=.01)
Sykes et al.	Sex	Female sex	Persistent symptoms	Chi-Square and Mann–Whitney U testing	Anxiety (p=0.001),low mood (p=0.031), myalgia (p=0.022), fatigue (p=0.004), sleep disturbance (p=0.009), and memory impairment (p=0.001)
Taboada et al.	Age	Age	Limitations in the functional status (grade II-IV of PCSF)	Multivariate logistic regression model	(OR = 2.600, 95% CI: 1.192–5.671)
	Severity	Length of hospital stay			(OR = 1.049, 95% CI: 1.009–1.090)
	Severity	Admission to ICU / mechanical ventilation			P < 0.001
Qu et al.	Sex	Female sex	Poor QoL scores	Logistic regression	(OR: 1.79, 95% CI: 1.04–3.06)
	Age	Older age (≥60 years)			(OR: 2.44, 95% CI: 1.33–4.47)
	Severity	Physical symptom after discharge			(OR: 40.15, 95% CI: 9.68–166.49)
Einvik et al.	Sex	Female sex	Symptoms of post-traumatic stress	Multivariable linear regression	NR
	Ethnicity	Born outside Norway			

	Severity	Dyspnoea during COVID-19			
Gherlone et al.	Comorbidities	COPD	Dry mouth	Multivariable analysis	(OR= 9.10, 95% CI: 1.8 -68.49)
Stavem et al.	Severity	Number of symptoms (10–23)	Symptoms at follow-up	Multivariable negative binomial regression analysis	(OR= 4.16, 95% CI:2.57 to 6.72, p<0.001)
	Comorbidities	≥2			(OR=2.52, 95%CI: 1.58 to 4.02, p<0.001)
Baricich et al.	Severity	ICU admission	Physical impairment	Multivariable logistic regression model	(OR: 3.1, 95%CI: 1.3-7.9, p=0.01)
	Age	Age	walking ability (SPPB)		p <0.02
	Comorbidities	Number or comorbidities	walking ability (SPPB)<br>2MWT		p <0.01<br>p <0.04
	Sex	Male gender	SPPB total score		p <0.01
Jacobson et al.	Ethnicity	Latin ethnicity	lower expected 6-MWT	Multivariate analysis	(-7.40 [-11.55-{-3.25}], p=0.001
	Comorbidities	BMI			(-0.52 [-0.81-{-0.22}], p=0.001)
	Severity	Persistence of symptoms at follow up	Shortness of breath		P=0.004
Petersen et al.	Age	Individuals in age group 50-66 compared with the youngest groups:<br>0-17 years<br>18-34 years	Persistent symptoms	Age-stratified analysis	p=0.003<br>p=0.001
Alharthy et al.	Severity	Increased incidence of dyspnoea and fever prior to hospital admission, decreased ICU admission PaO2/FiO2 ratio < 100, longer duration of mechanical ventilation, increased inflammatory biomarkers such as lactate dehydrogenase, ferritin, and D-dimers on ICU admission, and significant lung abnormalities detected by LUS	Persistent symptoms	Continuous variables using the Wilcoxon rank sum or the student's t-test. Categorical variables were examined using the Fisher's exact test or the Chi square test	p < 0.05

Anastasio et al.	Severity	Pneumonia and ARDS	Shortness of breath	Pearson's correlation coefficient and Cox regression were used	Patients who developed ARDS showed higher SBP (p=0.05) and DBP (p=0.02) and lower SpO2 during 6 MWT (p=0.004), FVC (p=0.004) and TLC (p<0.001). Patients without ARDS showed higher SR (p<0.001), RV (p<0.001), TLC (p<0.001) and RV/TLC (p=0.05).
Han et al.	Severity	Higher baseline CT lung involvement score (>=18 out of a possible score of 25)	Fibrotic-like changes in the lung at 6 months	Multivariate analysis	(OR: 4.2, 95%CI: 1.2-14)
Blanco et al.	Severity	Severity of the disease	DLCO <80% and a lower serum lactate dehydrogenase level	Multivariate analysis	DLCO<80% (OR 5.92; 95%CI 2.28–15.37; p < 0.0001) Serum lactate dehydrogenase (OR 0.98; 95%CI 0.97–0.99)
Lerum et al.	Severity	ICU admission	Persistent CT abnormalities and problems in usual activities	Mann–Whitney U-tests or Chi-squared tests	p=.031
Bellan et al.	Severity	Higher DLCO	Decreased risk of physical impairment	Univariate analysis and logistic regression models	(OR, 0.96 [95% CI, 0.94-0.98]; P < .001)
	Comorbidities	COPD	Increase risk of physical impairment		(OR, 12.70 [95% CI, 1.41-114.85]; P = .02)
Sonnweber et al.	Severity	Age, gender, and pre-existing diseases such as cardiovascular diseases, pulmonary diseases, diabetes mellitus type 2, and malignancy	Persistence of symptoms, patient performance status, and CT findings at follow-up	Friedman's or Wilcoxon signed-rank test	p=0.042 to p<0.001
Mendez et al.	Sex	Female sex	Impaired DLCO	Linear regression analysis	0.002
	Severity	ICU patients	Pulmonary embolism		p<0.001
		D-dimer levels	Impaired DLCO		p= 0.011
Blanco et al.	Severity	Lower serum LDH levels	Impaired DLCO	Multivariate analysis	OR 0.98; 95% CI 0.97-0.99; p 0.002
Qin et al.	Severity	Higher TSS of the chest and ARDS lymphocyte count, MPA diameter on admission and ARDS	Impaired DLCO	Univariable analysis	TSS>10.5 (OR: 10.5; 95%CI: 2.5-44.1; P=0.001) ARDS (OR: 4.6; 95%CI: 1.4-15.5; P=0.014)
		Long hospital stay	Lung sequelae		

Rass et al.	Severity	ICU patients	New neurological diseases	Chi-square or Kruskal-Wallis test	P=0.001
	Age	Elderly	Neurological signs	NR	NR
Weng et al.	Severity	Less severe (Lower frequency of supplemental oxygen therapy (79% vs 94%; p=0·016), and lower frequency of ICU admission	Gastrointestinal sequelae	Univariable and multivariable logistic regressions	p=0·016
		Treated more often with proton pump inhibitors (PPIs) and corticosteroids and were less frequently treated with enteral nutrition			PPI (p=0.000)<br>Corticosteroids (p=0.024)<br>Enteral nutrition (p=0.007)
Arnold et al.	Severity	Severe cases	Lower physical score	Mann Whitney-U and Kruskal Wallis tests for continuous data and Fisher's exact test or Chi-squared testing for categorical data.	NR
Sibila et al.	Sex	Male gender	Spirometric abnormalities 3 months after discharge,=	NR	Reduced FEV1: (76.9% vs 51.2%, p = 0.005)<br>Reduced FVC: (76.3% vs 51.6%, p = 0.008)
	Comorbidities	Cardiovascular disease and diabetes			Reduced FEV1:<br>Cardiovascular disease (34.2% vs 9.4%, p = 0.001)<br>Diabetes (28.9% vs 12%, p = 0.02)<br>Reduced FVC:<br>Cardiovascular disease (29.7% vs 11.0%, p = 0.009)
Huang et al.	Severity	Participants with severity scale 5–6	Higher risk of lung diffusion impairment, anxiety or depression, and fatigue or muscle weakness	Multivariable analysis	OR 4·60 (95% CI 1·85–11·48) for diffusion impairment, OR 1·77 (1·05–2·97) for anxiety or depression, and OR 2·69 (1·46–4·96) for fatigue or muscle weakness
	Sex	Female sex			

ARDS: Acute respiratory distress syndrome; BMI: Body mass index; CT: Computerised Topography; DCLO: diffusing capacity for carbon monoxide; ICU: Intensive care unit; LDH: Lactate dehydrogenase; LUS: lung ultrasound; MWT: minute walking test; NR: Not reported; OR: Odds Ratio; PCSF: post covid functional status; QoL: Quality of life; SPPB: Short Physical Performance Battery test; TSS: Toxic shock syndrome
